# Chromosome rearrangements, recombination suppression, and limited segregation distortion in hybrids between Yellowstone cutthroat trout (Oncorhynchus clarkii bouvieri) and rainbow trout (O. mykiss)

**DOI:** 10.1186/1471-2164-14-570

**Published:** 2013-08-22

**Authors:** Carl O Ostberg, Lorenz Hauser, Victoria L Pritchard, John C Garza, Kerry A Naish

**Affiliations:** 1 U.S. Geological Survey, Western Fisheries Research Center, 6505 NE 65th Street, Seattle, WA 98115, USA; 2 School of Aquatic and Fishery Sciences, University of Washington, 1122 NE Boat Street, Box 355020, Seattle, WA 98105, USA; 3 Southwest Fisheries Science Center, National Marine Fisheries Service and University of California, Santa Cruz, 110 Shaffer Road, Santa Cruz, CA 95060, USA

## Abstract

**Background:**

Introgressive hybridization is an important evolutionary process that can lead to the creation of novel genome structures and thus potentially new genetic variation for selection to act upon. On the other hand, hybridization with introduced species can threaten native species, such as cutthroat trout (*Oncorhynchus clarkii*) following the introduction of rainbow trout (*O. mykiss*). Neither the evolutionary consequences nor conservation implications of rainbow trout introgression in cutthroat trout is well understood. Therefore, we generated a genetic linkage map for rainbow-Yellowstone cutthroat trout (*O. clarkii bouvieri*) hybrids to evaluate genome processes that may help explain how introgression affects hybrid genome evolution.

**Results:**

The hybrid map closely aligned with the rainbow trout map (a cutthroat trout map does not exist), sharing all but one linkage group. This linkage group (RYHyb20) represented a fusion between an acrocentric (Omy28) and a metacentric chromosome (Omy20) in rainbow trout. Additional mapping in Yellowstone cutthroat trout indicated the two rainbow trout homologues were fused in the Yellowstone genome. Variation in the number of hybrid linkage groups (28 or 29) likely depended on a Robertsonian rearrangement polymorphism within the rainbow trout stock. Comparison between the female-merged F_1_ map and a female consensus rainbow trout map revealed that introgression suppressed recombination across large genomic regions in 5 hybrid linkage groups. Two of these linkage groups (RYHyb20 and RYHyb25_29) contained confirmed chromosome rearrangements between rainbow and Yellowstone cutthroat trout indicating that rearrangements may suppress recombination. The frequency of allelic and genotypic segregation distortion varied among parents and families, suggesting few incompatibilities exist between rainbow and Yellowstone cutthroat trout genomes.

**Conclusions:**

Chromosome rearrangements suppressed recombination in the hybrids. This result supports several previous findings demonstrating that recombination suppression restricts gene flow between chromosomes that differ by arrangement. Conservation of synteny and map order between the hybrid and rainbow trout maps and minimal segregation distortion in the hybrids suggest rainbow and Yellowstone cutthroat trout genomes freely introgress across chromosomes with similar arrangement. Taken together, these results suggest that rearrangements impede introgression. Recombination suppression across rearrangements could enable large portions of non-recombined chromosomes to persist within admixed populations.

## Background

The widespread occurrence of hybridization has been a catalyst for intensive study in evolutionary biology and has provided rich opportunities for investigating genome evolution, adaptation, speciation, reproductive isolation, and hybrid fitness [1-3]. Hybridization is a natural evolutionary process, contributing to the diversification of plants and animals [4,5]. When populations hybridize and progeny are viable and fertile, genomes introgress and produce recombined chromosomes. Introgression breaks down linkage associations and generates novel gene combinations which may have fitness consequences [2]. However, hybridization can also have significant conservation implications, leading to the formation of hybrid swarms and extinction [6,7]. In these cases, introgression may interfere with epistatic interactions by disrupting local adaptations and breaking down co-adapted gene complexes. Regardless of the consequences, understanding the genome processes that affect introgression is fundamental to understanding hybrid genome evolution.

Genetic linkage maps provide a means for investigating genome evolution and function, and have contributed to an improved understanding of hybridization and introgression [8-10]. They have provided empirical evidence that chromosome rearrangements can act as barriers to gene flow by suppressing recombination between rearranged chromosomes [11-13]. They can be used to identify segregation distortion (loci that deviate from Mendelian inheritance patterns), which may indicate the presence of fitness-linked loci or genetic incompatibilities [14]. When applied to introgressed populations, linkage maps have identified specific genomic regions that might be important for providing adaptive fitness advantages [9,15]. Finally, they provide a framework for detecting quantitative trait loci (QTL), enabling identification of genomic regions associated with ecological, evolutionary, or physiological processes within hybrids and parental species [10,16,17].

Genome maps for salmonid fishes show signatures of two significant events: genome duplication and chromosome rearrangements [18-21]. The first significant event, genome duplication, is thought to have occurred through autopolyploidy approximately 25–100 million years ago, resulting in an ancestral tetraploid genome [22]. Rediploidization of the genome is occurring but is not complete [22]. This residual tetraploidy has two consequences exclusive to males. First, males form multivalents between homeologues during meiosis [23], suppressing crossing-over between homologues and reducing recombination rates compared to females [24]. Second, residual tetraploidy results in pseudolinkage between homeologous chromosomes in males [23], producing statistical rather than physical linkage [24]. The second significant event, chromosome rearrangements, has generated highly variable chromosome numbers among salmonid species [25]. Although chromosome numbers differ among species, chromosome arm numbers have remained relatively constant because rearrangements have primarily been of the Robertsonian type between acrocentric and metacentric chromosomes. Robertsonian rearrangements are translocations that involve centric fusion or fission between chromosome arms, causing a change in chromosome number but not chromosome arm number.

Rainbow trout (*Oncorhynchus mykiss*) and cutthroat trout (*O. clarkii*) are two salmonid species that inhabit western North America. Rainbow and cutthroat trout are sister species and shared a common ancestor approximately 3 million years ago [26]. Despite karyotypic differences between the species, Robertsonian rearrangements have maintained the same number (n = 104) of diploid chromosome arms [27,28]. Similarity in chromosome arm number between the two species could be an important factor that enables the species to readily hybridize and produce viable and fertile progeny. In fact, where non-indigenous rainbow trout have been introduced into indigenous cutthroat trout habitats, introgressive hybridization has led to hybrid swarms and extinction of local cutthroat trout populations, and has thus become a major conservation concern [29]. Although introgression between rainbow and cutthroat trout is well documented, it is unknown how introgression affects the genomic architecture of hybrids and thus their subsequent evolution. For example, karyotypic differences between rainbow and cutthroat trout could affect hybrid genome evolution by suppressing recombination, hindering gene flow, and generating linkage disequilibrium [10,11,13,30]. As a result, the ability of invading alleles to become established within a host genome could be influenced by the presence of chromosome rearrangements. In addition, reduced recombination between rearranged chromosome segments could prevent disruption of co-adapted gene complexes [31,32]; enabling these adaptations to persist within hybrid populations, which could ultimately affect hybrid fitness.

Here, we present the first hybrid genetic linkage map between two introgressing salmonid species, rainbow trout and Yellowstone cutthroat trout (*O. c. bouvieri*). Rainbow trout (RBT) have 58–64 diploid chromosomes depending on the chromosome race [28] whereas Yellowstone cutthroat trout (YCT) have 64 diploid chromosomes [27]. We generated F_2_ hybrids between RBT and YCT and developed a F_1_ hybrid linkage map to investigate the genomic consequences of introgression. The objectives for constructing the F_1_ hybrid linkage map were to 1) determine if linkage groups were conserved between the F_1_ map and existing RBT maps, 2) determine if introgression suppressed recombination, and 3) estimate the prevalence of segregation distortion in the F_1_ hybrid map. Our hybrid linkage map has application to conservation and management of indigenous cutthroat trout subspecies because it localizes species-specific markers to linkage groups and identifies genomic regions where recombination is suppressed, both of which may assist resource managers in determining accurate estimates of RBT admixture throughout the native cutthroat trout range. It will also have application for identifying QTL associated with species-specific traits. Finally, it can be used as a baseline for future comparative mapping studies in rainbow-cutthroat trout hybrids.

## Results

### Hybrid linkage map

After removing markers that were heterozygous for the same alleles in both parents, 310 microsatellite loci, 72 single nucleotide polymorphisms (SNPs), and one species-specific insertion/deletion (indel) were mapped in YCT-RBT F_1_ hybrids using two families (Family, 54 progeny; Family 2, 53 progeny) (Additional file 1, Worksheets 2 – 8; Additional files 2 and 3). We identified a total of 28–29 linkage groups in the sex-merged map for the hybrids (Table 1) and have designated hybrid linkage groups as RYHyb (rainbow-Yellowstone hybrid). Comparisons to RBT chromosomes [21,33] revealed that all linkage groups identified in the F_1_ hybrids were syntenic with and had similar marker orders to specific RBT chromosomes, and therefore we defined each specific linkage group with respect to its homologous RBT chromosome, except RYHyb20 (a chromosome fusion) and RYHyb28 (RBT sex chromosome homologue) (Table 1).

**Table 1:** **Hybrid linkage groups and alignment to rainbow trout chromosome (Omy) and linkage group (RT) arms (*p*and*q*)**

**Hybrid linkage group**	**Inferred linkage group structure**	**Omy arms**	**RT arms**
RYHyb01	Metacentric	Omy01 (p, q)	RT6 (p, q)
RYHyb02	Metacentric	Omy02 (p, q)	RT27 (p, q)
RYHyb03	Metacentric	Omy03 (p, q)	RT31 (p, q)
RYHyb04	Metacentric	Omy04 (p, q)	RT24 (p, q)
RYHyb05	Metacentric	Omy05 (p, q)	RT8 (p, q)
RYHyb06	Metacentric	Omy06 (p, q)	RT10 (p, q)
RYHyb07	Metacentric	Omy07 (p, q)	RT12 (p, q)
RYHyb08	Metacentric	Omy08 (p, q)	RT23 (p, q)
RYHyb09	Metacentric	Omy09 (p, q)	RT21 (p, q)
RYHyb10	Metacentric	Omy10 (p, q)	RT20 (p, q)
RYHyb11	Metacentric	Omy11 (p, q)	RT19 (p, q)
RYHyb12	Metacentric	Omy12 (p, q)	RT9 (p, q)
RYHyb13	Metacentric	Omy13 (p, q)	RT2 (p, q)
RYHyb14	Metacentric	Omy14 (p, q)	RT3 (p, q)
RYHyb15	Metacentric	Omy15 (p, q)	RT7 (p, q)
RYHyb16	Metacentric	Omy16 (p, q)	RT22 (p, q)
RYHyb17	Metacentric	Omy17 (p, q)	RT29 (p, q)
RYHyb18	Metacentric	Omy18 (p, q)	RT16 (p, q)
RYHyb19	Metacentric	Omy19 (p, q)	RT14 (p, q)
RYHyb20	Metacentric	Omy20 (p, q)_Omy28 (q)	RT17 (p, q)_RT13 (q)
RYHyb21	Metacentric	Omy21 (p, q)	RT15 (p, q)
RYHyb22	Metacentric	Omy22 (p, q)	RT5 (p, q)
RYHyb23	Acrocentric	Omy23 (q)	RT30 (q)
RYHyb24	Acrocentric	Omy24 (q)	RT26 (q)
RYHyb25^1^	Acrocentric	Omy25 (q)	RT4 (q)
RYHyb25_29^1^	Metacentric	Omy25 (q)_Omy29 (q)	RT4 (q)_RT25 (q)
RYHyb26	Acrocentric	Omy26 (q)	RT18 (q)
RYHyb27	Acrocentric	Omy27 (q)	RT11 (q)
RYHyb28	Acrocentric	Omysex (q)	RT1 (q)
RYHyb29^1^	Acrocentric	Omy29 (q)	RT25 (q)

^1^ Female 1 and Male 2 were homozygous for the acrocentric chromosomes RYHyb25 and RYHyb29. Female 2 and Male 1 were heterozygotes for the polymorphism, comprising the fused metacentric RYHyb25_29 and the acrocentric chromosomes RYHyb25 and RYHyb29.

**Figure 1:**
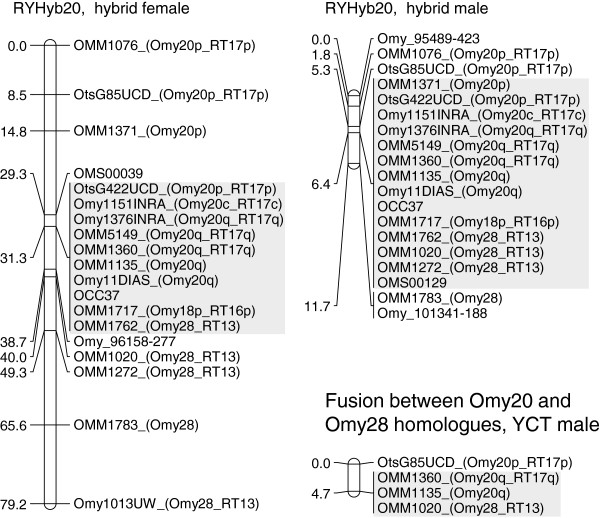
**Hybrid linkage group RYHyb20 showing the fusion between rainbow trout chromosomes Omy20 and Omy28.** Female- and male-merged hybrid maps are shown. The grey shaded area represents a block of non-recombining loci spanning the *p*-arm, centromere, and *q*-arm of Omy20 and the acrocentric Omy28. The fusion between the Omy20 and Omy28 homologues in a male Yellowstone cutthroat trout (YCT) map, identified by applying four loci in common to the hybrid and rainbow trout maps, is also shown. The rainbow trout chromosome (Omy), linkage group (RT), and chromosome arm (*p*, *q*) or centromere (c) location for each locus mapped in rainbow trout by Guyomard *et al.* (2006) and Rexroad *et al.* (2008) is indicated. Map distances are in centiMorgans.

We found evidence for a fusion between two RBT chromosomes in the hybrids. Rainbow trout chromosomes Omy20 (a metacentric chromosome in RBT) and Omy28 (an acrocentric chromosome in RBT), were fused into a single linkage group, RYHyb20, in both sexes (Figure 1). Several loci mapping to Omy20 and Omy28 in RBT [21,33] did not recombine in the F_1_ hybrids, indicating that a major portion of RYHyb20 was inherited as a single, non-recombining block of markers. Additional mapping in a male YCT (48 progeny) indicated that the Omy20 and Omy28 homologues were fused in YCT.

**Figure 2:**
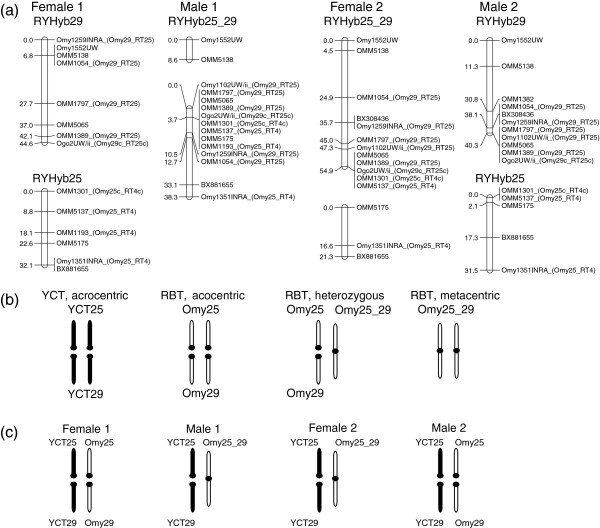
**Robertsonian polymorphism between RYHyb25 and RYHyb29. (a)** Linkage groups RYHyb25 and RYHyb29 represent acrocentric chromosomes in Female 1 and Male 2 while RYHyb25_29, a metacentric chromosome, represents a fusion between the two acrocentric chromosomes in Male 1 and Female 2. Chromosome (Omy), linkage group (RT), and putative centromere (c) location for each locus mapped in Guyomard *et al.* (2006) is indicated. Map distances are in centiMorgans. **(b)** Possible diploid chromosome constitutions for the Omy25 and Omy29 Robertsonian polymorphism in the rainbow trout (RBT) parent stock (acrocentric, 2 N = 60; heterozygous, 2 N = 59; or metacentric 2 N = 58) and the Yellowstone cutthroat trout (YCT) homologues (YCT25 and YCT29). RBT chromosomes are in white and YCT chromosomes are in black. **(c)** Inferred diploid chromosomal constitutions for F_1_ hybrid parents. YCT crossed by RBT with either the acrocentric or heterozygous polymorphism would yield RYHyb25 and RYHyb29 in Female 1 and Male 2, and YCT crossed by RBT with either the metacentric or heterozygous polymorphism would yield RYHyb25_29 in Male 1 and Female 2.

Linkage groups RYHyb25 and RYHyb29 (homologues to Omy25 and Omy29, respectively, in RBT) differed in arrangement among parents. The distal mapping loci OMM1301 (on RYHyb25) and Ogo2UW/ii (on RYHyb29) were not linked in Female 1 and Male 2, indicating two independent linkage groups (Figure 2A). However, there was no recombination between these loci in Female 2 and Male 1, suggesting RYHyb25 and RYHyb29 were fused in these parents (noted as RYHyb25_29). The recombination estimate between OMM1301 and OMM1797 in the progeny of a male YCT (*Θ* = 0.46, LOD = 0.03) confirmed that the RYHyb25 and RYHyb29 homologues are not fused in YCT. We found a similar recombination estimate between the same loci in Male2 (*Θ* = 0.45, LOD = 0.08), but not in Male 1 (*Θ* = 0.0, LOD = 12.94).

Marker order was largely conserved among parent-specific and between female- and male-merged maps (Additional file 1, Worksheets 2 – 8; Additional files 2 and 3). However, we observed inconsistent marker order among parent-specific maps for RYHyb25 and RYHyb29 and, therefore, these two linkage groups could not be merged within sex. Further, marker order differed between sexes for distally mapping loci at RYHyb13p, RYHyb19, and RYHyb24. Loci with different order between sexes were removed prior to generating the sex-merged map, and removal did not appear to alter marker order within linkage groups. Marker order differences could be due to marker informativeness within families, insufficient chromosome coverage, or reduced recombination in males.

### Assignment of species diagnostic markers to linkage groups

We assigned 114 diagnostic species markers (97 SNPs, 13 indels, and 4 restriction fragment length polymorphisms (RFLPs)) to specific linkage groups (Additional file 1, Worksheets 9). Specific mapping locations within linkage groups could not be determined because the F_1_ parents were heterozygous at these loci. One indel, OCC-37, was polymorphic within YCT and mapped to a specific location within RYHyb20. All hybrid linkage groups contained at least one species-specific diagnostic marker. Linkage groups RYHyb21 and RYHyb11, both metacentric chromosomes, were each assigned a single diagnostic species marker indicating that only one of the two chromosome arms in both linkage groups had a species-specific marker assigned.

### Homeologies

Using duplicated loci, we observed six homeologous hybrid linkage groups (RYHyb12q/RYHyb13q, RYHyb13p/RYHyb17p, RYHyb14p/RYHyb29, RYHyb15q/RYHyb21q, RYHyb10q/RYHyb19p, and RYHyb06p/RYHyb26) that have been previously identified within RBT [34]. We also observed one homeologous pairing that has not been observed in rainbow trout (RYHyb03 centromere region/RYHyb22q). Additionally, for 10 other duplicated loci, only one homeologue could be scored confidently. Linkage groups RYHyb13q and RYHyb19p each contained two of these loci, and RYHyb02p, RYHyb02 centromere region, RYHyb10q, RYHyb10 centromere region, RYHyb18p, and RYHyb21q each contained one of these loci.

### Pseudolinkage

We found eight pseudolinkage groups exclusive to both male maps (RYHyb01/RYHyb23, RYHyb02/RYHyb03, RYHyb06/RYHyb26, RYHyb07/RYHyb18, RYHyb10/RYHyb19, RYHyb15/RYHyb21, RYHyb12/RYHyb13, and RYHyb13/RYHyb17). Pseudolinkage was represented in males by statistical linkage between markers that mapped to independent linkage groups in the two female maps. All cases of pseudolinkage were between chromosomes identified as homeologous within RBT [34].

### Recombination rates

Females within both families had a significantly higher recombination rate across the genome than males. The female to male recombination ratio in Family 1 and Family 2 was 6.92 (*P* < 0.001, G-test) and 5.65 (*P* < 0.001, G-test), respectively (Table 2). Females had significantly higher recombination rates across each linkage group than males within at least one family (*P* < 0.001, G-test), with the following exceptions. Female and male recombination rates were not different across RYHyb24, and the female to male recombination ratios could not be estimated across RYHyb11, RYHyb14, and RYHyb23 because male pairwise recombination values were zero for all corresponding female pairwise comparisons. The recombination rate was not different between mapping parents of the same sex across the genome.

**Table 2:** Map distances, in centiMorgans (cM), in female and male merged hybrid maps, female to male recombination ratios (F:M), and significant allelic segregation distortion across 25 cM intervals for each linkage group (sliding window analysis)

**Linkage group**	**Female map distance (cM)**	**Male map distance (cM)**	**Family 1 F:M ratio**	**Family 2 F:M ratio**	**Significant number of 25 cM intervals**^**1**^
RYHyb01	89.4	3	14.25*	51.17*	↓F1(1), ↓F2(2)
RYhyb02	82.9	19.3	5.22*	35.00*	↓F2(2)
RYHyb03	88	10.8	21.64*	3.63*	↓F1(1)
RYHyb04	106.9	4.3	30.29*	11.31*	
RYHyb05	73.9	44.1 (10.8 + 33.3)^2^	-	2.78*	↓M2(2)
RYHyb06	83	10.2	-	12.25*	
RYHyb07	59	23.9	-	3.03*	↓M2(1)
RYHyb08	84.5 (21.8 + 62.7)^2^	33.8	16.89*	4.66*	↑M1(1)
RYHyb09	88.5	35.1	3.32*	3.59*	↑M1(2), ↑M2(1)
RYHyb10	89.7	3.3	25.58*	-	
RYHyb11	16.7	2.3	-	-	↑M1(1)
RYHyb12	77.4	27.5	17.37*	6.24*	↓F1(3)
RYHyb13	85.9	13.3	8.00*	-	↓F2(3)
RYHyb14	46.4 (46.4 + 0)^2^	1.4	-	-	
RYHyb15	66.5	22	6.29*	13.13*	
RYHyb16	83	39.2	6.21*	4.89*	
RYHyb17	97.4	7	2.25*	-	
RYHyb18	92.9	5.1	27.90*	3.5	↑M2(1)
RYHyb19	79.5	24	8.81*	-	↑F1(2)
RYHyb20	79.2	11.7	7.81*	7.58*	
RYHyb21	58	15.8	8.01*	23.30*	
RYHyb22	67.8	56.3	2.30*	1.78*	↑F1(2)
RYHyb23	64.6	0	-	-	
RYHyb24	41.8	22.6	-	0.67	
RYHyb25	32.1	31.5	1.21*	0.79	
RYHyb25_29	76.1 (54.8 + 21.3)^2^	46.9 (8.6 + 38.3)^2^	1.91*	1.54*	
RYHyb26	44.2	13.3	0.67	22.40*	↑M2(1)
RYHyb27	39.4	1.8	29.60*	-	↓F2(1)
RYHyb28	56.2	7.9	9.90*	6.75*	
RYHyb29	44.6	40.3	3.60*	1.60*	
Genome wide^3^	2019.1	518.35	6.92*	5.65*	

Dashed line (−) indicates that the recombination rate in the male parent was zero and therefore undefined.

*Significant G-tests at *P* < 0.001 following corrections for multiple tests.

^1^ Arrows indicate significantly greater (↑) or less (↓) YCT allele frequencies than expected within each parent–specific map (*F1* Female 1, *F2* Female 2, *M1* Male 1, and *M2* Male 2). The number in parentheses indicates the number of significant 25 cM intervals within a parent map.

^2^ Map distance is the product of the two groups in parentheses.

^3^ The genome wide map distance was calculated using the average map distance across RYHyb25 + RYHyb29 and RYHyb25_29 within each sex (i.e. ((*RYHyb*25 + *RYHyb*29) + *RYHyb*25 _ 29)/2).

We identified 13 linkage groups where recombination rates differed significantly between the female consensus F_1_ hybrid and RBT maps (Additional file 4). We treated RYHyb20 as two separate linkage groups, noted as RYHyb20(Omy20) and RYHyb20(Omy28), because Omy20 and Omy28 are not fused in RBT. Further, RYHyb25 and RYHyb29 represent a fusion/fission polymorphism in the female maps, and so we treated these groups as independent in Female 1 and used the RBT linkage group in Guyomard *et al.*[33] for comparison. We also treated RYHyb25_29 as a metacentric linkage group in Female 2 and used the map of Rexroad *et al.*[21] for comparison.

**Figure 3:**
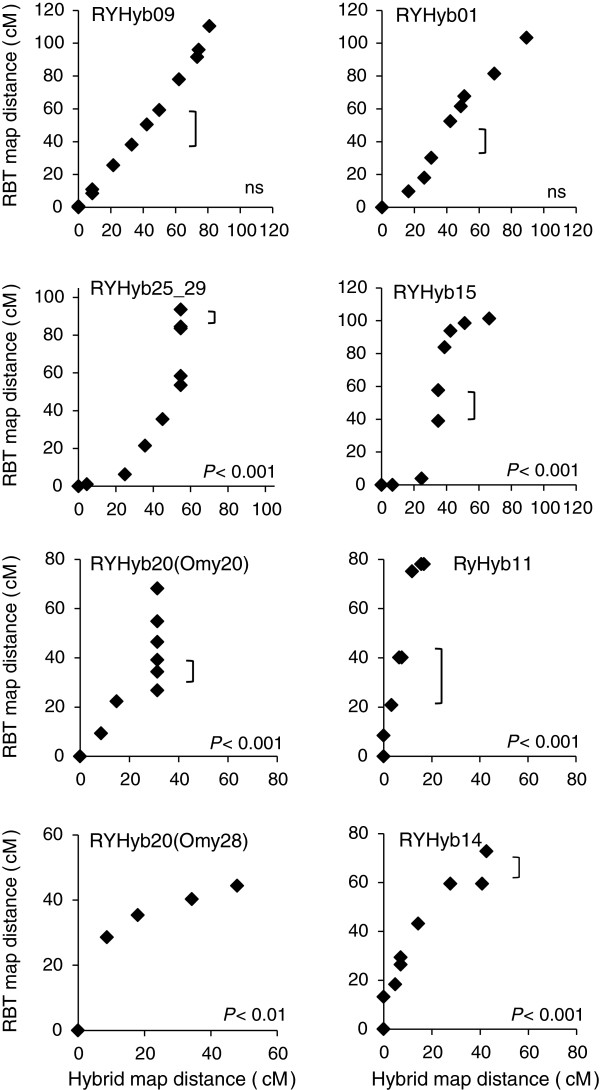
**Map distance comparison between the female-merged F**_**1 **_**hybrid map and the female consensus RBT map.** Selected linkage groups show map distance comparisons in centiMorgans (cM) across the same markers in the F_1_ hybrid map (X-axis) and the rainbow trout (RBT) map (Y-axis). Black diamonds indicate mapped markers (*p* to *q* orientation) and brackets indicate putative centromere locations for metacentric linkage groups identified in Guyomard *et al.* (2006). For acrocentric linkage groups, the marker closest to the centromere is plotted at zero. Significant differences in recombination (*P*-values) between the female-merged F_1_ and consensus RBT female maps across linkage groups are indicated (ns, not significant). See Additional file 4 for comparisons across all linkage groups.

We found five instances of suppressed and one instance of elevated recombination distance across large numbers of loci within several linkage groups in the female hybrid map relative to the female RBT map (Additional file 4) (Figure 3). All instances of recombination suppression mapped across centromeres. Linkage group RYHyb11, a metacentric chromosome in RBT, was the shortest linkage group in the female hybrid map. The recombination distance across the hybrid map relative to the RBT map (relative hybrid:RBT recombination distance) was 0.21, indicating the recombination rate was suppressed across all loci mapped in RYHyb11. Linkage group RYHyb20(Omy20) had a relative hybrid:RBT recombination distance of 0.46. The reduction in map distance appeared to be due to six non-recombining loci in the hybrid map, whereas the same six loci mapped over 40 cM to the *p*- and *q*-arms in the RBT map. In contrast, the relative hybrid:RBT recombination distance on the other arm of the hybrid map, RYHyb20(Omy28), appeared elevated across the four loci closest to the telomere in the hybrid map (2.44). However, the recombination distance appeared suppressed in the hybrid map between the two markers closest to the centromere. Recombination suppression near the centromere was likely associated with the fusion to RYHyb20(Omy20). Linkage group RYHyb25_29 had a relative hybrid:RBT recombination distance of 0.59; however, we were unable to include the majority of the q-arm in the comparison. We observed five non-recombining loci in the hybrid map, whereas the same five loci mapped over 40 cM in the RBT. In contrast, the single-armed linkage groups RYHyb25 and RYHyb29 had relative hybrid:RBT recombination distance of 0.82 and 1.12, respectively. The reduced relative map distance in RYHyb25_29 compared to RYHyb25 and RYHyb29 suggests that the fusion between the two chromosome arms caused a reduction in the recombination rate across the centromere. Linkage group RYHYb15 had a relative hybrid:RBT recombination distance of 0.66. Although the hybrid map had a reduced recombination distance across the centromere compared to the RBT map, the recombination distance was greater in the hybrid map than in the RBT map near telomeres on both chromosome arms. Linkage group RYHyb14 had a relative hybrid:RBT recombination distance of 0.59; however, we were unable to include the majority of the q-arm in the comparison.

### Segregation distortion

**Figure 4:**
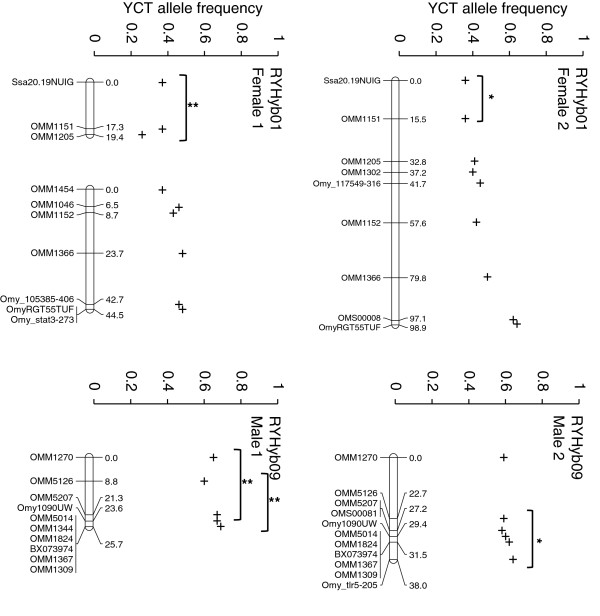
**Yellowstone cutthroat trout allele frequencies plotted against linkage maps.** Selected linkage groups show RYHyb01 in Females 1 and 2 and RYHyb09 in Males 1 and 2. A 25 centiMorgan sliding window was used to identify blocks of loci with Yellowstone cutthroat trout (YCT) allele frequencies that deviated significantly from expected frequencies. Linkage maps are in *p* to *q* orientation. * indicates *P* < 0.05 and ** indicates *P* < 0.01. See Additional file 2 for YCT allele frequencies within each mapping parent for all linkage groups.

Analyses based on a sliding window revealed that 27 of the 286 total 25 cM intervals among all parent-specific maps contained YCT allele frequencies that deviated significantly from Mendelian expectations (Additional file 2) (Table 2). The proportion of 25 cM intervals deviating significantly within each parent-specific map was as follows: Female 1, 9/104; Female 2, 8/107; Male 1, 4/35; and Male 2, 6/40. We found no consistent distortion in allele frequencies among all parents. However, both females showed a significant reduction in YCT allele frequencies in the same region on RHYb01p and both males showed a significant increase in YCT allele frequencies across both RYHyb09 arms (Figure 4). Interestingly, homeologues within pseudolinkage groups RYHyb07/RYHyb18 and RYHyb06/RYHyb27 had inverse proportions of YCT allele frequencies in Male 2. Linkage group RYHyb07 had significantly less, and homeologue RYHyb18 had significantly greater, YCT allele frequencies than expected. Similarly, RYHyb27 had significantly less, and the homeologue RYHyb06 had a trend for greater (although not significantly so), YCT allele frequencies than expected.

We found 15 linkage groups where genotypic segregation distortion occurred at *P* < 0.05 (Additional file 5). Approximately 6% of the total loci genotyped were distorted at this significance level. After applying B-Y FDR corrections for multiple tests, the observed genotypic frequencies differed significantly from expected frequencies within only four linkage groups (Additional file 5), and represented approximately 1.6% of the total loci genotyped. Linkage groups RYHyb09 and RYHyb18 contained loci with an excess of YCT/YCT genotypes, and RYHyb06 and RYHyb07 contained loci with an excess of RBT/RBT genotypes. Interestingly, none of the 309 loci mapped in common between the two families showed significant genotypic distortion within both families.

## Discussion

The genetic linkage maps established for YCT-RBT hybrids provide novel insights into the genomic consequences of introgression between RBT and YCT. Hybrid and RBT linkage groups were syntenic and had similar marker order, suggesting that RBT and YCT share chromosome arms. In addition, hybrids and RBT shared linkage groups, with one exception where a hybrid linkage group involved a fusion between a bi-armed metacentric and a single-armed acrocentric RBT chromosome. This result was confirmed by additional mapping in YCT. Comparison between hybrid and RBT female maps indicated that introgression suppressed recombination across several large chromosome segments. Two hybrid linkage groups showing recombination suppression (RYHyb20 and RYHyb25_28) contained different chromosome arrangements between YCT and RBT. Segregation distortion was generally limited and distortion patterns varied among parents and families, suggesting that few incompatibilities exist between RBT and YCT genomes. Taken together, these results suggest that RBT and YCT genomes freely introgress, with the exception that differences in chromosome rearrangements between the species could impede introgression across large portions of specific linkage groups.

### Chromosome rearrangements between RBT and YCT

The hybrid map is a product of differences in chromosome rearrangements between RBT and YCT, as well as mixed ancestry of the RBT source stock. Using data from karyotypes [27,28] and the hybrid map, we suggest that RBT and YCT differ by at least five species-specific chromosome rearrangements. We first consider the fusion between Omy28 and Omy20q in YCT. The most parsimonious explanation for the fusion between the metacentric Omy20 and the acrocentric Omy28 involves two rearrangements. The first rearrangement would have occurred prior to the Omy20-Omy28 fusion as a pericentric inversion of the entire q-arm of Omy20. This inversion would have resulted in Omy20 becoming an acrocentric chromosome and a reduction in the expected 52 haploid chromosome arms [27,28] to the observed 51 haploid arms in the hybrid map. The possibility that Omy20q could be inverted in YCT relative to RBT is supported in Chinook salmon (*O. tshawytscha*), as the Omy20 homologue in Chinook is an acrocentric chromosome and the segment homologous to Omy20q appears inverted compared to RBT (unpublished observations, K. Naish). The second rearrangement would have been a Robertsonian type involving centric fusion between the acrocentric Omy20 homologue and Omy28, conserving chromosome arm number. The three remaining differences may be explained by comparing acrocentric chromosome numbers between RBT (seven in the 60 chromosome race) and YCT (twelve). The seven RBT acrocentric chromosomes have been identified as such in the hybrid map, suggesting that they have an acrocentric YCT homologue. The remaining five YCT acrocentric chromosomes likely have homologs with RBT metacentric chromosomes. The third and fourth rearrangement differences probably represent centric fusion/fission events between two RBT metacentric and four of the five remaining YCT acrocentric chromosomes, which would not change the chromosome arm number. The fifth rearrangement difference may have involved a fission event within a RBT metacentric chromosome arm, generating the fifth acrocentric and a submetacentric chromosome in YCT. Fission within a RBT metacentric arm would gain one YCT chromosome arm and restore the 52 haploid chromosome arm number, countering the loss of a chromosome arm by the pericentric inversion.

The variable number of linkage groups identified in the hybrid maps (28 and 29) is likely due to mixed ancestry of the source RBT stock. Linkage analysis in YCT indicated that the Omy25 and Omy29 homologues each represent acrocentric linkage groups in YCT, but these chromosomes are known to be a Robertsonian polymorphism in RBT [28,34,35]. The Kamloops stock at Hayspur Hatchery appears to include ancestry from inland RBT (2 N = 58; Omy25 and Omy29 are fused as a metacentric chromosomes) as well as the common hatchery RBT derived from coastal California (2 N = 60; Omy25 and Omy29 are acrocentric chromosomes) (R. F. Leary, Montana Fish, Wildlife and Parks, personal communication). Admixture between the two RBT stocks would generate Robertsonian metacentric (2 N = 58) and acrocentric (2 N = 60) polymorphs, as well as Robertsonian heterozygotes (2 N = 59). Subsequent hybridization between the polymorphic Kamloops stock and YCT would produce F_1_ hybrids that were Robertsonian heterozygotes (for example, Female 2 and Male 1) comprised of 28 linkage groups, containing the RBT metacentric fusion Omy25_Omy29 and the YCT acrocentric homologues to Omy25 and Omy29, as well as Robertsonian acrocentric homozygotes (for example, Female 1 and Male 2) comprised of 29 linkage groups, containing RBT acrocentric chromosomes Omy25 and Omy28 and their YCT homologues (Figure 2).

### Recombination suppression

Conclusions based on recombination differences between the hybrid and RBT maps have limitations, as rates can vary between related species [36], as well as among individuals within species [18,19,24]. Indeed, we found significant differences in pairwise recombination rates between the two RBT maps [21,33] used to construct the female consensus map across Omy5, Omy8, Omy14, Omy19, Omy20, and Omy22. Furthermore, differences in marker density among hybrid and RBT maps could also account for recombination differences by affecting map distance estimates. However, our objective was to perform a comparative analysis to identify recombination suppression broadly across the genome. Because recombination frequency appears correlated with chromosome arm number [37] and broad-scale recombination rates tend to be conserved between closely related species [36], we might expect similar recombination rates between RBT and YCT. Therefore, although absolute differences between map distances can be affected by number of markers and number of individuals mapped, the trend revealed by the comparative analyses yields interesting insight into recombination suppression in the hybrids, which could indicate the presence of chromosome rearrangements or genic incompatibilities.

Chromosome rearrangements can generate recombination suppression in heterokaryotypes (chromosomal hybrids) through unbalanced gametes which results in non-recombinants being the only viable gametes [38], or by restricting recombination between rearrangements [39]. Although unbalanced gametes cannot be ruled out, we consider them an unlikely cause of suppression in the F_1_ hybrids. First, conservation of synteny and marker order between the hybrid and RBT maps [21,33] suggests that YCT and RBT chromosomes are highly collinear, which would facilitate normal pairing between homologues and alternate disjunction in F_1_ hybrids. Second, because RBT and YCT contain the same number of chromosome arms, Robertsonian type rearrangements are considered to have played a significant role in generating the chromosome number differences between the species [34]. Meiotic pairing between homologues that differ by a Robertsonian rearrangement (i.e., pairing between a metacentric chromosome and the two acrocentric homologues) would produce a trivalent in the F_1_ hybrids, which would not necessarily cause malsegregation and unbalanced gametes. For example, the rate of nondisjunction was not different in individuals that were heterozygous for Robertsonian rearrangements compared to homozygotes in pink salmon (*O. gorbuscha*) [40], house mouse (*Mus musculus domesticus*) [41], and Eurasian common shrew (*Sorex araneus*) [42], suggesting this type of rearrangement produces balanced gametes.

Recombination was restricted in the F_1_ hybrids across several loci spanning chromosome fusion and fission differences between RBT and YCT; the Robertsonian fusion/fission within RYHyb25_29 and RYHyb20. This suggests that chromosome rearrangements did indeed suppress recombination. Rearrangements generate extensive linkage disequilibrium in heterokarotypic hybrids [3] and suppress recombination across genomic regions that extend beyond rearrangements [11,30,43]. Therefore, broad-scale recombination suppression across other linkage groups in the F_1_ hybrids could indicate the presence of rearrangements. We suggest that three other metacentric hybrid linkage groups, RYHyb11, RYHyb14, and RYHyb15, contained chromosomes that differed by arrangement between RBT and YCT. These linkage groups might represent Robertsonian rearrangements because recombination was suppressed across putative centromeres which could indicate that F_1_ hybrids were heterozygous for centric fusions/fissions. However, we found marker order differences within the suppressed regions in each of these three linkage groups compared to RBT linkage maps [21,33], which could possibly indicate inversions or translocations, or, alternatively, be due to reduced mapping power in regions with low recombination or the number of progeny used to construct the various maps.

Significant differences in recombination rates between several hybrid and RBT linkage groups could indicate the presence of inter-specific, genic incompatibilities. However, our broad-scale analysis hinders inference across smaller genomic scales where incompatibilities have been reported [44-46]. Nevertheless, we found numerous instances where two adjacent markers did not recombine in the female-merged hybrid map, but these same markers recombined in the consensus female RBT map. This might suggest that recombination between particular YCT and RBT genomic regions is maladaptive, or that the difference in adjacent recombination is an artifact of the number of offspring genotyped. Finer scale mapping across these regions that differ in recombination rate could be fruitful for identifying the presence of incompatibilities. Inter-specific incompatibilities may also be inferred from segregation distortion, as genotypes that occur less often than expected may be incompatible [3,14]. In addition, we expected that inter-specific incompatibilities would show consistent distortion among maps. For example, consistent allelic distortion within both RYHyb01 female hybrid maps and both RYHyb09 male hybrid maps could indicate the presence of incompatibilities. Although segregation distortion was present in several other linkage groups, a lack of consistency across hybrid maps suggests these distorted loci do not reflect genic incompatibilities between RBT and YCT.

### Recombination suppression within Robertsonian rearrangements

The recombination suppression pattern across the Robertsonian rearrangement RYHyb25_29 and the presumed Robertsonian rearrangement RYHyb15 differed from patterns reported between chromosome races in the house mouse [47,48] and common shrew [39]. Recombination in mice and shrews that were homozygous for Robertsonian fusions (i.e., homozygous for the metacentric polymorphism) appeared suppressed near centromeres and elevated toward telomeres in comparison to Robertsonian heterozygotes [39,47]. However, we observed the opposite; recombination was suppressed in Robertsonian heterozygotes (i.e., hybrids) across the putative centromere and elevated toward telomeres in comparison to fusion homozygotes (i.e., the female RBT consensus map). The mechanisms causing the difference between our results and the house mouse and common shrew are unclear. Given the approximate 3 million year divergence time between RBT and YCT [26] and the absence of historical secondary contact, genomic differences could have evolved near the centromeres of rearranged chromosomes that would restrict crossover events in Robertsonian heterozygotes, such as para- or pericentric inversions or genic incompatibilities. Alternatively, mechanistic processes governing meiotic crossover could differ among taxa. Determining how recombination suppression in the Omy25-Omy29 Robertsonian rearrangement differs between intra-specific (RBT chromosome races) and inter-specific hybrids could indicate the efficiency of this type of rearrangement as a barrier to gene flow in salmonids.

### Rearrangements protect genomic regions from recombination

Our results suggest that chromosome rearrangement is the main genomic obstacle for gene exchange between RBT and YCT. Rearrangements have been observed to reduce gene flow between several species pairs, including *Drosophila pseudoobscura* and *D. persimilis*[43], *Helianthus petiolaris* and *H. annuus*[10], and *S. araneus* and *S. antinorii*[49,50]. Rearrangements protect genomic regions from recombination, enabling genes within or closely linked to the rearranged genome to differentiate between heterokaryotypes while unrestricted gene flow occurs between regions with similar composition [45,51]. As a result, fitness related genes could accumulate within regions that are protected from recombination and diverge in the face of hybridization [45,51]. Indeed, genes involved with reproductive isolation have mapped to chromosome rearrangements [31,32]. The effectiveness of rearrangements as recombination suppressors may be dependent on how chromosomes are reorganized; rearrangements that change gene order (e.g., inversions or translocations) may be more effective in protecting the genome from being disrupted than rearrangements that do not change gene order (e.g., fusions or fissions). However, several studies indicate that Robertsonian type rearrangements restrict gene flow [11,49,50,52], but with extreme interbreeding this type of rearrangement may be an ineffective barrier [11].

Given the hybrid linkage map results, we predict that, within introgressed populations, inter-specific recombination will be restricted in particular genomic regions where chromosome arrangement differs between YCT and RBT. This prediction is supported by studies that have reported reduced gene flow across chromosome rearrangements within stable hybrid zones [10,11,50,52]. Because the majority of hybrid linkage groups appeared to have similar marker order and arrangement with RBT, few chromosomes would be expected to contain regions with restricted recombination, although suppression could potentially extend broadly across these chromosomes [11,30,43]. Within a long standing hybrid zone between house mouse chromosome races, linkage disequilibrium persisted among loci mapping near Robertsonian rearrangements [11], indicating that these types of rearrangements can generate and maintain linkage disequilibrium. Given that RBT-YCT introgression is recent and rearrangements produce extensive linkage disequilibrium [3], large genomic regions that flank centric fusion/fission differences could be expected to remain intact within some YCT chromosomes. As a consequence, recombination suppression between heterokaryotypes could protect genomic regions from being disrupted and enable co-adapted gene complexes and/or local adaptations linked to the rearrangements to persist within YCT admixed with RBT.

### Segregation distortion mechanisms

The low level of segregation distortion observed in the RBT-YCT hybrid maps was unexpected. Several studies have reported high levels of segregation distortion, greater than 15% of loci at *P* < 0.05, within hybrid maps across a variety of taxa, including interspecific crosses between *Mimulus guttatus* and *M. nastutus*[8], *Lepomis cyanellus* and *L. megalotis*[53], and *Nasonia giraulti* and *N. vitripennis*[54] and between intraspecific crosses of *Salvelinus alpinus*[55], *M. guttatus*[56], *Ceratodon purpureus*[57], *Coregonus clupeaformis*[58], and *Tigriopus californicus*[59]. Divergence time estimates between parental lineages used for several of these maps ranged from 0.1-0.2 MYA to 13–16 MYA [60-63]. We would expect comparable distortion levels to these studies following the estimated 3 million year divergence time between RBT and YCT. Our findings are probably not due to a lack of power for detecting distortion, because we mapped a similar number of markers per linkage group as the studies above, although the number of individuals per mapping panel was less than these studies, except Woram *et al.*[55]. Nevertheless, it is intriguing that segregation distortion was limited and few consistent distortion patterns emerged between sexes and families. The low level of distortion suggests that few incompatibilities exist between RBT and YCT, and may partially explain why these species readily form hybrid swarms.

Although segregation distortion was limited, we found a few noteworthy cases. A variety of mechanisms may cause distortion [14,64,65]. Understanding the causes of distortion typically requires in-depth study, but we discuss two mechanisms. First, pseudolinkage likely caused allelic distortion within two homeologous linkage groups in Male 2 (RYHyb07/RYHyb18 and RYHyb06/RYHyb27). Pseudolinkage may be implicated when homeologous linkage groups both show allelic segregation distortion [14], and we observed this distortion in YCT allele frequencies. Second, meiotic drive could have caused distortion. Meiosis is asymmetric in females and results in one functional haploid gamete per germ cell, compared to males where symmetric meiosis results in four functional gametes per germ cell [64]. Therefore, any process that results in non-random segregation of chromosomes during oogenesis may lead to distortion in females. Female meiotic drive is characterized by competition among centromeres for spindle fiber attachment during meiosis and oogenesis [64]. Because the centromere on one chromosome may outcompete the centromere on the homologue for deposition into the oocyte, allele frequencies in female maps may be distorted at loci mapping near centromeres [66]. Male meiotic drive is characterized by competition between alleles during sperm development and results in differential gamete success. Genomic divergence between species could escalate competition between meiotic drive elements, causing segregation distortion in their hybrid progeny [67]. Consistent distortion patterns within RYHyb01 between female hybrid maps and within RYHyb09 between male hybrid maps suggest that meiotic drive could be acting within the hybrids.

### Sex chromosomes

Conservation of synteny with the RBT sex chromosome, Omy1, suggests that RYHyb28 is the sex-linkage group. Indeed, sex has been established as mapping to homologs in YCT and RBT [68]. Conservation of sex-linkage groups is uncommon across several salmonid species [20,69-71]. Two alternative mechanisms have been proposed to account for the lack of conservation among sex-linkage groups in salmonids: either the sex-determining gene is the same among species but has moved to different chromosomes in different lineages, or sex determination has evolved independently in different lineages [69]. Regardless of the mechanism, the lack of homology among sex chromosomes could be an important factor for restricting inter-specific hybridization, and could explain why hybridization is not commonly observed between many sympatric salmonid species and, conversely, why two species pairs identified as having homologous sex-linkage regions, YCT and RBT [68] and Arctic charr (*S. alpinus*) and brook charr (*S. fontinalis*) [72], introgress in the wild [73,74]. The relationship between conservation of sex chromosomes and introgression is confounded in brown trout (*Salmo trutta*) and Atlantic salmon (*S. salar*) because their sex chromosomes lack homology [70], yet the species hybridize in the wild [75]. However, brown trout and Atlantic salmon hybrids suffer reduced viability [76] and introgression is rare [75]. Nevertheless, homology between sex-linkage groups could possibly facilitate introgression between RBT and YCT.

## Conclusions

Our results are consistent with a growing number of studies demonstrating that chromosome rearrangements reduce gene flow by suppressing recombination [10,11,30,43,49,50,52]. Although much of the RBT and YCT genome appears porous to gene exchange, our study indicates that chromosome rearrangements between RBT and YCT act as genomic obstacles to introgression. As a consequence, chromosome arrangements could have a significant influence on the evolution of YCT-RBT hybrid genomes. For example, within admixed populations, rearrangements could protect particular YCT genomic regions from RBT introgression, enabling large segments of the YCT genome to remain intact. If fitness related genes are linked to these rearrangements, recombination suppression could preserve them from being disrupted. This could provide an adaptive advantage to hybrids that contain these fitness related genes and enable these genes to persist within admixed populations. In contrast, unobstructed gene flow between chromosomes with similar arrangement would disrupt linkage associations within each species and create new genetic variation for selection to act upon.

The genetic linkage map established herein for YCT-RBT F_1_ hybrids provides an initial framework for investigating hybridization and is likely relevant to other cutthroat trout subspecies that introgress with RBT, and, therefore, may serve as a general model of genomic introgression. In addition, our study defines a set of genome-wide species markers that can be applied to conservation and management of indigenous YCT.

## Methods

### Mapping families

Hybrid F_1_ YCT-RBT parents were generated by crossing female YCT collected from Henry’s Lake Fish Hatchery and Fish Management Station, Idaho Department of Fish and Game (IDFG), with male RBT (Kamloops stock) from Hayspur Hatchery (IDFG). Mature F_1_ hybrids were collected at Henry’s Lake in March of 2004 and used to generate two F_2_ hybrid full-sib crosses (Family 1, N = 54 mapping progeny; Family 2, N = 53 mapping progeny). Because a YCT genetic map does not exist, we also constructed a YCT cross (N = 48 mapping progeny) so that we could clarify linkage anomalies observed between the F_1_ hybrid and published RBT maps [21,33]. Fin tissues were sampled from the parents and we confirmed the hybrid/species status of F_1_ hybrid and YCT parents by screening 12 species-specific markers that differentiate RBT and YCT (Additional file 1, Worksheet 1). Crosses were reared 10 months post-fertilization at which point the fish were euthanized and fin tissues sampled. DNA was extracted using DNeasy kits (Qiagen Inc., Valencia, CA, USA).

### Mapping markers

Rainbow trout genetic linkage maps developed by Guyomard *et al.*[33] and Rexroad *et al.*[21] served as templates for F_1_ hybrid map construction. We applied 294 microsatellite primers (Additional file 1, Worksheet 1), spanning a large portion of each RBT linkage group and ensuring coverage across the centromere for metacentric chromosomes. Microsatellite amplification and PCR product visualization followed the methods of McClelland and Naish [20].

We also applied 169 SNPs (Additional file 1, Worksheet 1). SNPs were interrogated using TaqMan 5’ nuclease assays (Applied Biosystems Inc., Carlsbad, CA, USA) or SNPtype assays (Fluidigm Corporation, San Francisco, CA, USA). All genotyping was carried out in 96.96 Dynamic Genotyping Arrays on an EP1 Genotyping System (Fluidigm Corporation), with a pre-amplification step, following manufacturer’s protocols. Genotypes were determined using the Fluidigm SNP Genotyping Analysis software (v 3.0.2), with confidence threshold set to 80%.

In addition, 14 insertion/deletion and four RFLP species-diagnostic primers (Additional file 1, Worksheet 1) were included in the map. Amplifications were performed in 20 μl reaction volumes consisting of 15 ng genomic DNA, 1X NH_4_ Reaction Buffer (Bioline, Taunton, MA, USA), 1.5-2.5 mM MgCl_2_, 200 μM each dNTP, 1.5 pmol of each primer, and 0.5 units *Taq* polymerase (Bioline, Taunton, MA, USA). PCR products were visualized on 2-4% agarose gels stained in ethidium bromide.

### Linkage analysis

Linkage maps were established using LINKMFEX v2.3 software package [14] with an LOD threshold at 3.0. Because male salmonids show less recombination across the genome than females [24], we initially constructed parent-specific linkage maps. Hereafter, parent-specific linkage maps are referred to as Female 1 (Family 1 female parent), Male 1 (Family 1 male parent), Female 2 (Family 2 female parent), and Male 2 (Family 2 male parent). Salmonids have high crossover interference and typically have one or no crossovers per chromosome arm [77]; we therefore used the pairwise recombination fraction, theta (*Θ*), between adjacent markers to estimate map distances. Male and female maps were compared by generating sex-specific and sex-merged maps using LINKMFEX. The total number of linkage groups in the F_1_ hybrid map was determined from sex-merged maps. Linkage maps were graphically represented using the program MAPCHART [78]. Markers that were heterozygous for the same alleles in both parents were excluded from the map because these parental genotypes reduce the number of informative progeny, generate missing data, and reduce mapping power for these markers. All species-diagnostic SNPs, indels, and RFLPs were heterozygous in both of the F_1_ hybrid parents, and thus these markers were not ordered within the map. However, we did assign each of these markers to a specific linkage group.

### Recombination rate analyses

We estimated the average recombination ratio across the genome and across each linkage group between parents within each family and between parents of the same sex using LINKMFEX. Significant differences in genome-wide and linkage group-wide recombination rates were identified by summing G-test values and degrees of freedom across each comparison.

To determine if introgression suppressed recombination rates, we generated a consensus female RBT map from Guyomard *et al.*[33] and Rexroad *et al.*[21] using recombination distances between markers and compared recombination rates to the female-merged F_1_ map. The consensus female RBT map was generated using only markers in common with the female-merged F_1_ hybrid map (Additional file 1, Worksheets 10). We ensured that maps had the same marker order among RBT and female-merged F_1_ hybrids by removing markers that differed in their rank in the order. Markers used to compare recombination rates generally covered a substantial proportion of each RBT linkage group and were not restricted to areas of low recombination (centromeric regions) or areas of high recombination (telomeric regions). Kosambi map distances from Rexroad *et al.*[21] were converted to *Θ* map distances by applying the formula *θ* = 0.5(*e*^4*k*^ − 1)/(*e*^4*k*^ + 1) [79], where k = Kosambi distance. Prior to generating the consensus female RBT map, we tested for significant differences in recombination distances between adjacent loci between the two female RBT maps for each linkage group. The two female RBT maps were merged into the consensus map using LINKMFEX. We then tested for significant differences in recombination rate between the female-merged F_1_ map and the consensus female RBT map across linkage groups against the null hypothesis of no difference in recombination rate. Significant differences in the recombination rate were identified as described above.

### Segregation distortion analyses

We tested for allelic distortion using a sliding window analysis, because loci exhibiting segregation distortion often cluster together within the genome [8]. We established a 25 centiMorgan (cM) interval for the sliding window, because linkage was not supported for loci greater than 25 cM in distance at the LOD = 3.0 threshold. Species-specific markers were used to specify the most likely F_1_ parent chromosome phases as either YCT or RBT using LINKMFEX. Locus-specific allele frequencies were tested for deviation from 1:1 Mendelian expectations by summing G-test values and degrees of freedom within each 25 cM interval.

For testing genotypic segregation distortion, we used the most likely allele phases obtained from LINKMFEX in the sliding window analysis to assign locus-specific genotypes in the F_2_ hybrid progeny as YCT homozygote (YCT/YCT), RBT homozygote (RBT/RBT), or heterozygote (YCT/RBT). Significant deviation from the expected 1:2:1 Mendelian genotypic proportions was determined for each locus by applying chi square tests followed by multiple comparison corrections using a false discovery rate (B-Y FDR) [80] across all loci.

## Competing interests

The authors declare that they have no competing interests.

## Authors’ contributions

COO performed genotyping of microsatellite and indel markers, carried out the statistical analyses, and drafted the manuscript. VLP and JCG performed SNP genotyping. LH and KN helped interpret the data and draft the manuscript. COO, LH, and KN conceptualized the study. All authors read and approved the final manuscript.

## Authors’ information

C. O. Ostberg: U.S. Geological Survey, Western Fisheries Research Center, 6505 NE 65th Street, Seattle, WA 98115 and School of Aquatic and Fishery Sciences, University of Washington, 1122 NE Boat Street, Box 355020, Seattle, WA 98105, USA, costberg@usgs.gov L. Hauser: School of Aquatic and Fishery Sciences, University of Washington, 1122 NE Boat Street, Box 355020, Seattle, WA 98105, USA, lhauser@uw.edu V. L. Pritchard: Department of Biological Sciences, University of Turku, 20014 Turku, Finland, victorialpritchard@gmail.com J. C. Garza: Southwest Fisheries Science Center, National Marine Fisheries Service and University of California, Santa Cruz, 110 Shaffer Road, Santa Cruz, CA 95060, USA, carlos.garza@noaa.gov K. A. Naish: School of Aquatic and Fishery Sciences, University of Washington, 1122 NE Boat Street, Box 355020, Seattle, WA 98105, USA, knaish@uw.edu.

## Supplementary Material

Additional file 1 **This file contains numbered worksheets that provide information on mapping loci and linkage maps in Excel file format.** The worksheets includes mapping loci and references (worksheet 1), parent-specific linkage maps (worksheets 2–5), female- and male-merged linkage maps (worksheets 6 and 7), sex-merged linkage map (worksheet 8), species diagnostic markers localized to hybrid linkage groups (worksheet 9), and F_1_ and RBT consensus maps used to investigate recombination suppression. Click here for file

Additional file 2 **This PDF file includes figures representing parent-specific F**_**1**_** hybrid linkage maps and inferred Yellowstone cutthroat trout (YCT) allele frequencies for each locus.** Click here for file

Additional file 3 **This PDF file includes figures representing female-, male-, and sex-merged F**_**1 **_**hybrid linkage maps.** Click here for file

Additional file 4 **This PDF file includes figures that compare map distances, in centiMorgans, across the same markers in the female-merged F**_**1**_** hybrid linkage map (X-axis) and the female consensus rainbow trout map (Y-axis) for each linkage group.** Click here for file

Additional file 5 This PDF file includes figures representing inferred genotypic frequency distributions for Yellowstone cutthroat trout homozygotes, rainbow trout homozygotes, and heterozygotes at each locus in Family 1, Family 2, and loci scored in both families combined across each hybrid linkage group. Click here for file
