# Supplementary material for: Chromosome rearrangements, recombination suppression, and limited segregation distortion in hybrids between Yellowstone cutthroat trout (Oncorhynchus clarkii bouvieri) and rainbow trout (O. mykiss)
Source: BMC Genomics. 2013 Aug 22;14:570. doi: 10.1186/1471-2164-14-570 (PMC3765842; doi:10.1186/1471-2164-14-570)
Supplement: Additional file 2 — This PDF file includes figures representing parent-specific F1 hybrid linkage maps and inferred Yellowstone cutthroat trout (YCT) allele frequencies for each locus. [file 1471-2164-14-570-S2.pdf]

Additional file 2. Parent-specific F_1 hybrid linkage maps in *p* to *q* orientation and Yellowstone cutthroat trout (YCT) allele frequencies. Map distances (cM) represent the recombination fraction (theta) between loci. A 25 cM sliding window was used to identify blocks of loci containing YCT allele frequencies that deviated significantly from expected frequencies. Yellowstone cutthroat trout allele frequencies are plotted for each locus mapped within linkage groups. * indicates $P < 0.05$ and ** indicates $P < 0.01$.

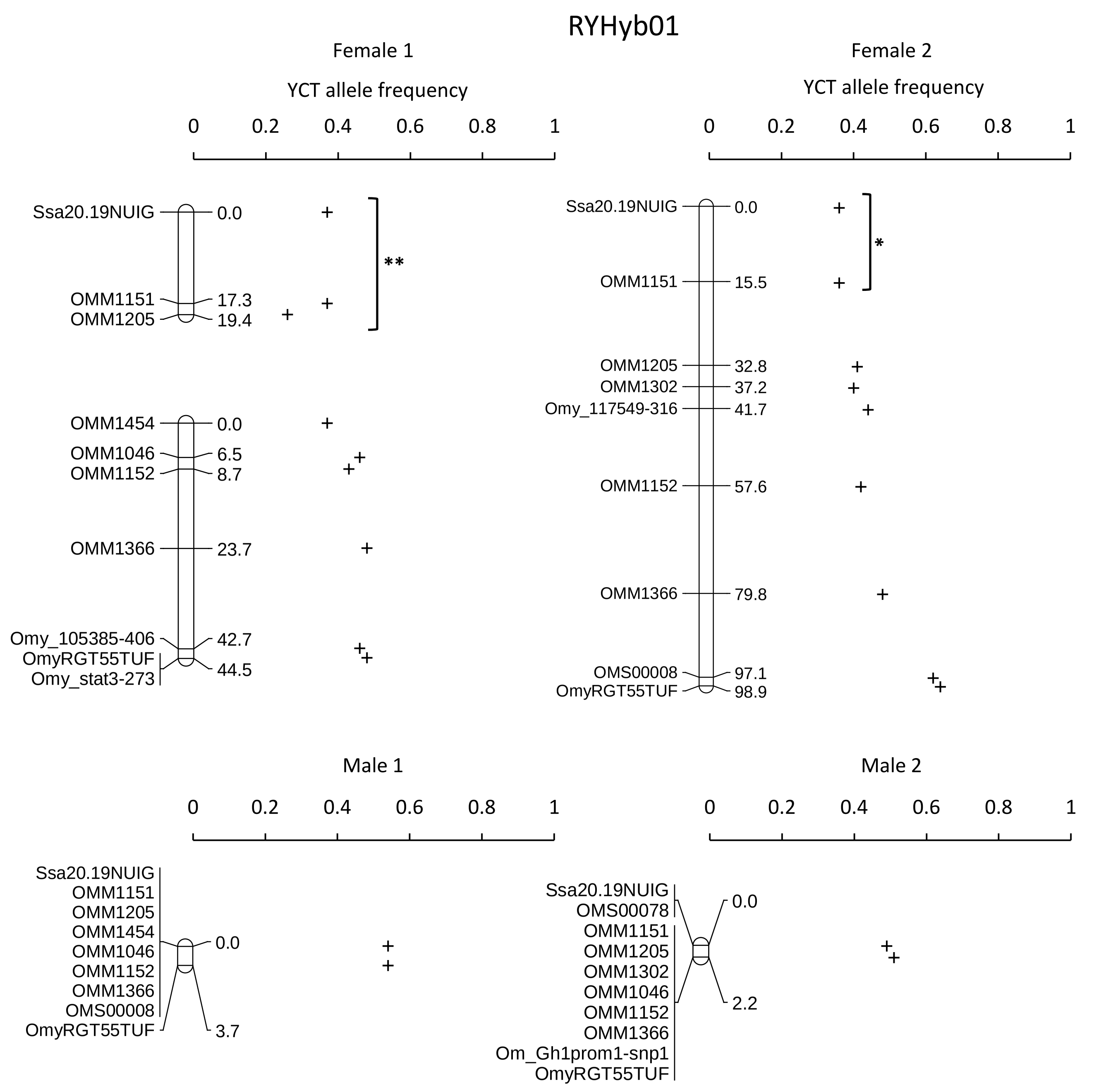
RYHyb01
Female 1
YCT allele frequency
0
0.2
0.4
0.6
0.8
1
Ssa20.19NUIG 0.0
OMM1151 17.3
OMM1205 19.4
**
OMM1454 0.0
OMM1046 6.5
OMM1152 8.7
OMM1366 23.7
Omy_105385-406 42.7
OmyRGT55TUF
Omy_stat3-273 44.5
Female 2
YCT allele frequency
0
0.2
0.4
0.6
0.8
1
Ssa20.19NUIG 0.0
OMM1151 15.5
*
OMM1205 32.8
OMM1302 37.2
Omy_117549-316 41.7
OMM1152 57.6
OMM1366 79.8
OMS00008 97.1
OmyRGT55TUF 98.9
Male 1
0
0.2
0.4
0.6
0.8
1
Ssa20.19NUIG
OMM1151
OMM1205
OMM1454
OMM1046
OMM1152
OMM1366
OMS00008
0.0
OmyRGT55TUF 3.7
Male 2
0
0.2
0.4
0.6
0.8
1
Ssa20.19NUIG
OMS00078
0.0
OMM1151
OMM1205
OMM1302
OMM1046
2.2
OMM1152
OMM1366
Om_Gh1prom1-snp1
OmyRGT55TUF

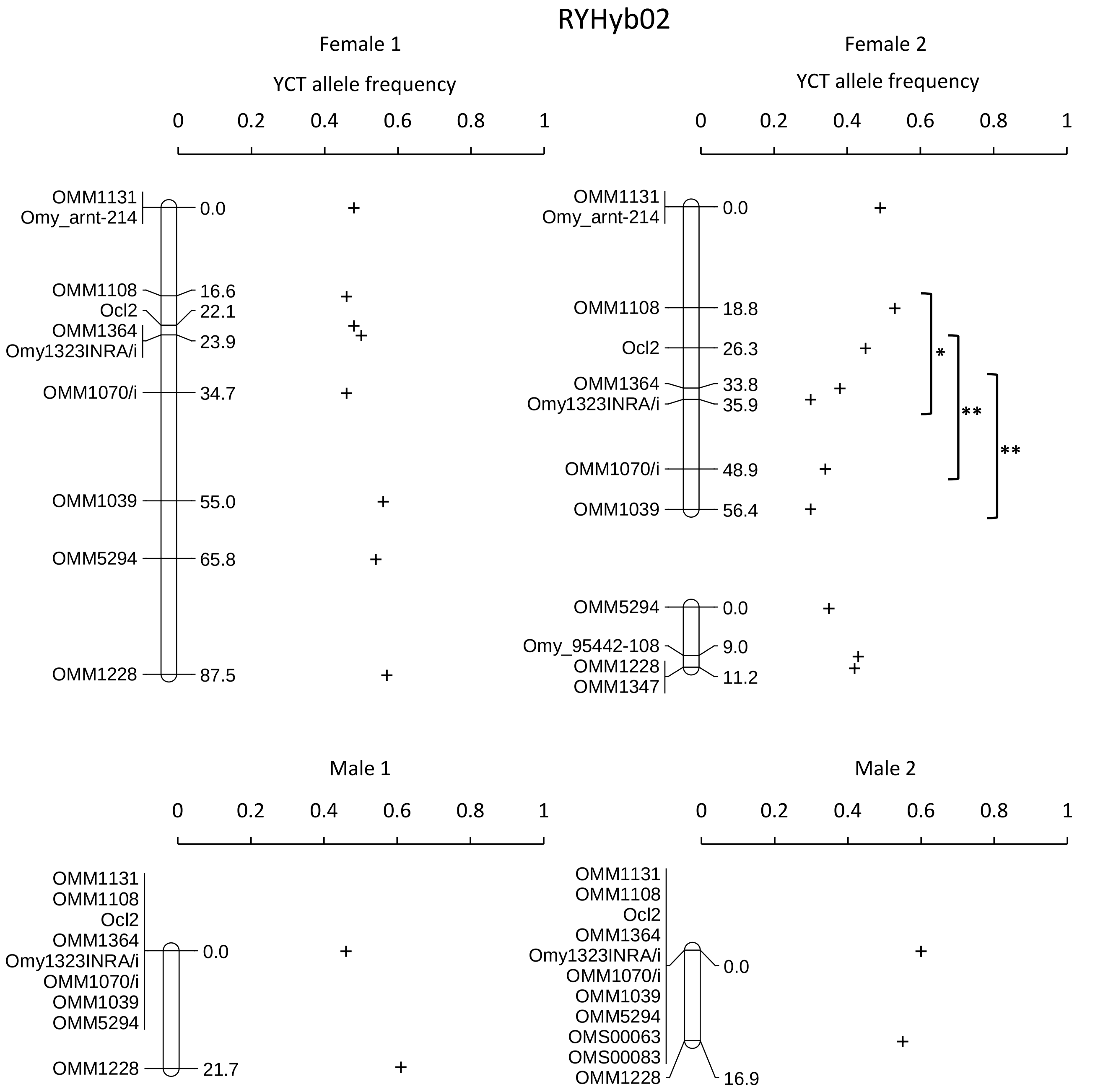

RYHyb02
Female 1
YCT allele frequency
0
0.2
0.4
0.6
0.8
1
OMM1131
Omy_arnt-214
0.0
OMM1108
16.6
Ocl2
22.1
OMM1364
Omy1323INRA/i
23.9
OMM1070/i
34.7
OMM1039
55.0
OMM5294
65.8
OMM1228
87.5
Female 2
YCT allele frequency
OMM1108
18.8
Ocl2
26.3
OMM1364
33.8
Omy1323INRA/i
35.9
OMM1070/i
48.9
OMM1039
56.4
*
**
**
OMM5294
0.0
Omy_95442-108
9.0
OMM1228
OMM1347
11.2
Male 1
OMM1070/i
OMM1039
OMM5294
21.7
Male 2
OMS00063
OMS00083
16.9

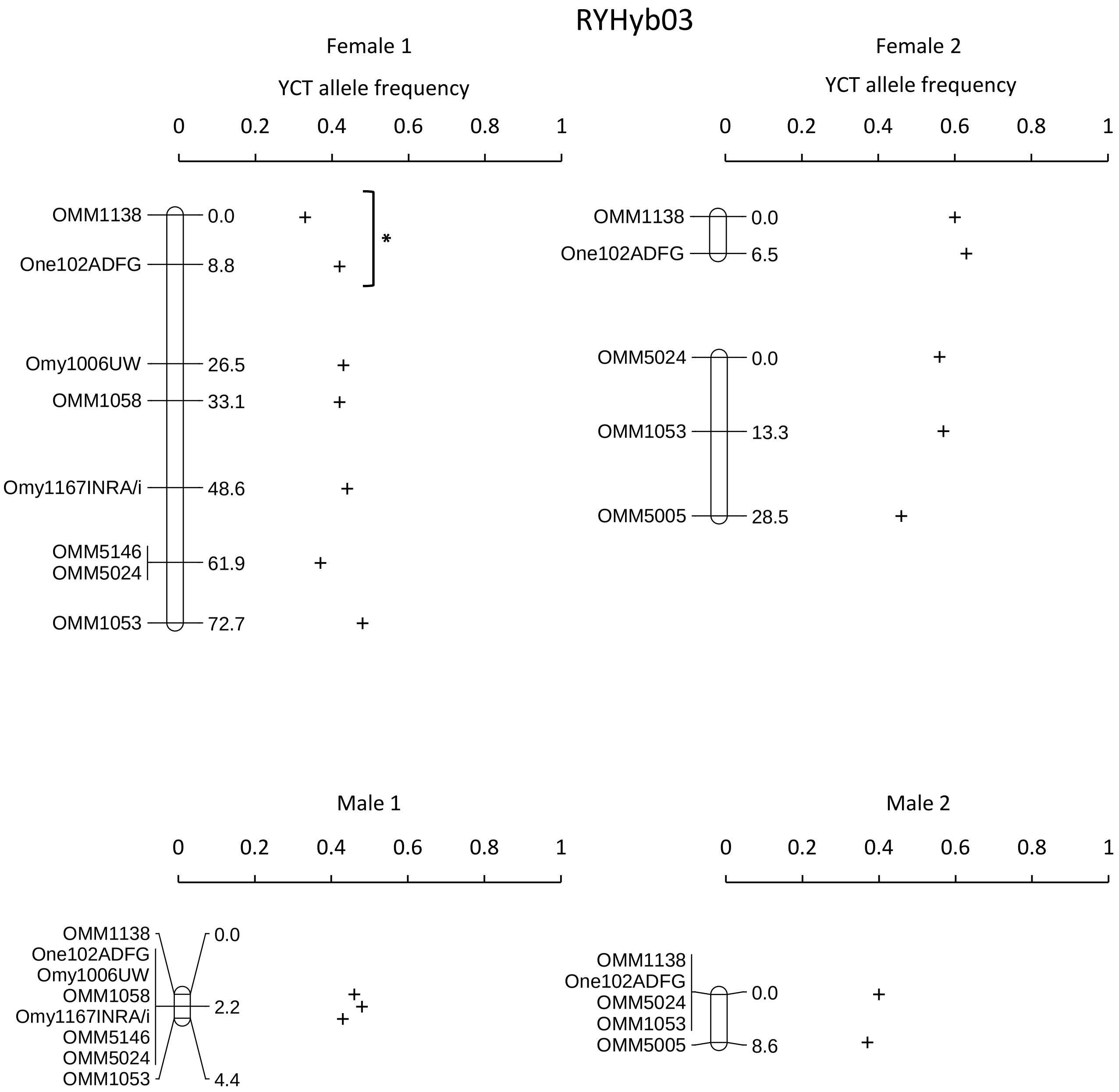

RYHyb03
Female 1
YCT allele frequency
0
0.2
0.4
0.6
0.8
1
OMM1138
One102ADFG
Omy1006UW
OMM1058
Omy1167INRA/i
OMM5146
OMM5024
OMM1053
0.0
8.8
26.5
33.1
48.6
61.9
72.7
*
Female 2
YCT allele frequency
OMM1138
One102ADFG
0.0
6.5
OMM5024
OMM1053
OMM5005
0.0
13.3
28.5
Male 1
OMM1138
One102ADFG
Omy1006UW
OMM1058
Omy1167INRA/i
OMM5146
OMM5024
OMM1053
0.0
2.2
4.4
Male 2
OMM1138
One102ADFG
OMM5024
OMM1053
OMM5005
0.0
8.6

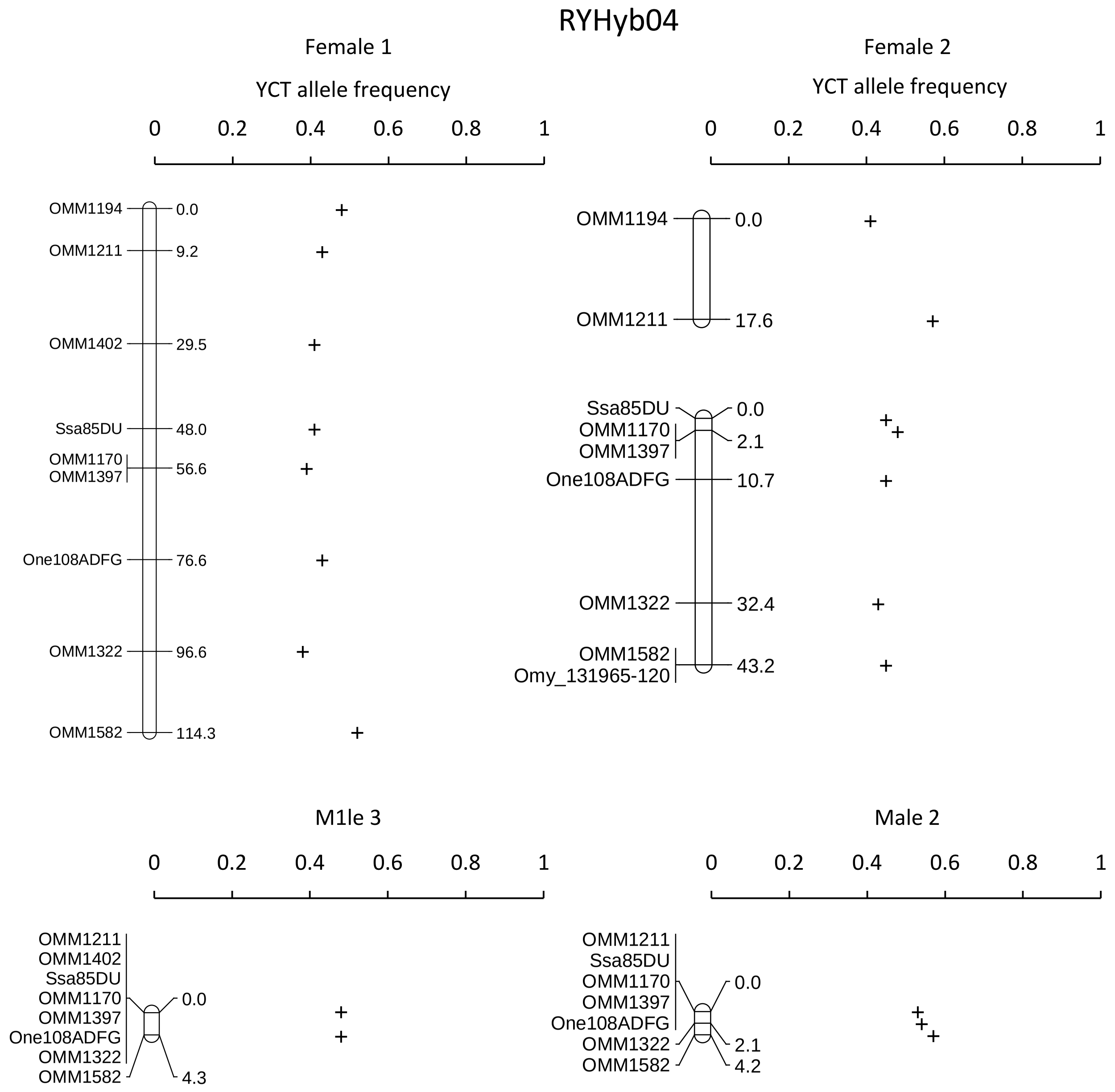
RYHyb04
Female 1
YCT allele frequency
0
0.2
0.4
0.6
0.8
1
OMM1194
0.0
OMM1211
9.2
OMM1402
29.5
Ssa85DU
48.0
OMM1170
OMM1397
56.6
One108ADFG
76.6
OMM1322
96.6
OMM1582
114.3
Female 2
YCT allele frequency
0
0.2
0.4
0.6
0.8
1
OMM1194
0.0
OMM1211
17.6
Ssa85DU
0.0
OMM1170
OMM1397
2.1
One108ADFG
10.7
OMM1322
32.4
OMM1582
Omy_131965-120
43.2
M1le 3
0
0.2
0.4
0.6
0.8
1
OMM1211
OMM1402
Ssa85DU
OMM1170
OMM1397
One108ADFG
OMM1322
OMM1582
0.0
4.3
Male 2
0
0.2
0.4
0.6
0.8
1
OMM1211
Ssa85DU
OMM1170
OMM1397
One108ADFG
OMM1322
OMM1582
0.0
2.1
4.2

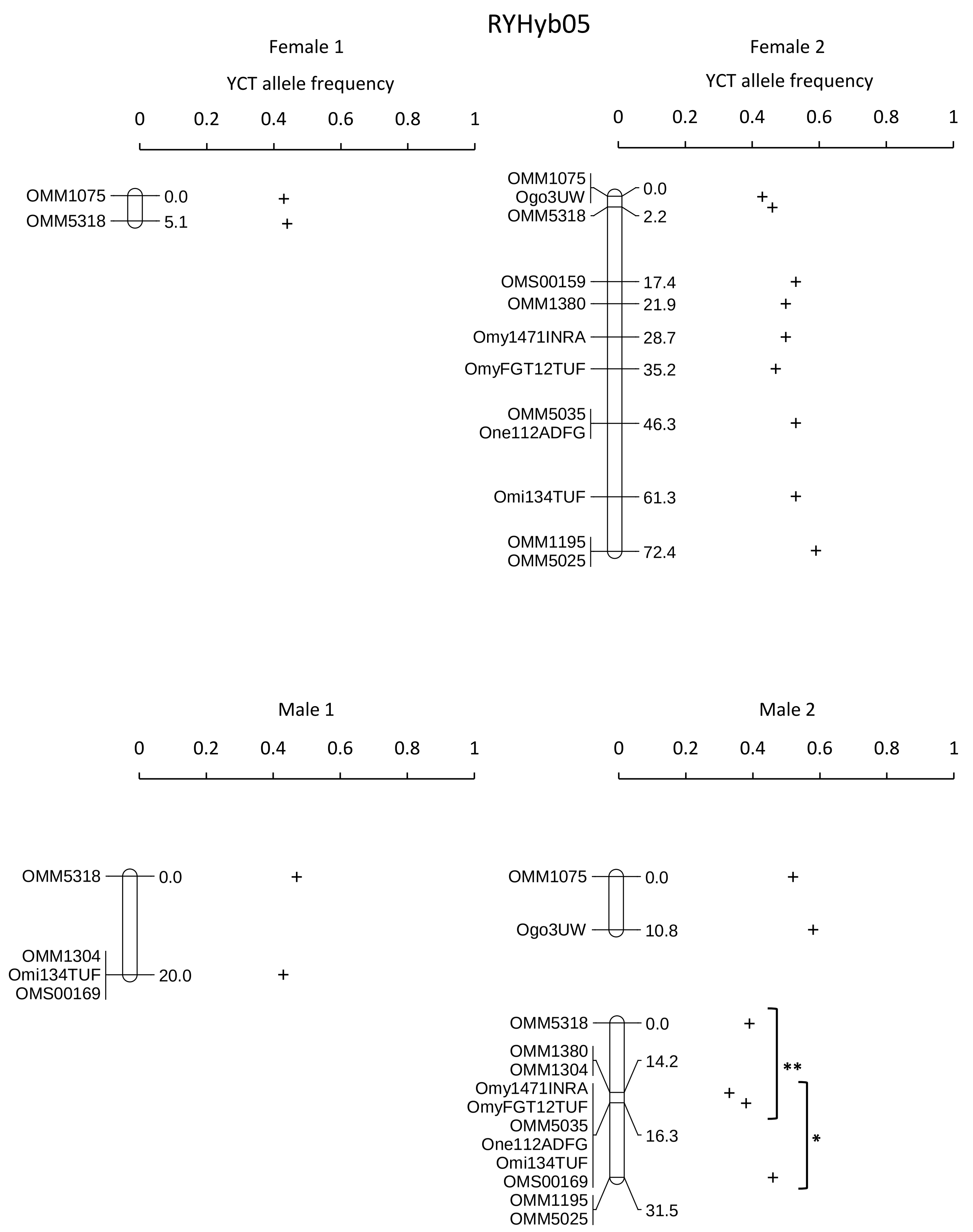
RYHyb05
Female 1
YCT allele frequency
0 0.2 0.4 0.6 0.8 1
OMM1075 0.0
OMM5318 5.1
Female 2
YCT allele frequency
0 0.2 0.4 0.6 0.8 1
OMM1075
Ogo3UW 0.0
OMM5318 2.2
OMS00159 17.4
OMM1380 21.9
Omy1471INRA 28.7
OmyFGT12TUF 35.2
OMM5035
One112ADFG 46.3
Omi134TUF 61.3
OMM1195
OMM5025 72.4
Male 1
0 0.2 0.4 0.6 0.8 1
OMM5318 0.0
OMM1304
Omi134TUF 20.0
OMS00169
Male 2
0 0.2 0.4 0.6 0.8 1
OMM1075 0.0
Ogo3UW 10.8
OMM5318 0.0
OMM1380
OMM1304 14.2
Omy1471INRA
OmyFGT12TUF
OMM5035 16.3
One112ADFG
Omi134TUF
OMS00169
OMM1195
OMM5025 31.5
**
*

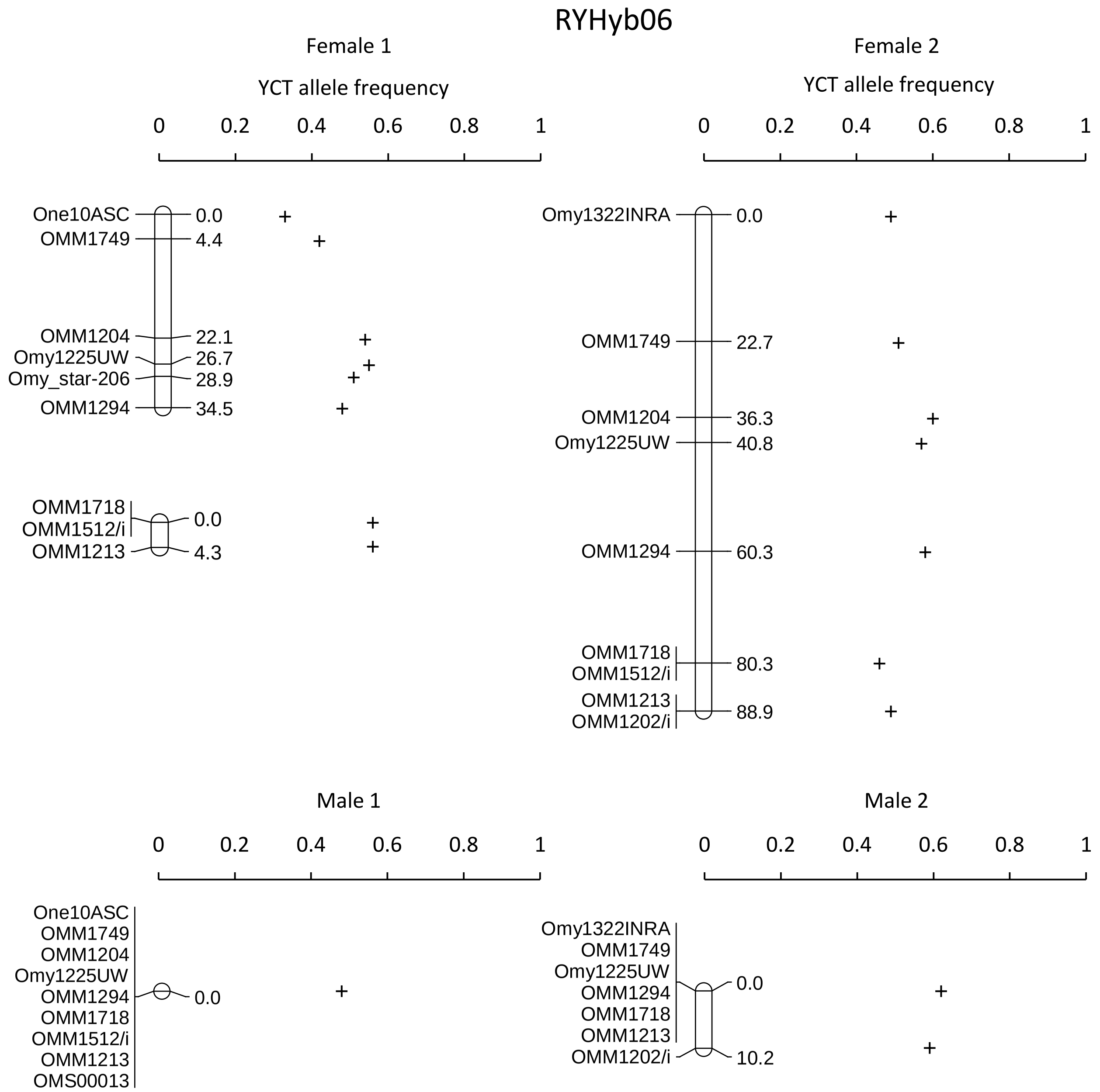

RYHyb06
Female 1
YCT allele frequency
0
0.2
0.4
0.6
0.8
1
One10ASC
OMM1749
OMM1204
Omy1225UW
Omy_star-206
OMM1294
0.0
4.4
22.1
26.7
28.9
34.5
OMM1718
OMM1512/i
OMM1213
0.0
4.3
Female 2
YCT allele frequency
Omy1322INRA
OMM1749
OMM1204
Omy1225UW
OMM1294
OMM1718
OMM1512/i
OMM1213
OMM1202/i
0.0
22.7
36.3
40.8
60.3
80.3
88.9
Male 1
One10ASC
OMM1749
OMM1204
Omy1225UW
OMM1294
OMM1718
OMM1512/i
OMM1213
OMS00013
0.0
Male 2
Omy1322INRA
OMM1749
Omy1225UW
OMM1294
OMM1718
OMM1213
OMM1202/i
0.0
10.2

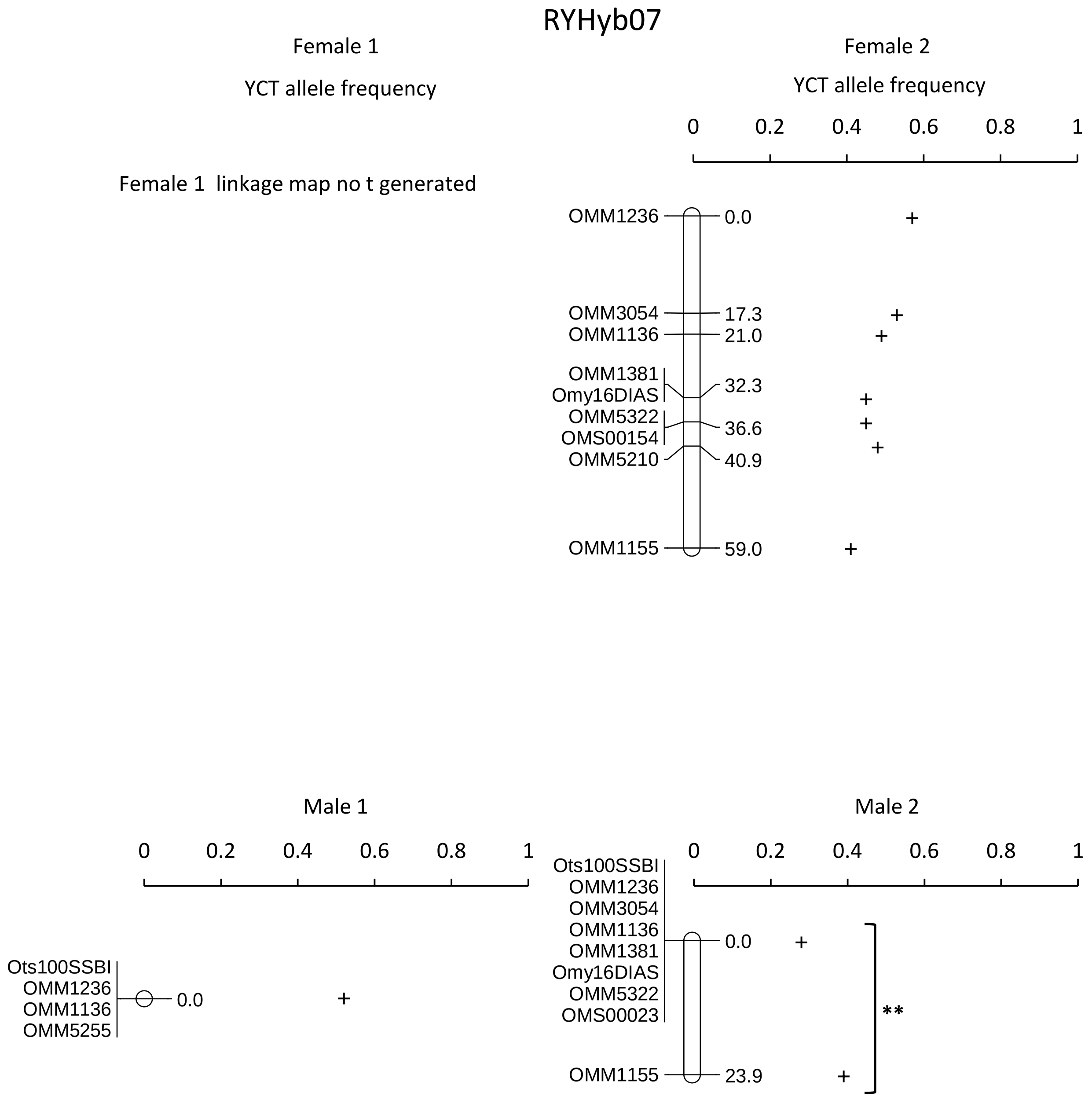

RYHyb07
Female 1
YCT allele frequency
Female 1 linkage map no t generated
Female 2
YCT allele frequency
0
0.2
0.4
0.6
0.8
1
OMM1236
0.0
OMM3054
17.3
OMM1136
21.0
OMM1381
Omy16DIAS
32.3
OMM5322
OMS00154
36.6
OMM5210
40.9
OMM1155
59.0
Male 1
Ots100SSBI
OMM1236
OMM1136
OMM5255
0.0
Male 2
Ots100SSBI
OMM1236
OMM3054
OMM1136
OMM1381
Omy16DIAS
OMM5322
OMS00023
0.0
OMM1155
23.9
**

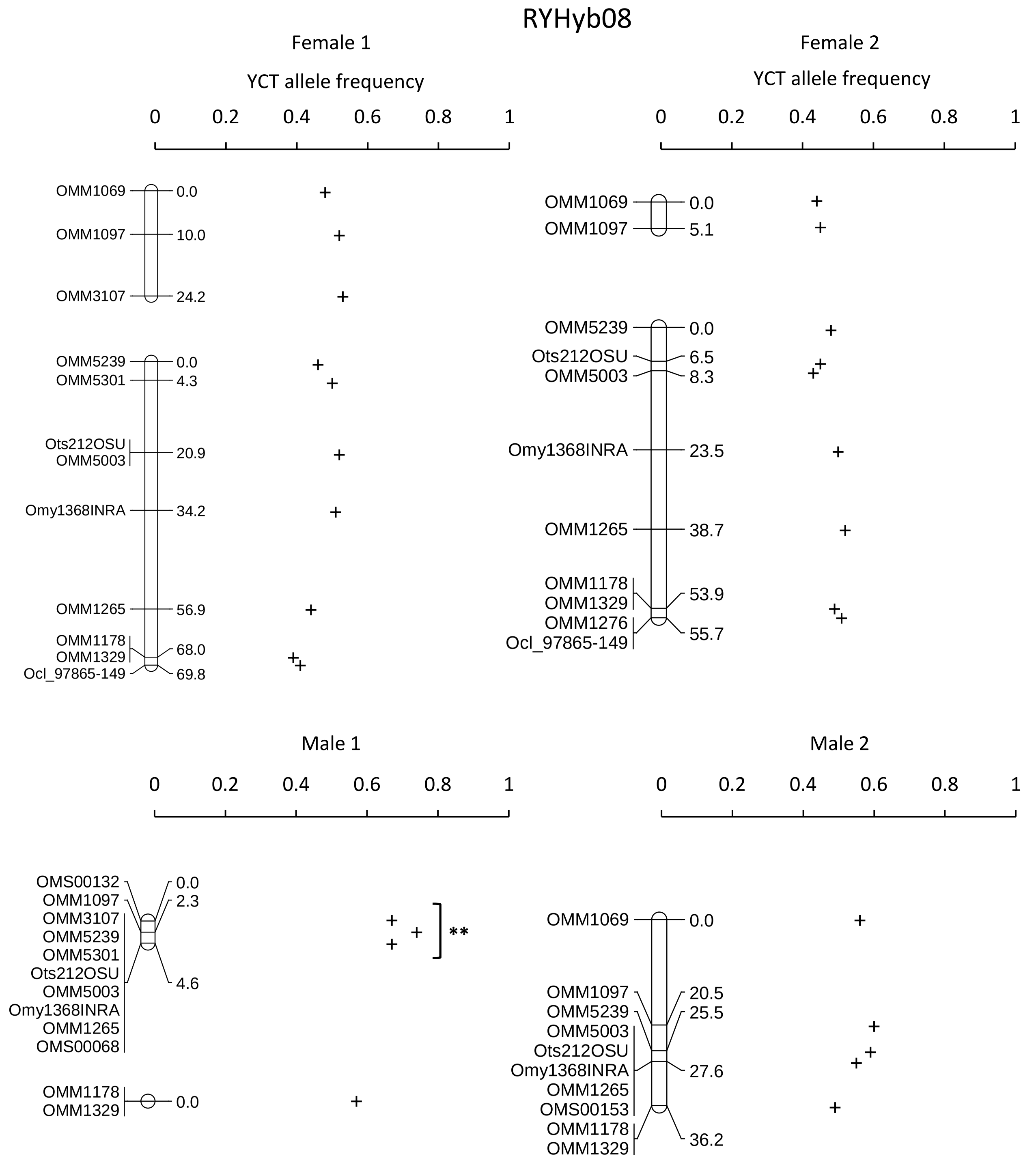
RYHyb08
Female 1
YCT allele frequency
0
0.2
0.4
0.6
0.8
1
OMM1069
0.0
OMM1097
10.0
OMM3107
24.2
OMM5239
0.0
OMM5301
4.3
Ots212OSU
OMM5003
20.9
Omy1368INRA
34.2
OMM1265
56.9
OMM1178
OMM1329
68.0
Ocl_97865-149
69.8
Female 2
YCT allele frequency
0
0.2
0.4
0.6
0.8
1
OMM1069
0.0
OMM1097
5.1
OMM5239
0.0
Ots212OSU
6.5
OMM5003
8.3
Omy1368INRA
23.5
OMM1265
38.7
OMM1178
OMM1329
53.9
OMM1276
Ocl_97865-149
55.7
Male 1
0
0.2
0.4
0.6
0.8
1
OMS00132
0.0
OMM1097
2.3
OMM3107
OMM5239
OMM5301
Ots212OSU
4.6
OMM5003
Omy1368INRA
OMM1265
OMS00068
**
OMM1178
OMM1329
0.0
Male 2
0
0.2
0.4
0.6
0.8
1
OMM1069
0.0
OMM1097
20.5
OMM5239
25.5
OMM5003
Ots212OSU
Omy1368INRA
27.6
OMM1265
OMS00153
OMM1178
OMM1329
36.2

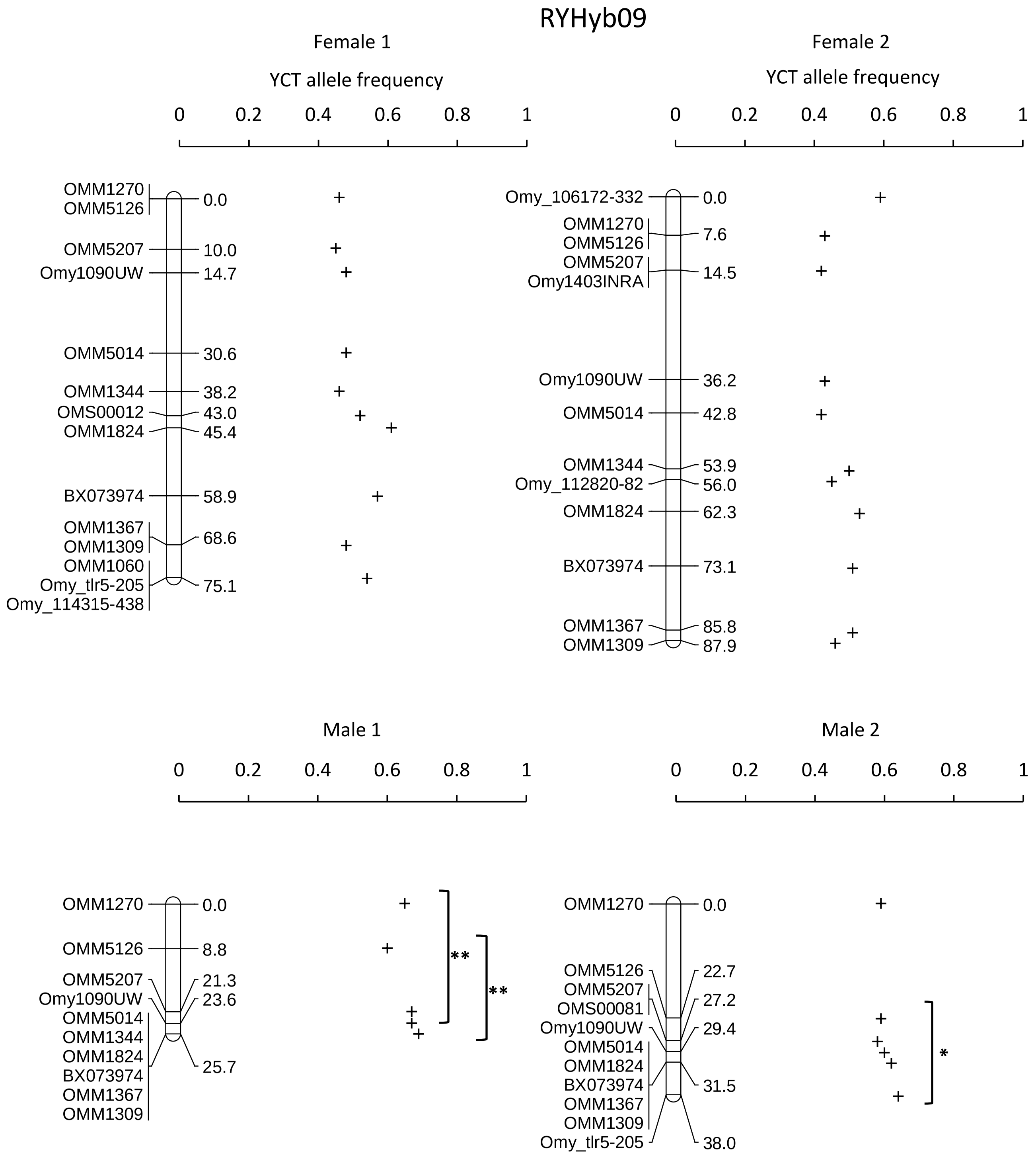
RYHyb09
Female 1
YCT allele frequency
0
0.2
0.4
0.6
0.8
1
OMM1270
OMM5126
0.0
OMM5207
10.0
Omy1090UW
14.7
OMM5014
30.6
OMM1344
38.2
OMS00012
43.0
OMM1824
45.4
BX073974
58.9
OMM1367
OMM1309
68.6
OMM1060
Omy_tlr5-205
Omy_114315-438
75.1
Female 2
YCT allele frequency
Omy_106172-332
0.0
OMM1270
OMM5126
7.6
OMM5207
Omy1403INRA
14.5
Omy1090UW
36.2
OMM5014
42.8
OMM1344
53.9
Omy_112820-82
56.0
OMM1824
62.3
BX073974
73.1
OMM1367
85.8
OMM1309
87.9
Male 1
OMM1270
0.0
OMM5126
8.8
OMM5207
21.3
Omy1090UW
23.6
OMM5014
OMM1344
OMM1824
BX073974
OMM1367
OMM1309
25.7
**
**
Male 2
OMM1270
0.0
OMM5126
22.7
OMM5207
27.2
OMS00081
Omy1090UW
29.4
OMM5014
OMM1824
BX073974
31.5
OMM1367
OMM1309
Omy_tlr5-205
38.0
*

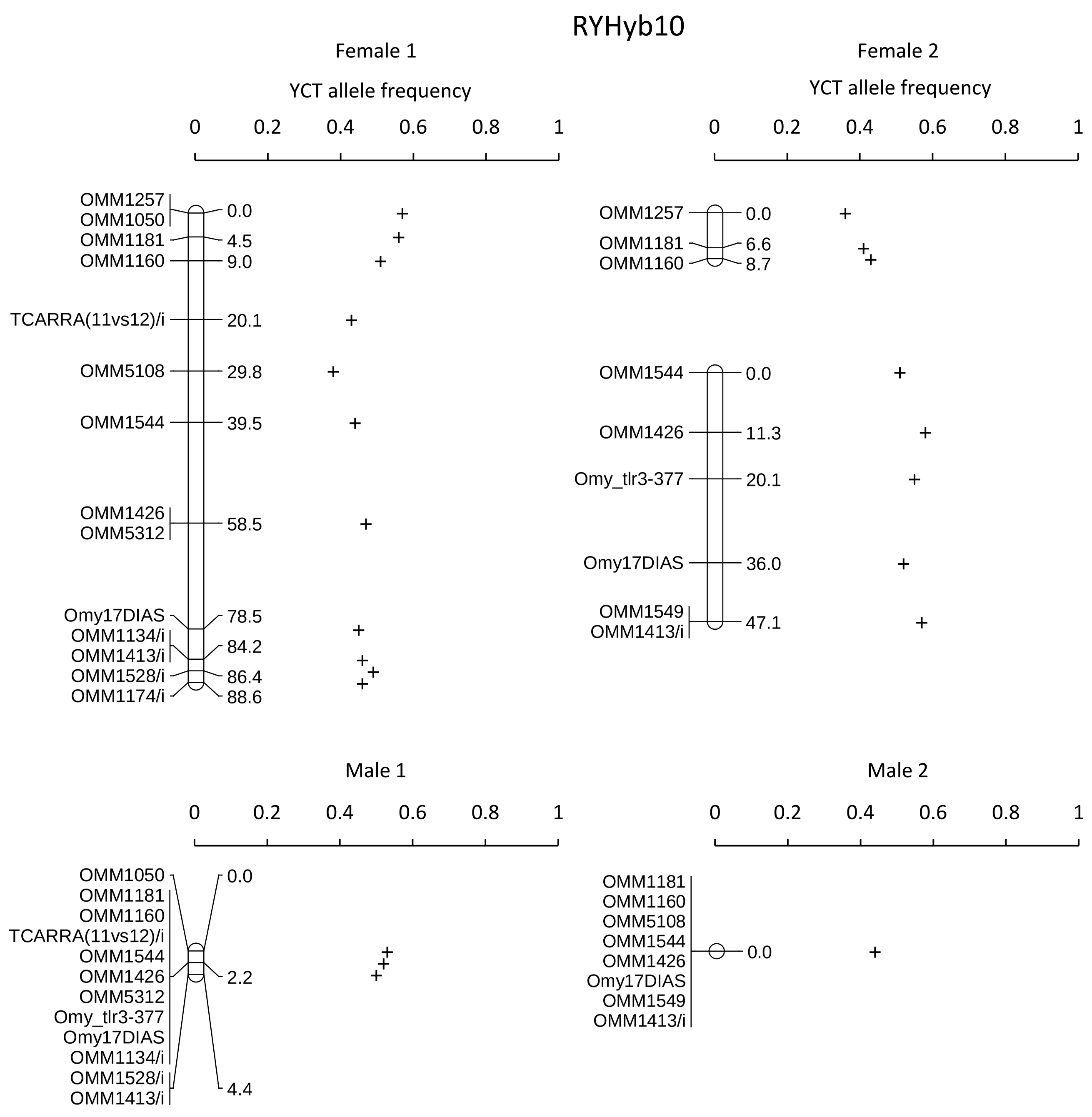
RYHyb10
Female 1
YCT allele frequency
0
0.2
0.4
0.6
0.8
1
OMM1257
OMM1050
0.0
OMM1181
4.5
OMM1160
9.0
TCARRA(11vs12)/i
20.1
OMM5108
29.8
OMM1544
39.5
OMM1426
OMM5312
58.5
Omy17DIAS
78.5
OMM1134/i
OMM1413/i
84.2
OMM1528/i
86.4
OMM1174/i
88.6
Female 2
YCT allele frequency
OMM1257
0.0
OMM1181
6.6
OMM1160
8.7
OMM1544
0.0
OMM1426
11.3
Omy_tlr3-377
20.1
Omy17DIAS
36.0
OMM1549
OMM1413/i
47.1
Male 1
OMM1050
OMM1181
OMM1160
TCARRA(11vs12)/i
OMM1544
OMM1426
OMM5312
Omy_tlr3-377
Omy17DIAS
OMM1134/i
OMM1528/i
OMM1413/i
0.0
2.2
4.4
Male 2
OMM1181
OMM1160
OMM5108
OMM1544
OMM1426
Omy17DIAS
OMM1549
OMM1413/i
0.0

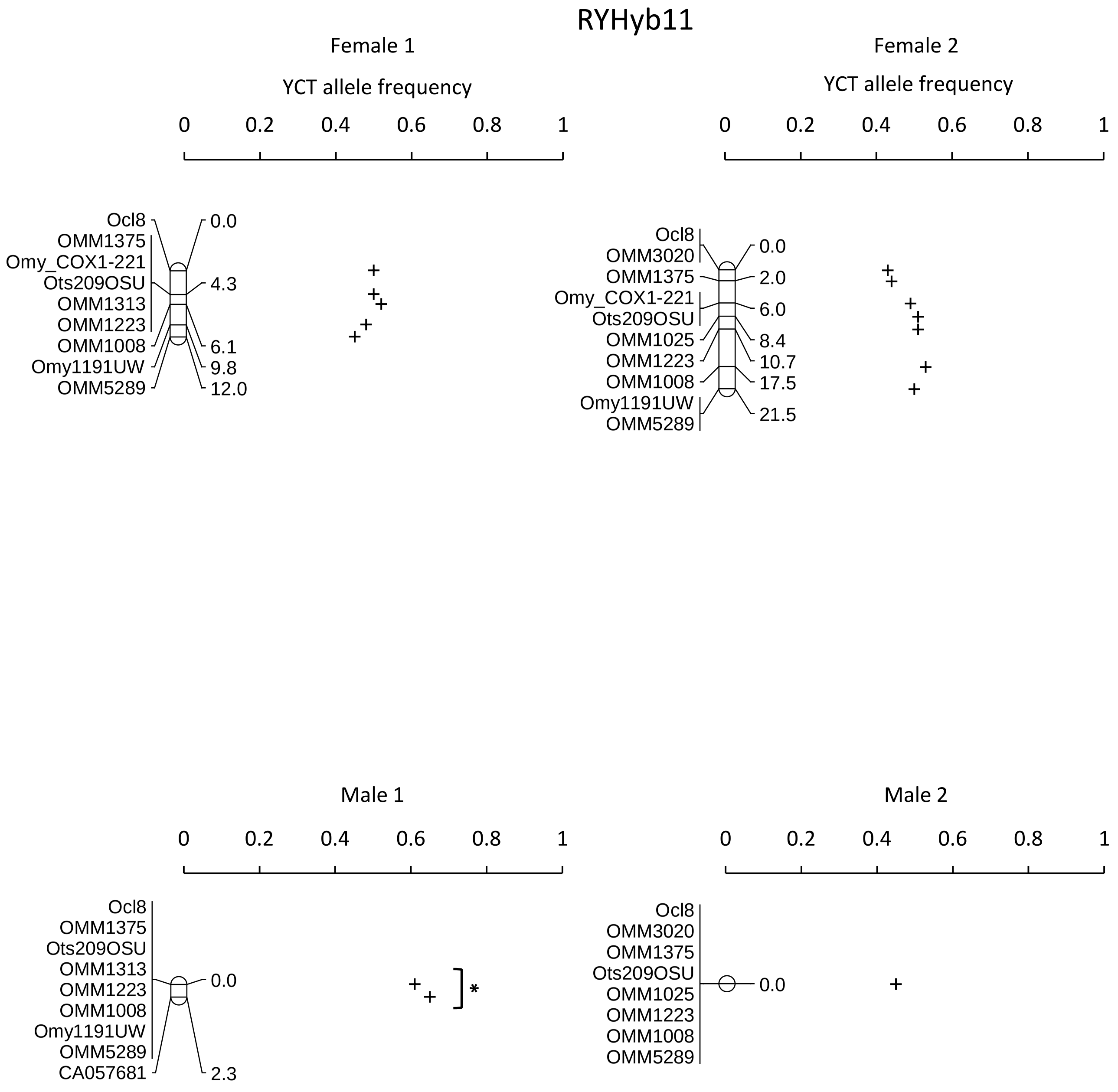

RYHyb11
Female 1
YCT allele frequency
0 0.2 0.4 0.6 0.8 1
Ocl8
OMM1375
Omy_COX1-221
Ots209OSU
OMM1313
OMM1223
OMM1008
Omy1191UW
OMM5289
0.0
4.3
6.1
9.8
12.0
Female 2
YCT allele frequency
0 0.2 0.4 0.6 0.8 1
Ocl8
OMM3020
OMM1375
Omy_COX1-221
Ots209OSU
OMM1025
OMM1223
OMM1008
Omy1191UW
OMM5289
0.0
2.0
6.0
8.4
10.7
17.5
21.5
Male 1
0 0.2 0.4 0.6 0.8 1
Ocl8
OMM1375
Ots209OSU
OMM1313
OMM1223
OMM1008
Omy1191UW
OMM5289
CA057681
0.0
2.3
*
Male 2
0 0.2 0.4 0.6 0.8 1
Ocl8
OMM3020
OMM1375
Ots209OSU
OMM1025
OMM1223
OMM1008
OMM5289
0.0

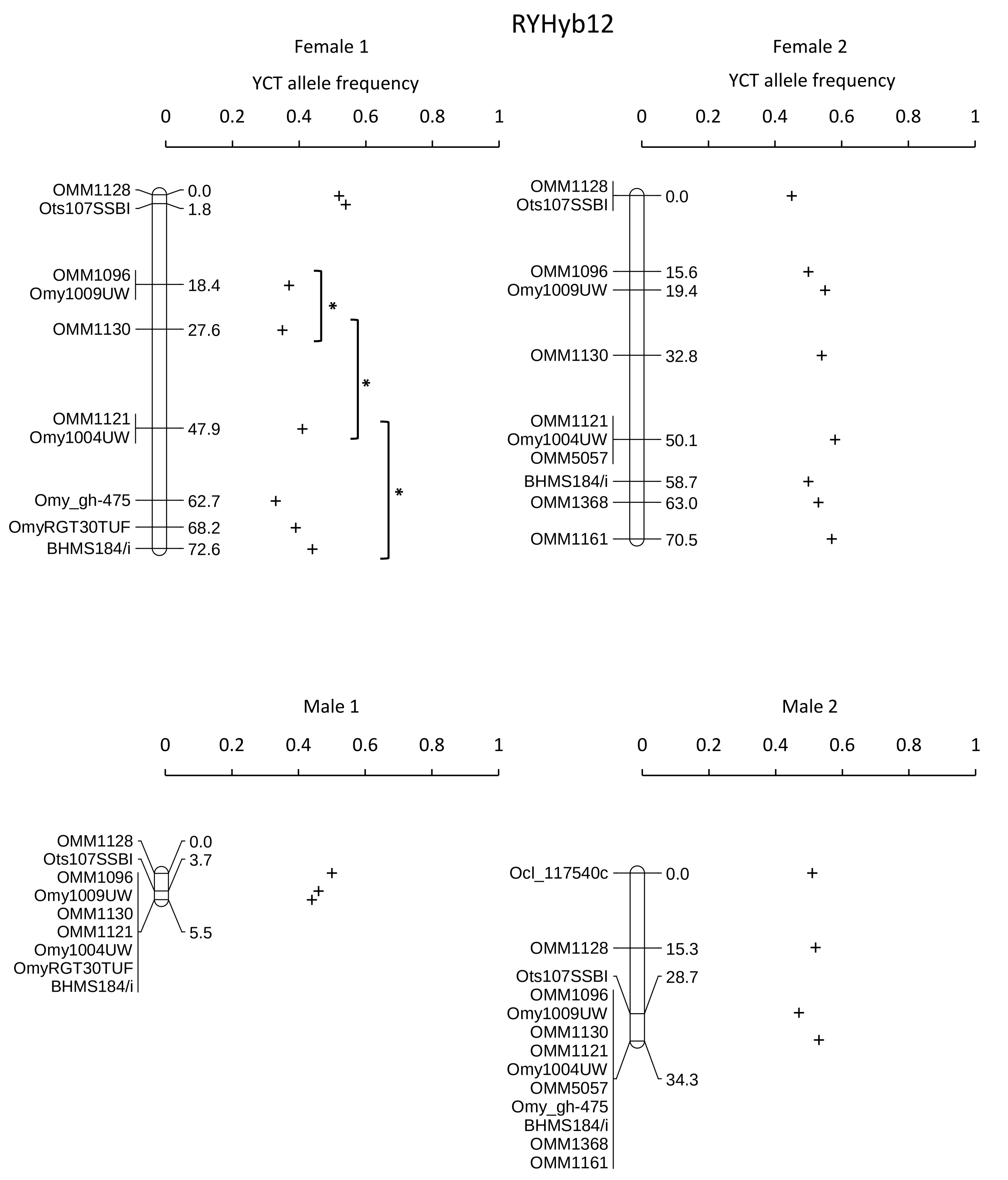
RYHyb12
Female 1
YCT allele frequency
0 0.2 0.4 0.6 0.8 1
OMM1128 0.0
Ots107SSBI 1.8
OMM1096
Omy1009UW 18.4
OMM1130 27.6
OMM1121
Omy1004UW 47.9
Omy_gh-475 62.7
OmyRGT30TUF 68.2
BHMS184/i 72.6
*
*
*
Female 2
YCT allele frequency
0 0.2 0.4 0.6 0.8 1
OMM1128
Ots107SSBI 0.0
OMM1096 15.6
Omy1009UW 19.4
OMM1130 32.8
OMM1121
Omy1004UW
OMM5057 50.1
BHMS184/i 58.7
OMM1368 63.0
OMM1161 70.5
Male 1
0 0.2 0.4 0.6 0.8 1
OMM1128 0.0
Ots107SSBI 3.7
OMM1096
Omy1009UW
OMM1130
OMM1121
Omy1004UW
OmyRGT30TUF
BHMS184/i 5.5
Male 2
0 0.2 0.4 0.6 0.8 1
Ocl_117540c 0.0
OMM1128 15.3
Ots107SSBI 28.7
OMM1096
Omy1009UW
OMM1130
OMM1121
Omy1004UW
OMM5057
Omy_gh-475
BHMS184/i
OMM1368
OMM1161 34.3

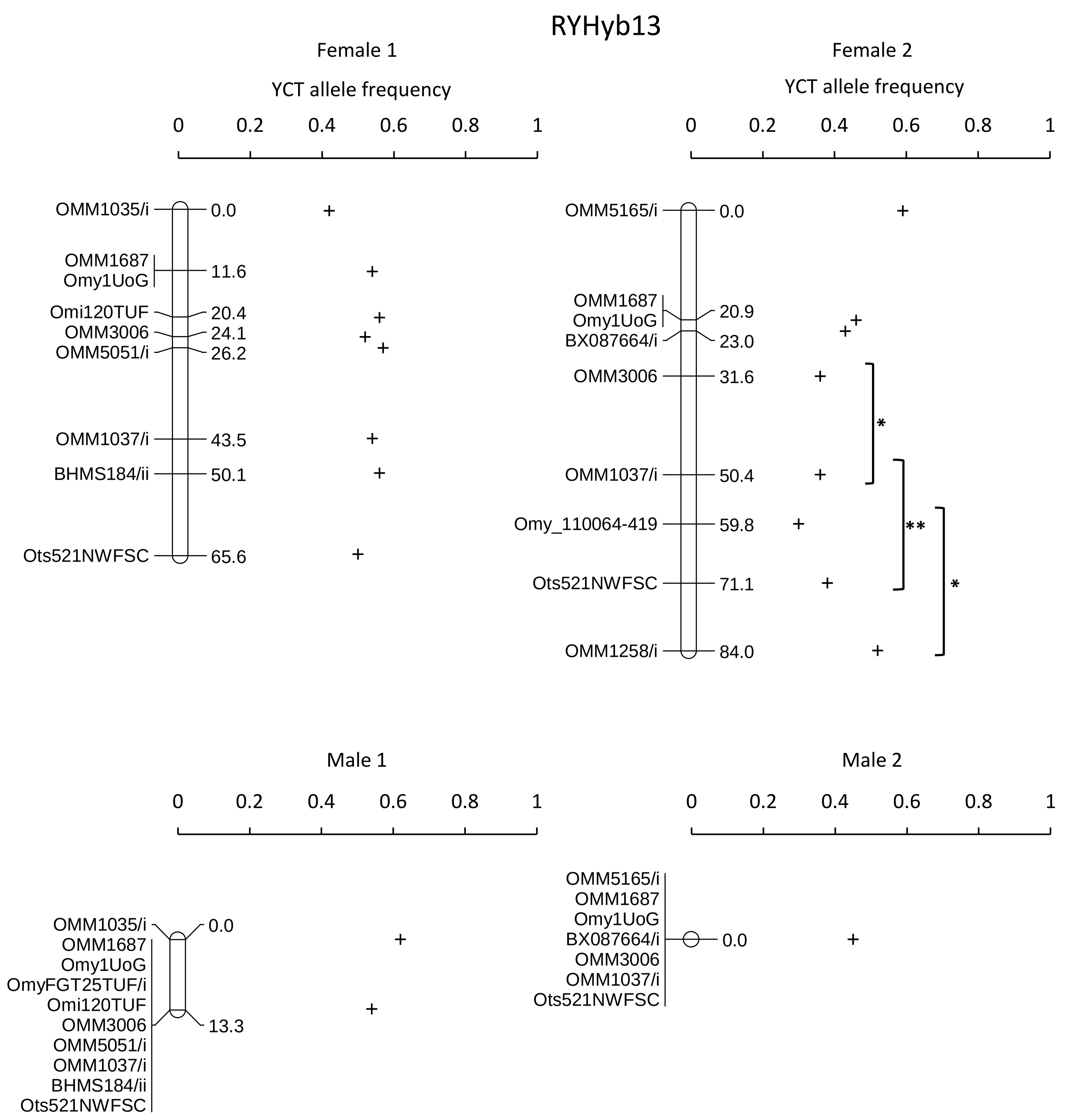
RYHyb13
Female 1
YCT allele frequency
0
0.2
0.4
0.6
0.8
1
OMM1035/i
0.0
OMM1687
Omy1UoG
11.6
Omi120TUF
20.4
OMM3006
24.1
OMM5051/i
26.2
OMM1037/i
43.5
BHMS184/ii
50.1
Ots521NWFSC
65.6
Female 2
YCT allele frequency
OMM5165/i
0.0
OMM1687
Omy1UoG
20.9
BX087664/i
23.0
OMM3006
31.6
OMM1037/i
50.4
Omy_110064-419
59.8
Ots521NWFSC
71.1
OMM1258/i
84.0
*
**
*
Male 1
OMM1035/i
0.0
OMM1687
Omy1UoG
OmyFGT25TUF/i
Omi120TUF
OMM3006
13.3
OMM5051/i
OMM1037/i
BHMS184/ii
Ots521NWFSC
Male 2
OMM5165/i
OMM1687
Omy1UoG
BX087664/i
0.0
OMM3006
OMM1037/i
Ots521NWFSC

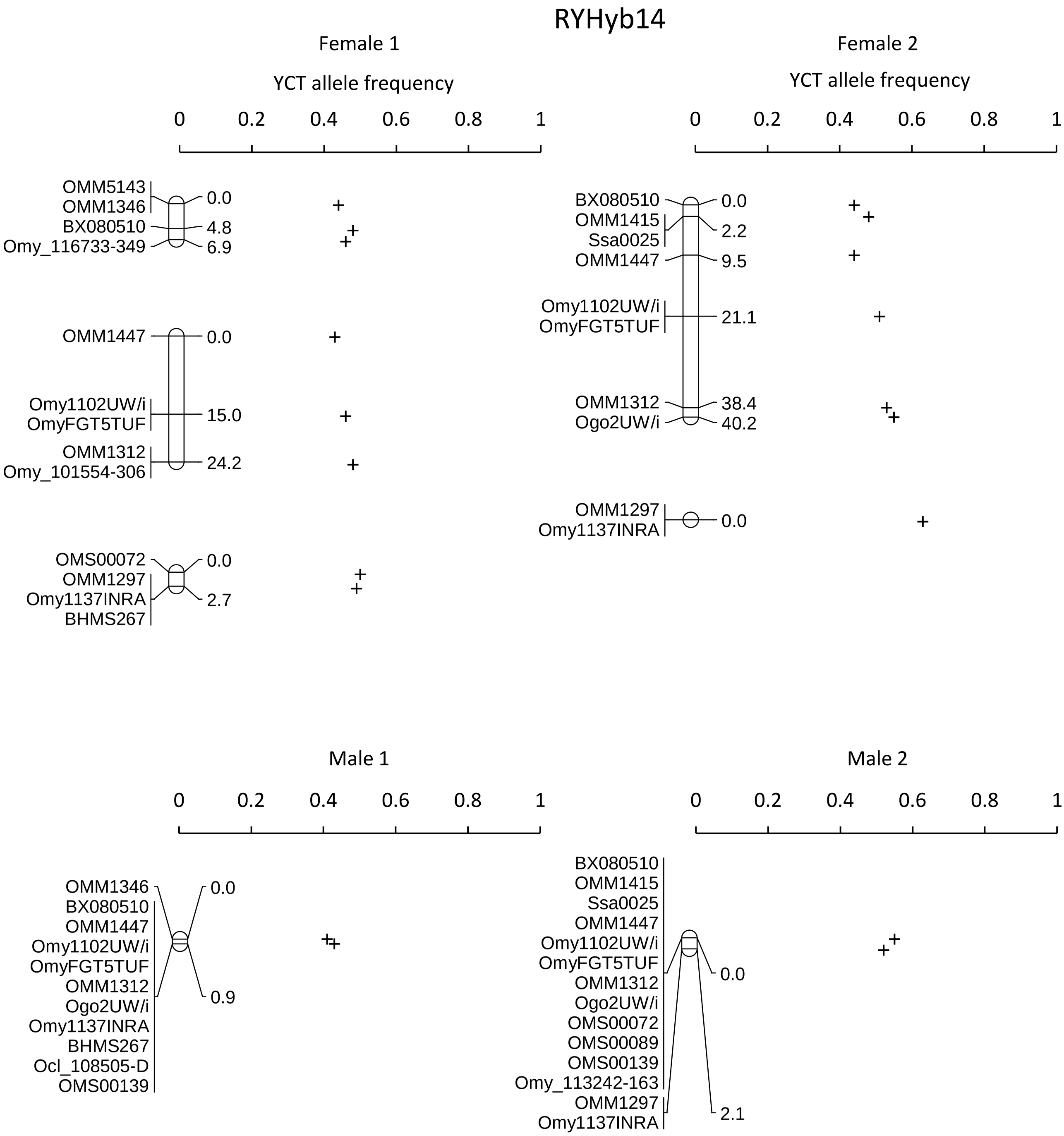
RYHyb14
Female 1
YCT allele frequency
0 0.2 0.4 0.6 0.8 1
OMM5143
OMM1346
BX080510
Omy_116733-349
0.0
4.8
6.9
OMM1447
Omy1102UW/i
OmyFGT5TUF
OMM1312
Omy_101554-306
0.0
15.0
24.2
OMS00072
OMM1297
Omy1137INRA
BHMS267
0.0
2.7
Female 2
YCT allele frequency
0 0.2 0.4 0.6 0.8 1
BX080510
OMM1415
Ssa0025
OMM1447
Omy1102UW/i
OmyFGT5TUF
OMM1312
Ogo2UW/i
0.0
2.2
9.5
21.1
38.4
40.2
OMM1297
Omy1137INRA
0.0
Male 1
0 0.2 0.4 0.6 0.8 1
OMM1346
BX080510
OMM1447
Omy1102UW/i
OmyFGT5TUF
OMM1312
Ogo2UW/i
Omy1137INRA
BHMS267
Ocl_108505-D
OMS00139
0.0
0.9
Male 2
0 0.2 0.4 0.6 0.8 1
BX080510
OMM1415
Ssa0025
OMM1447
Omy1102UW/i
OmyFGT5TUF
OMM1312
Ogo2UW/i
OMS00072
OMS00089
OMS00139
Omy_113242-163
OMM1297
Omy1137INRA
0.0
2.1

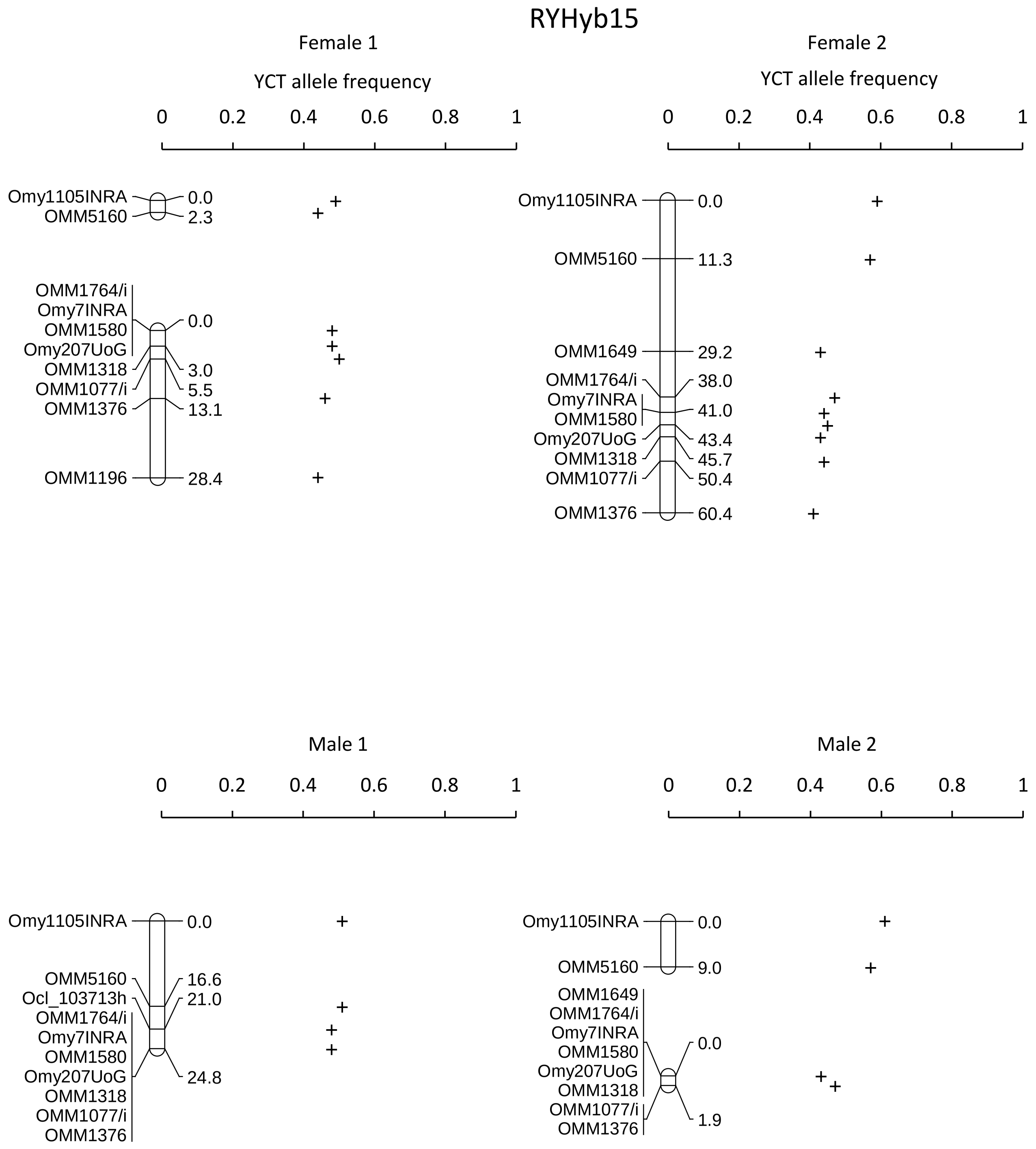
RYHyb15
Female 1
YCT allele frequency
0 0.2 0.4 0.6 0.8 1
Omy1105INRA 0.0
OMM5160 2.3
OMM1764/i
Omy7INRA
OMM1580 0.0
Omy207UoG
OMM1318 3.0
OMM1077/i 5.5
OMM1376 13.1
OMM1196 28.4
Female 2
YCT allele frequency
0 0.2 0.4 0.6 0.8 1
Omy1105INRA 0.0
OMM5160 11.3
OMM1649 29.2
OMM1764/i 38.0
Omy7INRA
OMM1580 41.0
Omy207UoG 43.4
OMM1318 45.7
OMM1077/i 50.4
OMM1376 60.4
Male 1
0 0.2 0.4 0.6 0.8 1
Omy1105INRA 0.0
OMM5160 16.6
Ocl_103713h 21.0
OMM1764/i
Omy7INRA
OMM1580
Omy207UoG 24.8
OMM1318
OMM1077/i
OMM1376
Male 2
0 0.2 0.4 0.6 0.8 1
Omy1105INRA 0.0
OMM5160 9.0
OMM1649
OMM1764/i
Omy7INRA
OMM1580 0.0
Omy207UoG
OMM1318
OMM1077/i
OMM1376 1.9

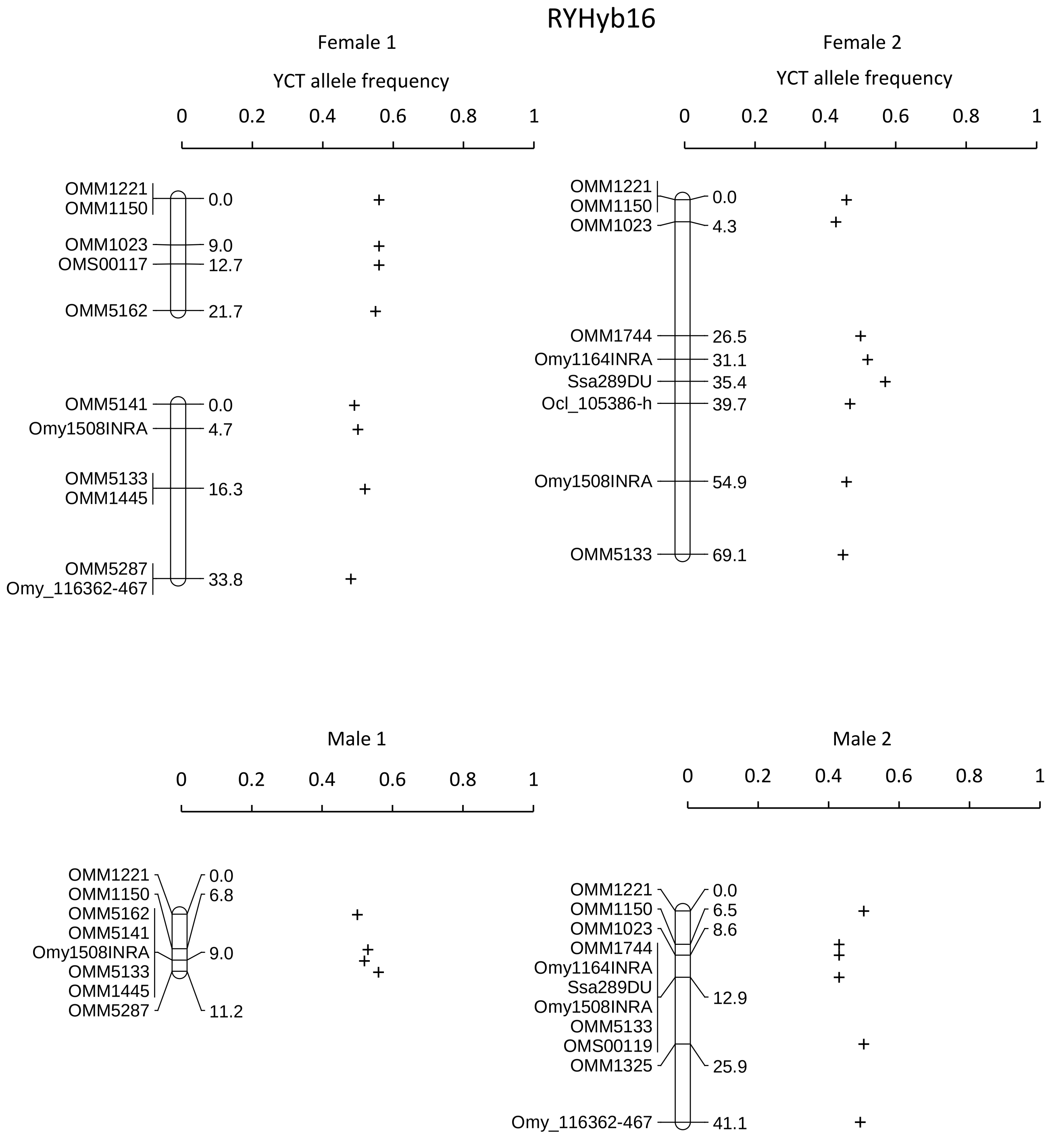
RYHyb16
Female 1
YCT allele frequency
0
0.2
0.4
0.6
0.8
1
OMM1221
OMM1150
0.0
OMM1023
9.0
OMS00117
12.7
OMM5162
21.7
OMM5141
0.0
Omy1508INRA
4.7
OMM5133
OMM1445
16.3
OMM5287
Omy_116362-467
33.8
Female 2
YCT allele frequency
OMM1221
OMM1150
0.0
OMM1023
4.3
OMM1744
26.5
Omy1164INRA
31.1
Ssa289DU
35.4
Ocl_105386-h
39.7
Omy1508INRA
54.9
OMM5133
69.1
Male 1
OMM1221
OMM1150
OMM5162
OMM5141
Omy1508INRA
OMM5133
OMM1445
OMM5287
0.0
6.8
9.0
11.2
Male 2
OMM1221
OMM1150
OMM1023
OMM1744
Omy1164INRA
Ssa289DU
Omy1508INRA
OMM5133
OMS00119
OMM1325
0.0
6.5
8.6
12.9
25.9
Omy_116362-467
41.1

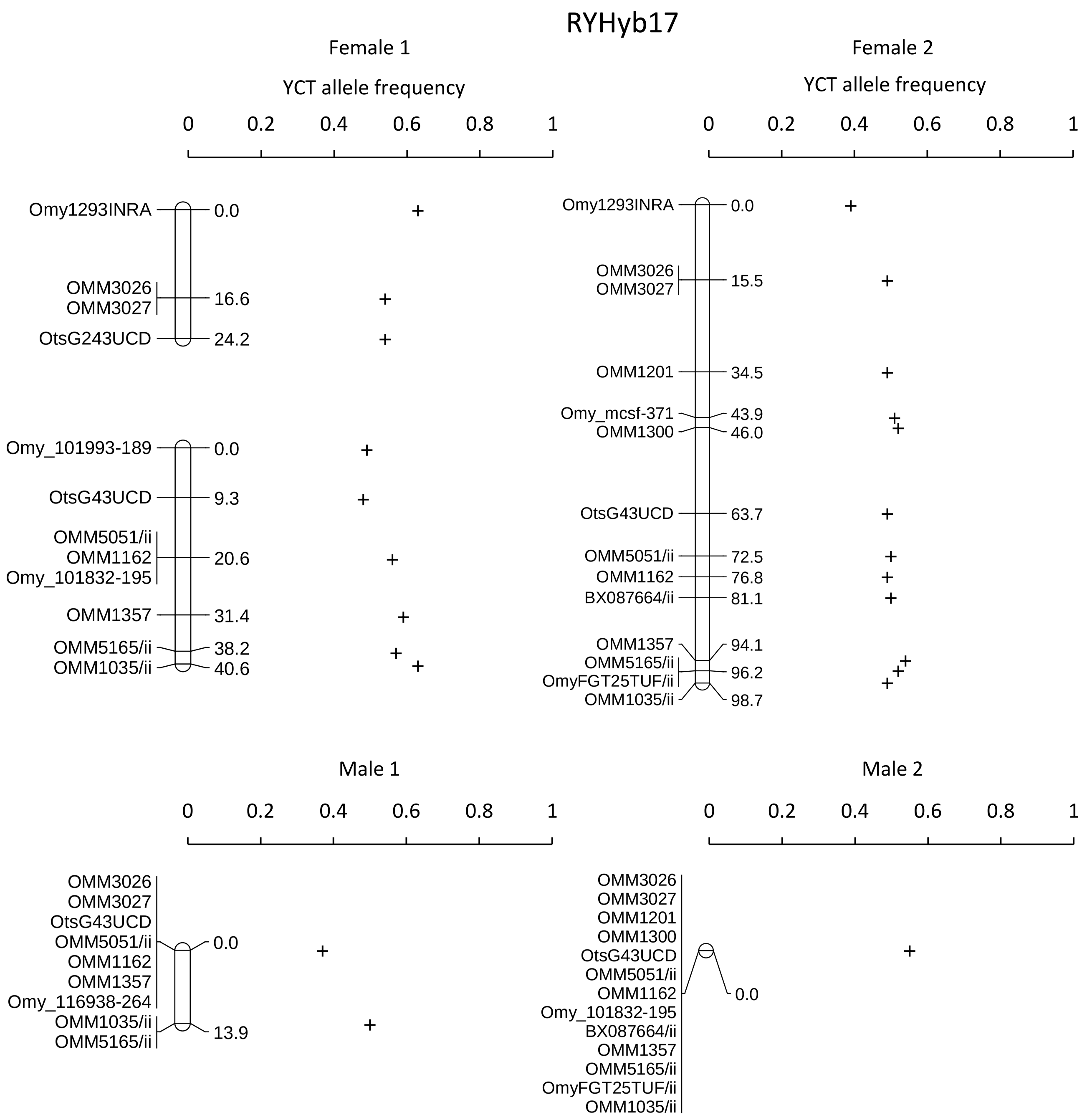
RYHyb17
Female 1
YCT allele frequency
0
0.2
0.4
0.6
0.8
1
Omy1293INRA
0.0
OMM3026
OMM3027
16.6
OtsG243UCD
24.2
Omy_101993-189
0.0
OtsG43UCD
9.3
OMM5051/ii
OMM1162
Omy_101832-195
20.6
OMM1357
31.4
OMM5165/ii
38.2
OMM1035/ii
40.6
Female 2
YCT allele frequency
Omy1293INRA
0.0
OMM3026
OMM3027
15.5
OMM1201
34.5
Omy_mcsf-371
43.9
OMM1300
46.0
OtsG43UCD
63.7
OMM5051/ii
72.5
OMM1162
76.8
BX087664/ii
81.1
OMM1357
94.1
OMM5165/ii
OmyFGT25TUF/ii
96.2
OMM1035/ii
98.7
Male 1
OMM3026
OMM3027
OtsG43UCD
OMM5051/ii
0.0
OMM1162
OMM1357
Omy_116938-264
OMM1035/ii
OMM5165/ii
13.9
Male 2
OMM3026
OMM3027
OMM1201
OMM1300
OtsG43UCD
OMM5051/ii
OMM1162
0.0
Omy_101832-195
BX087664/ii
OMM1357
OMM5165/ii
OmyFGT25TUF/ii
OMM1035/ii

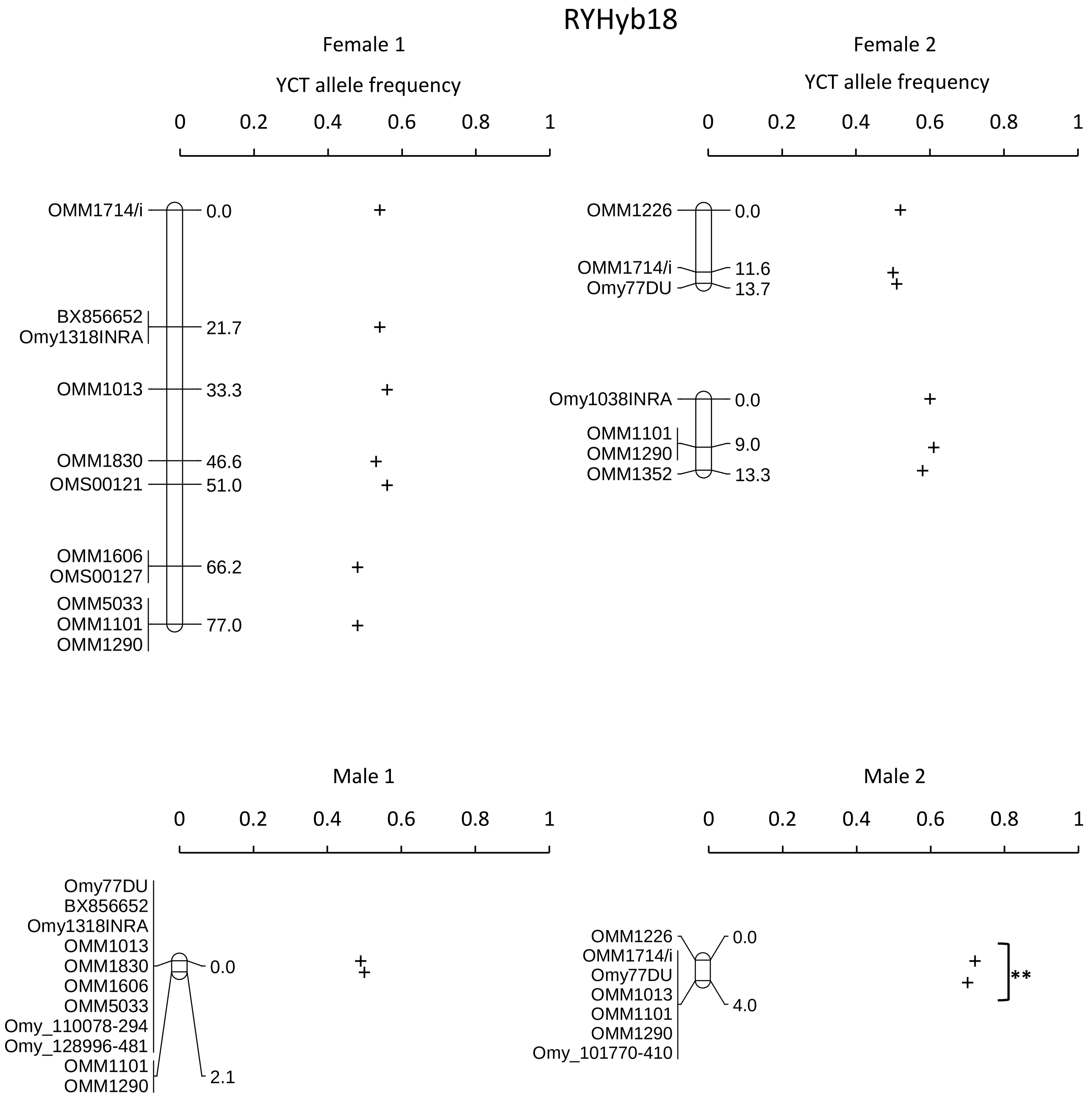
RYHyb18
Female 1
YCT allele frequency
0 0.2 0.4 0.6 0.8 1
OMM1714/i 0.0
BX856652
Omy1318INRA 21.7
OMM1013 33.3
OMM1830 46.6
OMS00121 51.0
OMM1606
OMS00127 66.2
OMM5033
OMM1101 77.0
OMM1290
Female 2
YCT allele frequency
0 0.2 0.4 0.6 0.8 1
OMM1226 0.0
OMM1714/i 11.6
Omy77DU 13.7
Omy1038INRA 0.0
OMM1101
OMM1290 9.0
OMM1352 13.3
Male 1
0 0.2 0.4 0.6 0.8 1
Omy77DU
BX856652
Omy1318INRA
OMM1013
OMM1830 0.0
OMM1606
OMM5033
Omy_110078-294
Omy_128996-481
OMM1101 2.1
OMM1290
Male 2
0 0.2 0.4 0.6 0.8 1
OMM1226 0.0
OMM1714/i
Omy77DU
OMM1013 4.0
OMM1101
OMM1290
Omy_101770-410
**

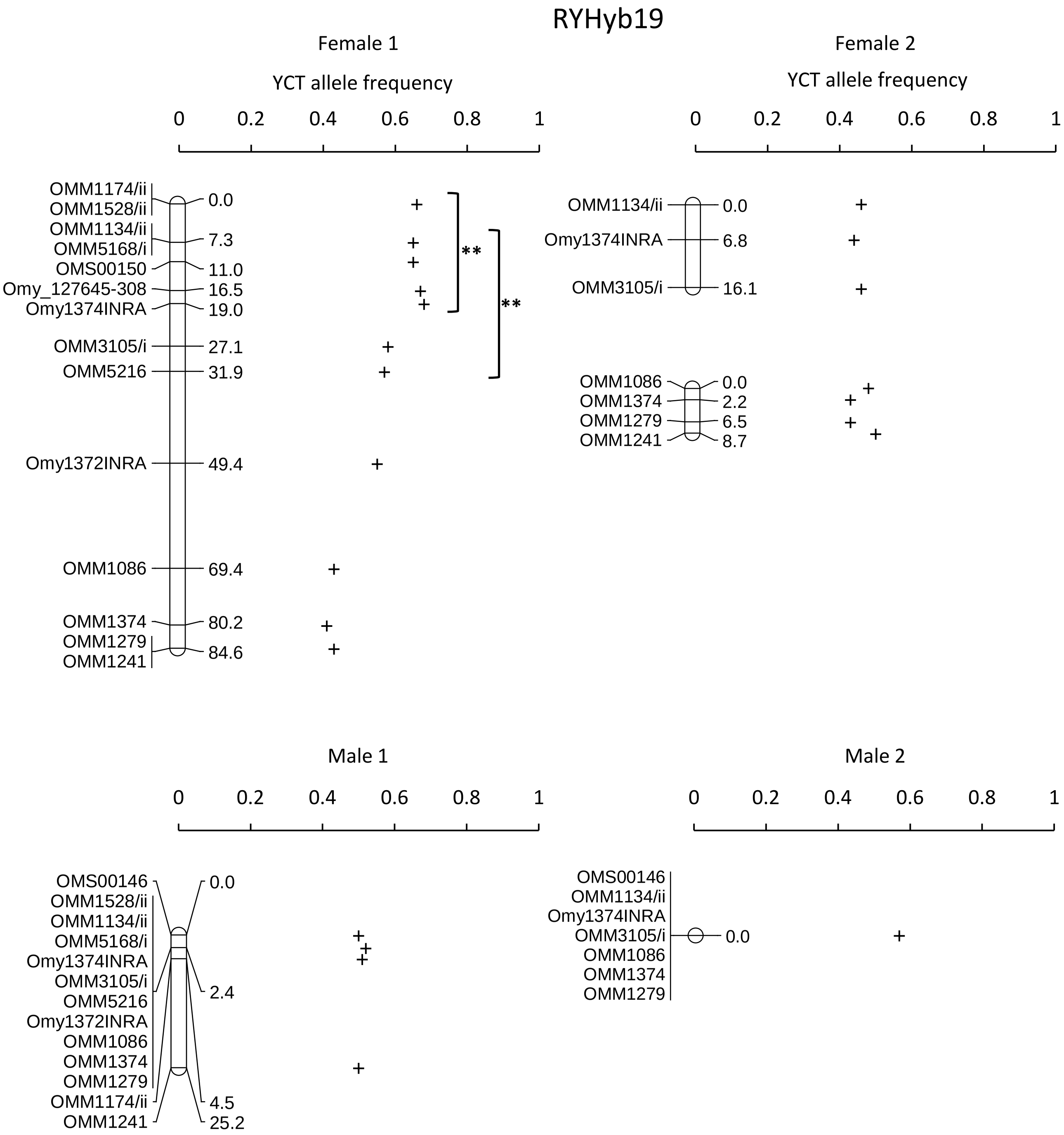
RYHyb19
Female 1
YCT allele frequency
0
0.2
0.4
0.6
0.8
1
OMM1174/ii
OMM1528/ii
OMM1134/ii
OMM5168/i
OMS00150
Omy_127645-308
Omy1374INRA
OMM3105/i
OMM5216
Omy1372INRA
OMM1086
OMM1374
OMM1279
OMM1241
0.0
7.3
11.0
16.5
19.0
27.1
31.9
49.4
69.4
80.2
84.6
**
**
Female 2
YCT allele frequency
0
0.2
0.4
0.6
0.8
1
OMM1134/ii
Omy1374INRA
OMM3105/i
0.0
6.8
16.1
OMM1086
OMM1374
OMM1279
OMM1241
0.0
2.2
6.5
8.7
Male 1
0
0.2
0.4
0.6
0.8
1
OMS00146
OMM1528/ii
OMM1134/ii
OMM5168/i
Omy1374INRA
OMM3105/i
OMM5216
Omy1372INRA
OMM1086
OMM1374
OMM1279
OMM1174/ii
OMM1241
0.0
2.4
4.5
25.2
Male 2
0
0.2
0.4
0.6
0.8
1
OMS00146
OMM1134/ii
Omy1374INRA
OMM3105/i
OMM1086
OMM1374
OMM1279
0.0

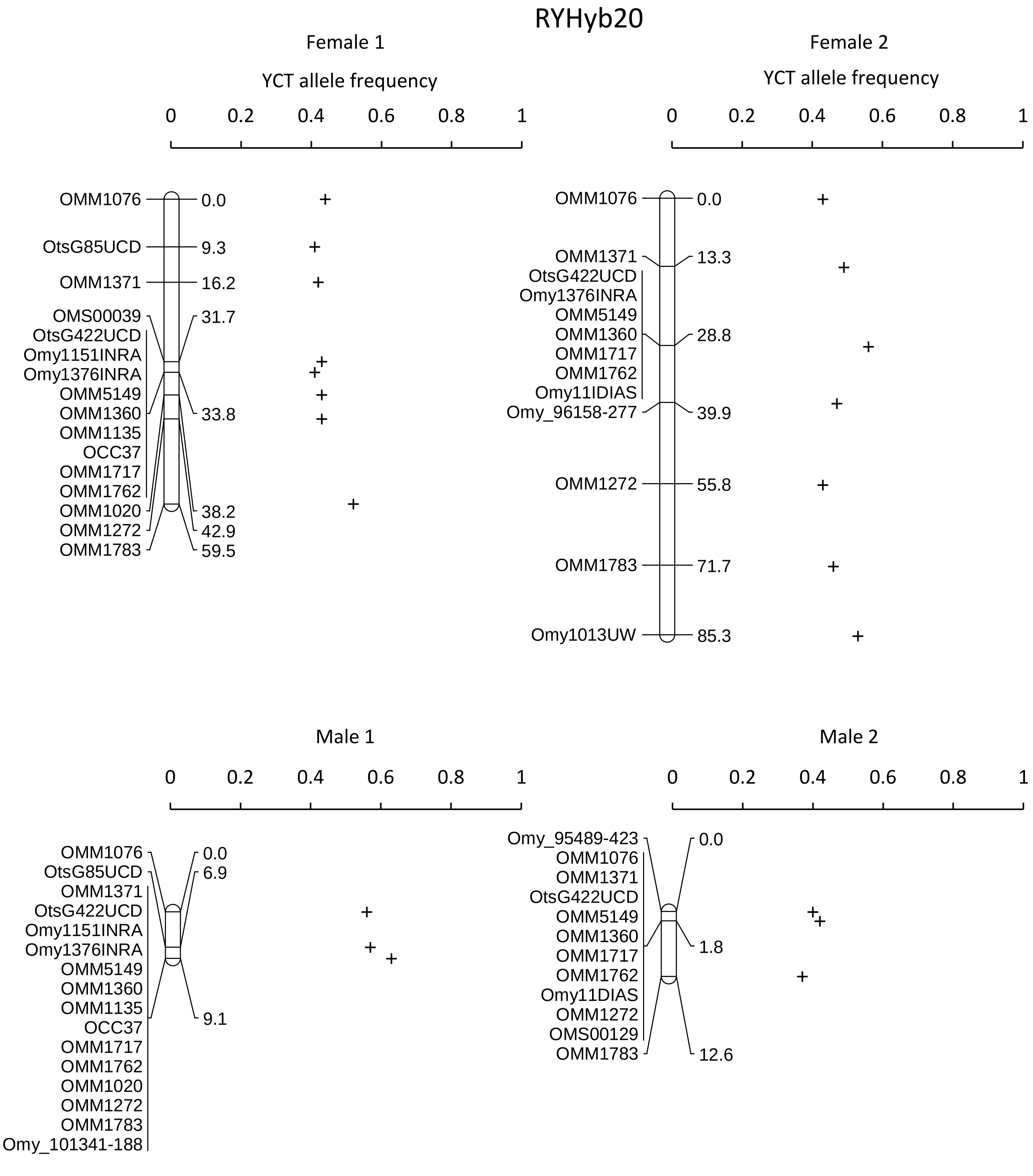
RYHyb20
Female 1
YCT allele frequency
0
0.2
0.4
0.6
0.8
1
OMM1076
OtsG85UCD
OMM1371
OMS00039
OtsG422UCD
Omy1151INRA
Omy1376INRA
OMM5149
OMM1360
OMM1135
OCC37
OMM1717
OMM1762
OMM1020
OMM1272
OMM1783
0.0
9.3
16.2
31.7
33.8
38.2
42.9
59.5
Female 2
YCT allele frequency
OMM1076
OMM1371
OtsG422UCD
Omy1376INRA
OMM5149
OMM1360
OMM1717
OMM1762
Omy11IDIAS
Omy_96158-277
OMM1272
OMM1783
Omy1013UW
0.0
13.3
28.8
39.9
55.8
71.7
85.3
Male 1
OMM1076
OtsG85UCD
OMM1371
OtsG422UCD
Omy1151INRA
Omy1376INRA
OMM5149
OMM1360
OMM1135
OCC37
OMM1717
OMM1762
OMM1020
OMM1272
OMM1783
Omy_101341-188
0.0
6.9
9.1
Male 2
Omy_95489-423
OMM1076
OMM1371
OtsG422UCD
OMM5149
OMM1360
OMM1717
OMM1762
Omy11DIAS
OMM1272
OMS00129
OMM1783
0.0
1.8
12.6

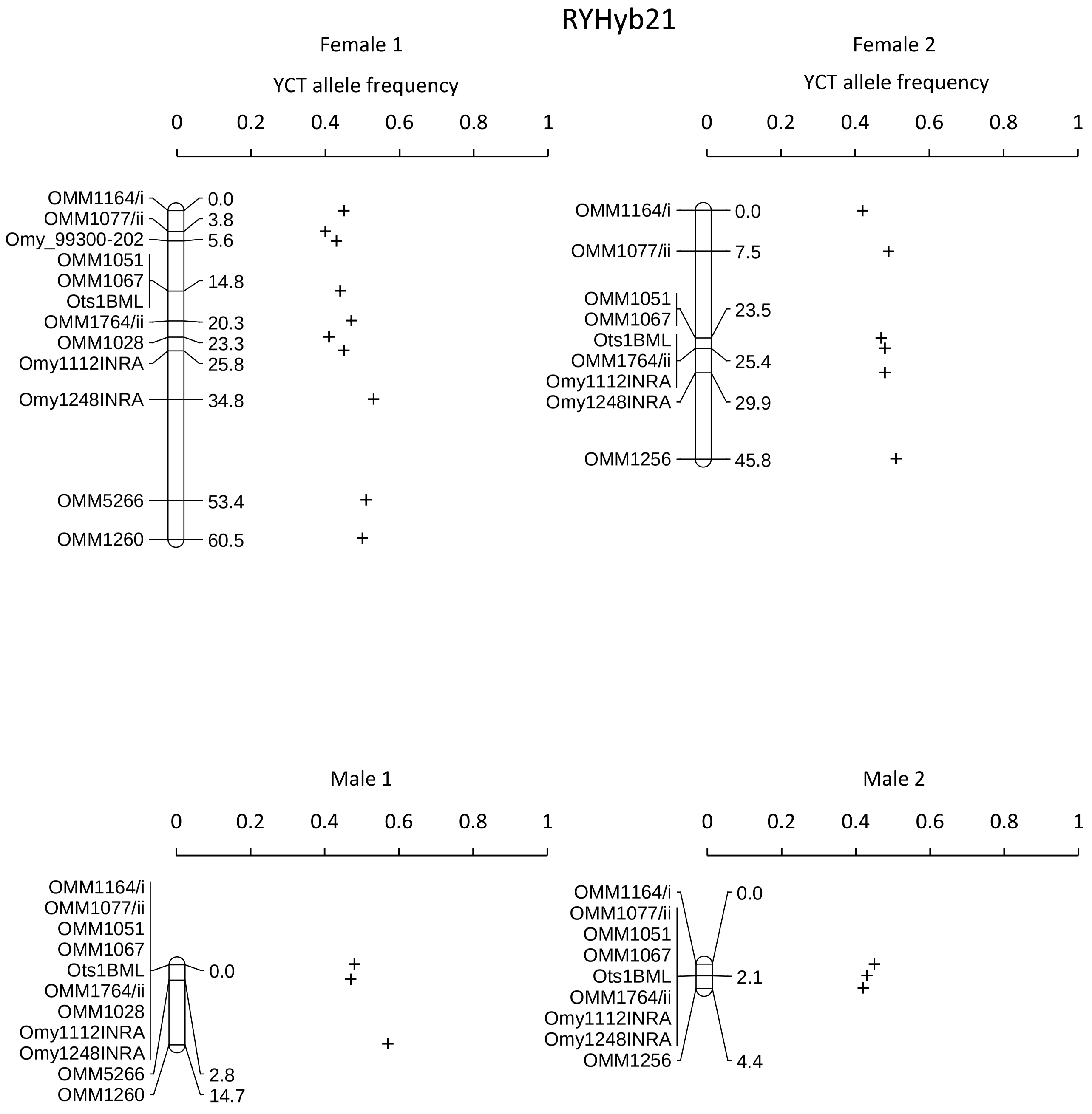
RYHyb21
Female 1
YCT allele frequency
0
0.2
0.4
0.6
0.8
1
OMM1164/i
OMM1077/ii
Omy_99300-202
OMM1051
OMM1067
Ots1BML
OMM1764/ii
OMM1028
Omy1112INRA
Omy1248INRA
OMM5266
OMM1260
0.0
3.8
5.6
14.8
20.3
23.3
25.8
34.8
53.4
60.5
Female 2
YCT allele frequency
OMM1164/i
OMM1077/ii
OMM1051
OMM1067
Ots1BML
OMM1764/ii
Omy1112INRA
Omy1248INRA
OMM1256
0.0
7.5
23.5
25.4
29.9
45.8
Male 1
OMM1164/i
OMM1077/ii
OMM1051
OMM1067
Ots1BML
OMM1764/ii
OMM1028
Omy1112INRA
Omy1248INRA
OMM5266
OMM1260
0.0
2.8
14.7
Male 2
OMM1164/i
OMM1077/ii
OMM1051
OMM1067
Ots1BML
OMM1764/ii
Omy1112INRA
Omy1248INRA
OMM1256
0.0
2.1
4.4

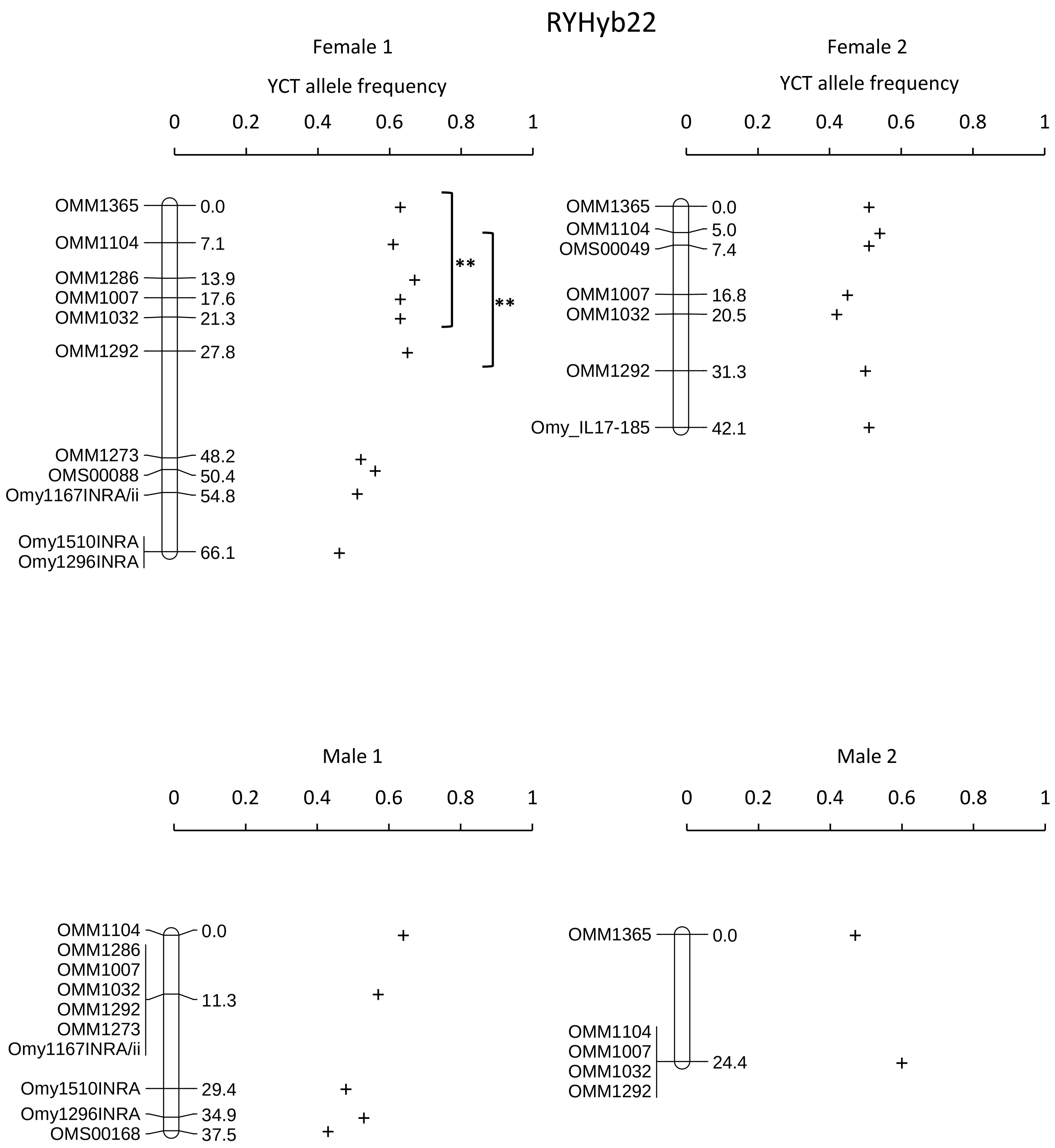
RYHyb22
Female 1
YCT allele frequency
0
0.2
0.4
0.6
0.8
1
OMM1365
0.0
OMM1104
7.1
OMM1286
13.9
OMM1007
17.6
OMM1032
21.3
OMM1292
27.8
OMM1273
48.2
OMS00088
50.4
Omy1167INRA/ii
54.8
Omy1510INRA
Omy1296INRA
66.1
**
**
Female 2
YCT allele frequency
0
0.2
0.4
0.6
0.8
1
OMM1365
0.0
OMM1104
5.0
OMS00049
7.4
OMM1007
16.8
OMM1032
20.5
OMM1292
31.3
Omy_IL17-185
42.1
Male 1
0
0.2
0.4
0.6
0.8
1
OMM1104
0.0
OMM1286
OMM1007
OMM1032
OMM1292
OMM1273
Omy1167INRA/ii
11.3
Omy1510INRA
29.4
Omy1296INRA
34.9
OMS00168
37.5
Male 2
0
0.2
0.4
0.6
0.8
1
OMM1365
0.0
OMM1104
OMM1007
OMM1032
OMM1292
24.4

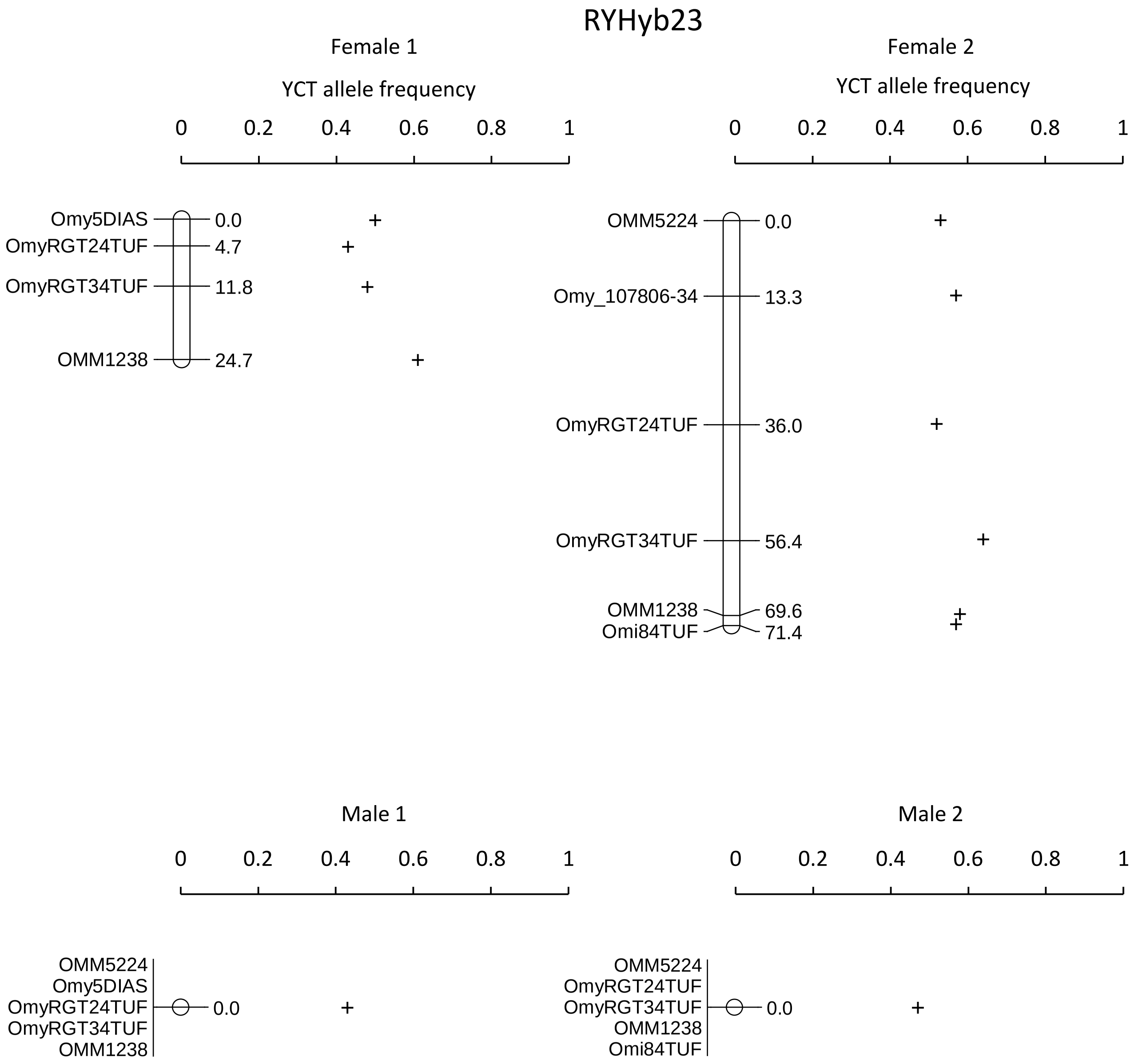
RYHyb23
Female 1
YCT allele frequency
0 0.2 0.4 0.6 0.8 1
Omy5DIAS 0.0
OmyRGT24TUF 4.7
OmyRGT34TUF 11.8
OMM1238 24.7
Female 2
YCT allele frequency
0 0.2 0.4 0.6 0.8 1
OMM5224 0.0
Omy_107806-34 13.3
OmyRGT24TUF 36.0
OmyRGT34TUF 56.4
OMM1238 69.6
Omi84TUF 71.4
Male 1
0 0.2 0.4 0.6 0.8 1
OMM5224
Omy5DIAS
OmyRGT24TUF
OmyRGT34TUF
OMM1238
0.0
Male 2
0 0.2 0.4 0.6 0.8 1
OMM5224
OmyRGT24TUF
OmyRGT34TUF
OMM1238
Omi84TUF
0.0

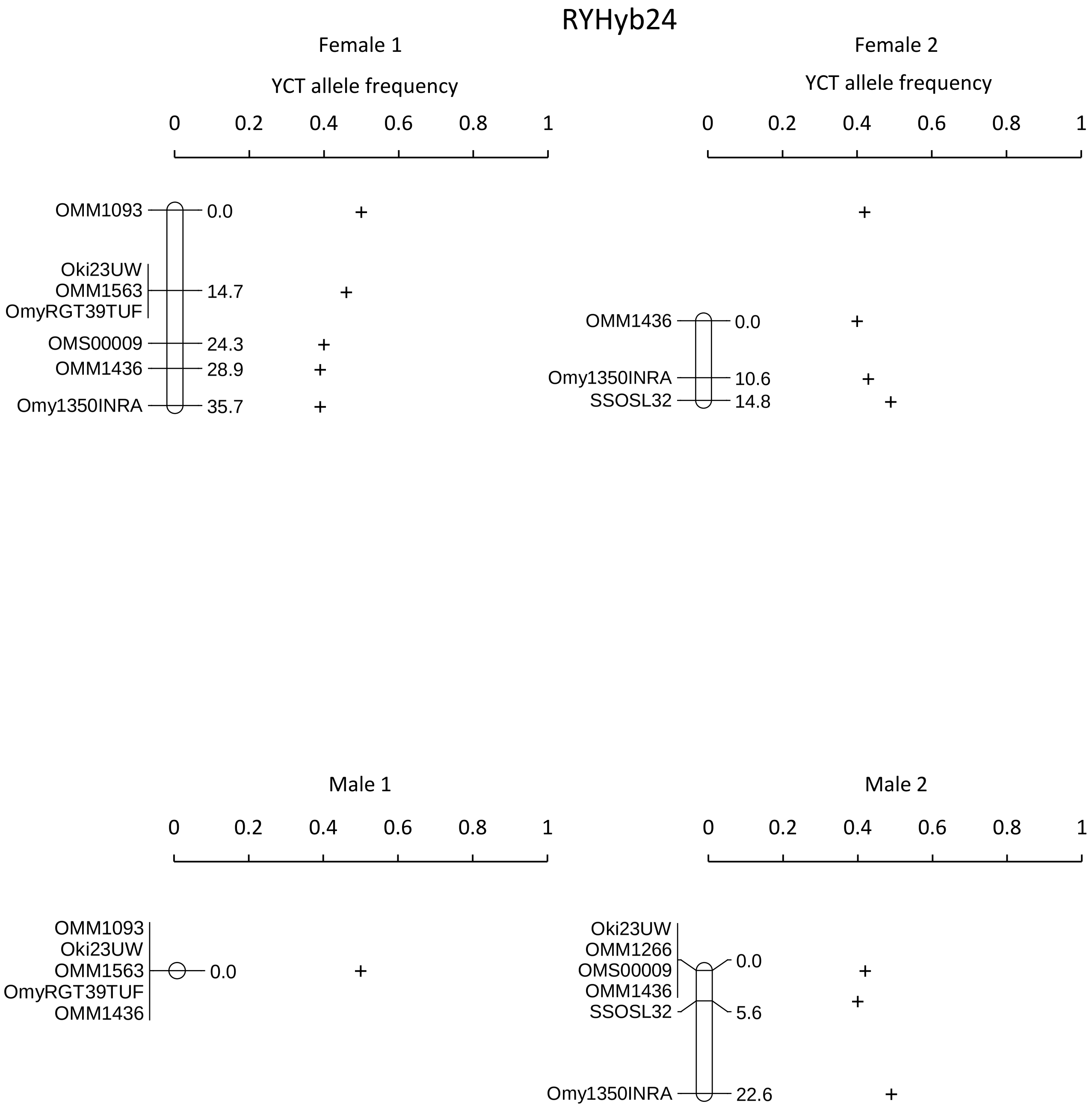

RYHyb24
Female 1
YCT allele frequency
0
0.2
0.4
0.6
0.8
1
OMM1093
0.0
Oki23UW
OMM1563
OmyRGT39TUF
14.7
OMS00009
24.3
OMM1436
28.9
Omy1350INRA
35.7
Female 2
YCT allele frequency
OMM1436
0.0
Omy1350INRA
10.6
SSOSL32
14.8
Male 1
OMM1093
Oki23UW
OMM1563
OmyRGT39TUF
OMM1436
0.0
Male 2
Oki23UW
OMM1266
OMS00009
OMM1436
0.0
SSOSL32
5.6
Omy1350INRA
22.6

RYHyb25 and RYHyb29 polymorphism

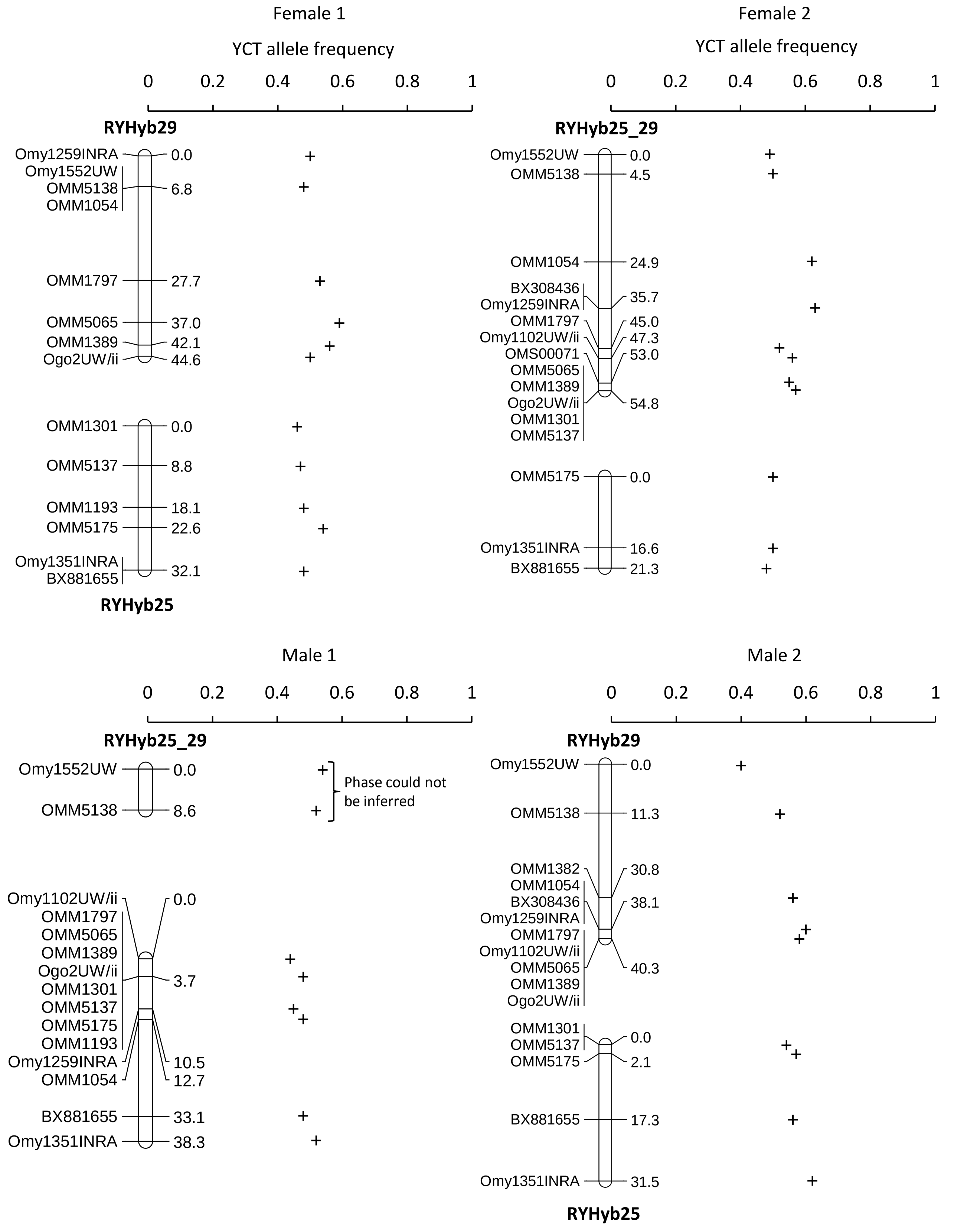

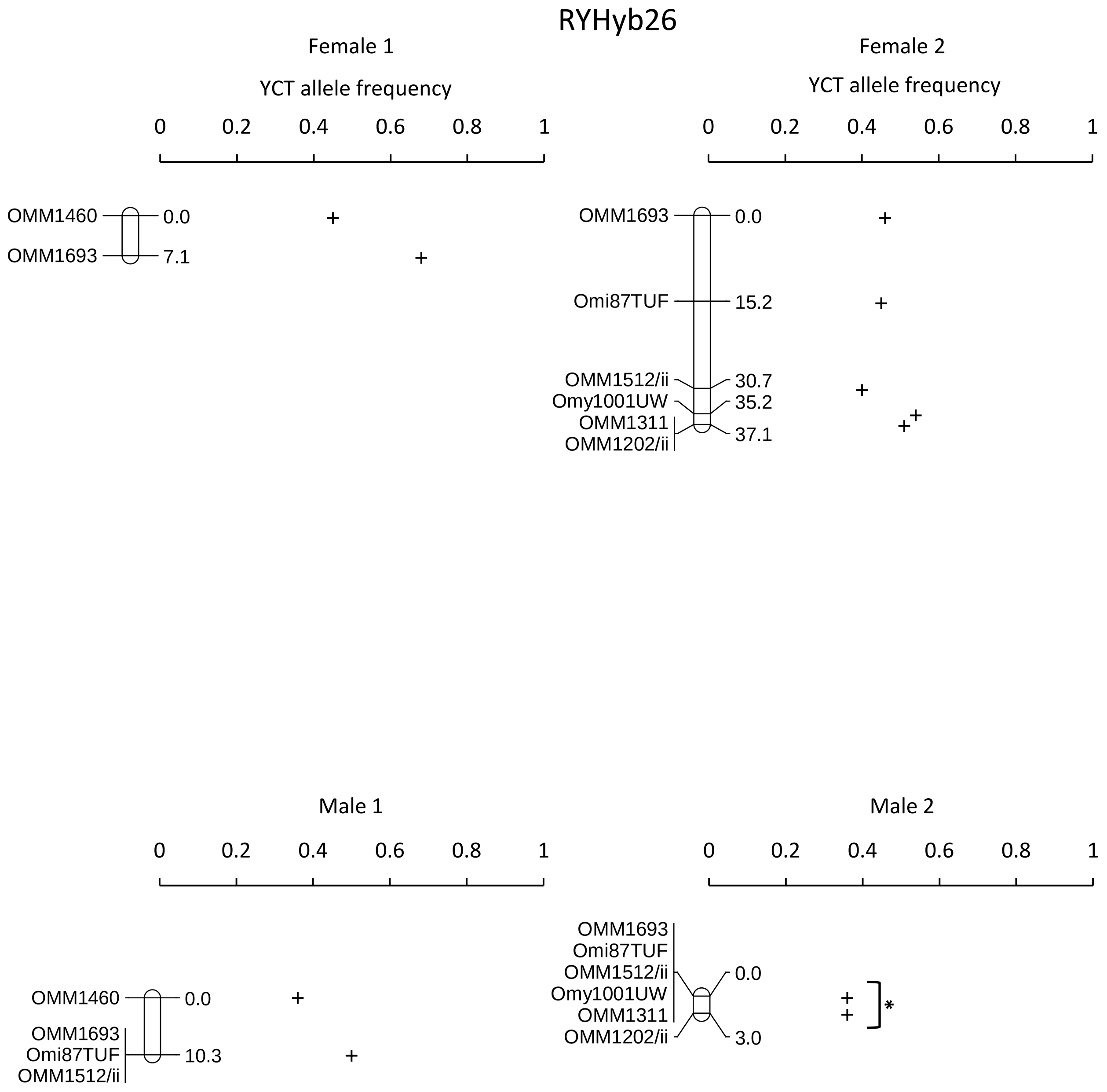
RYHyb26
Female 1
YCT allele frequency
0 0.2 0.4 0.6 0.8 1
OMM1460 0.0
OMM1693 7.1
Female 2
YCT allele frequency
0 0.2 0.4 0.6 0.8 1
OMM1693 0.0
Omi87TUF 15.2
OMM1512/ii 30.7
Omy1001UW 35.2
OMM1311
OMM1202/ii 37.1
Male 1
0 0.2 0.4 0.6 0.8 1
OMM1460 0.0
OMM1693
Omi87TUF 10.3
OMM1512/ii
Male 2
0 0.2 0.4 0.6 0.8 1
OMM1693
Omi87TUF
OMM1512/ii 0.0
Omy1001UW
OMM1311
OMM1202/ii 3.0
*

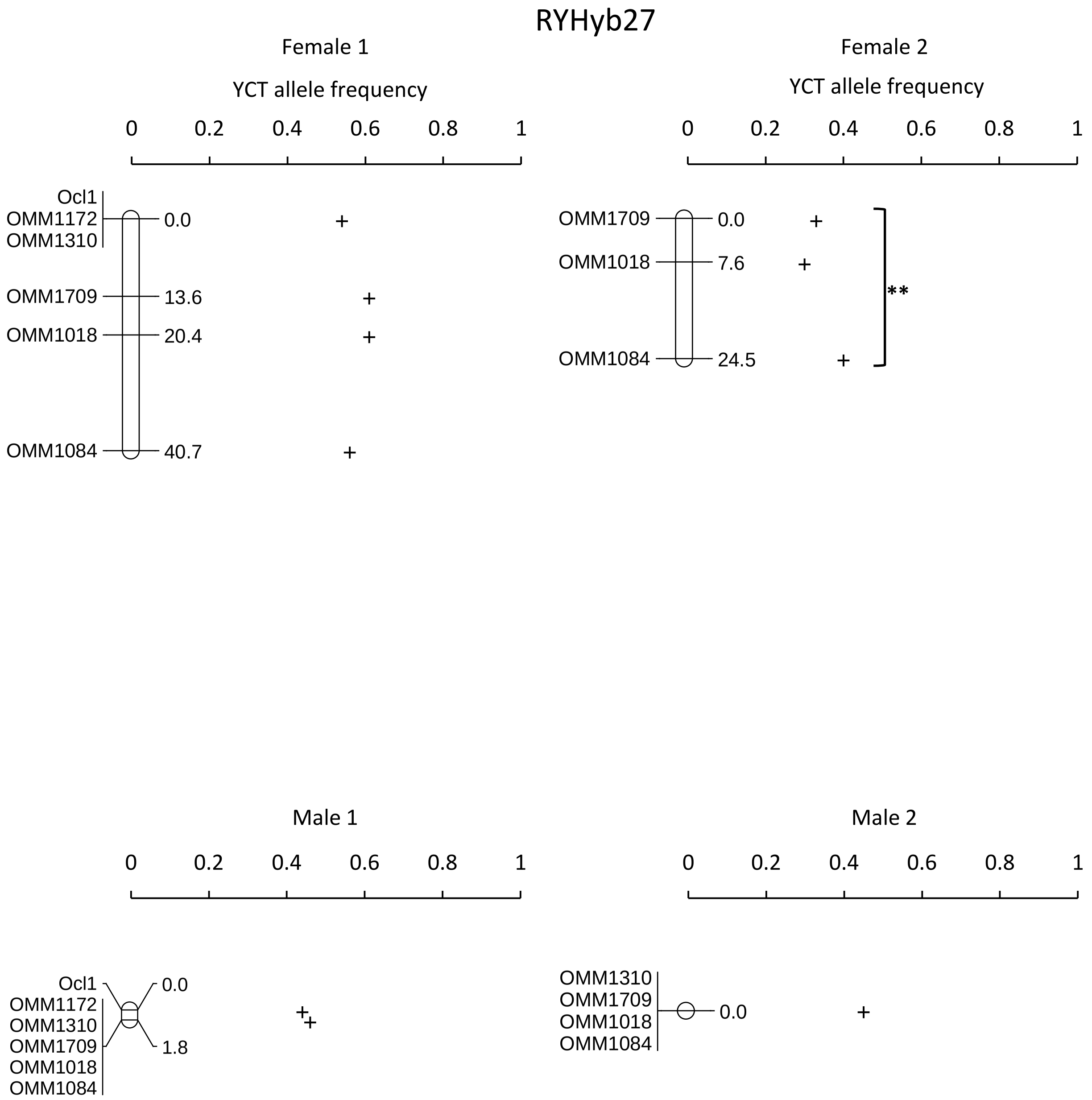

RYHyb27
Female 1
YCT allele frequency
0
0.2
0.4
0.6
0.8
1
Ocl1
OMM1172
OMM1310
0.0
OMM1709
13.6
OMM1018
20.4
OMM1084
40.7
Female 2
YCT allele frequency
0
0.2
0.4
0.6
0.8
1
OMM1709
0.0
OMM1018
7.6
OMM1084
24.5
**
Male 1
0
0.2
0.4
0.6
0.8
1
Ocl1
OMM1172
OMM1310
OMM1709
OMM1018
OMM1084
0.0
1.8
Male 2
0
0.2
0.4
0.6
0.8
1
OMM1310
OMM1709
OMM1018
OMM1084
0.0

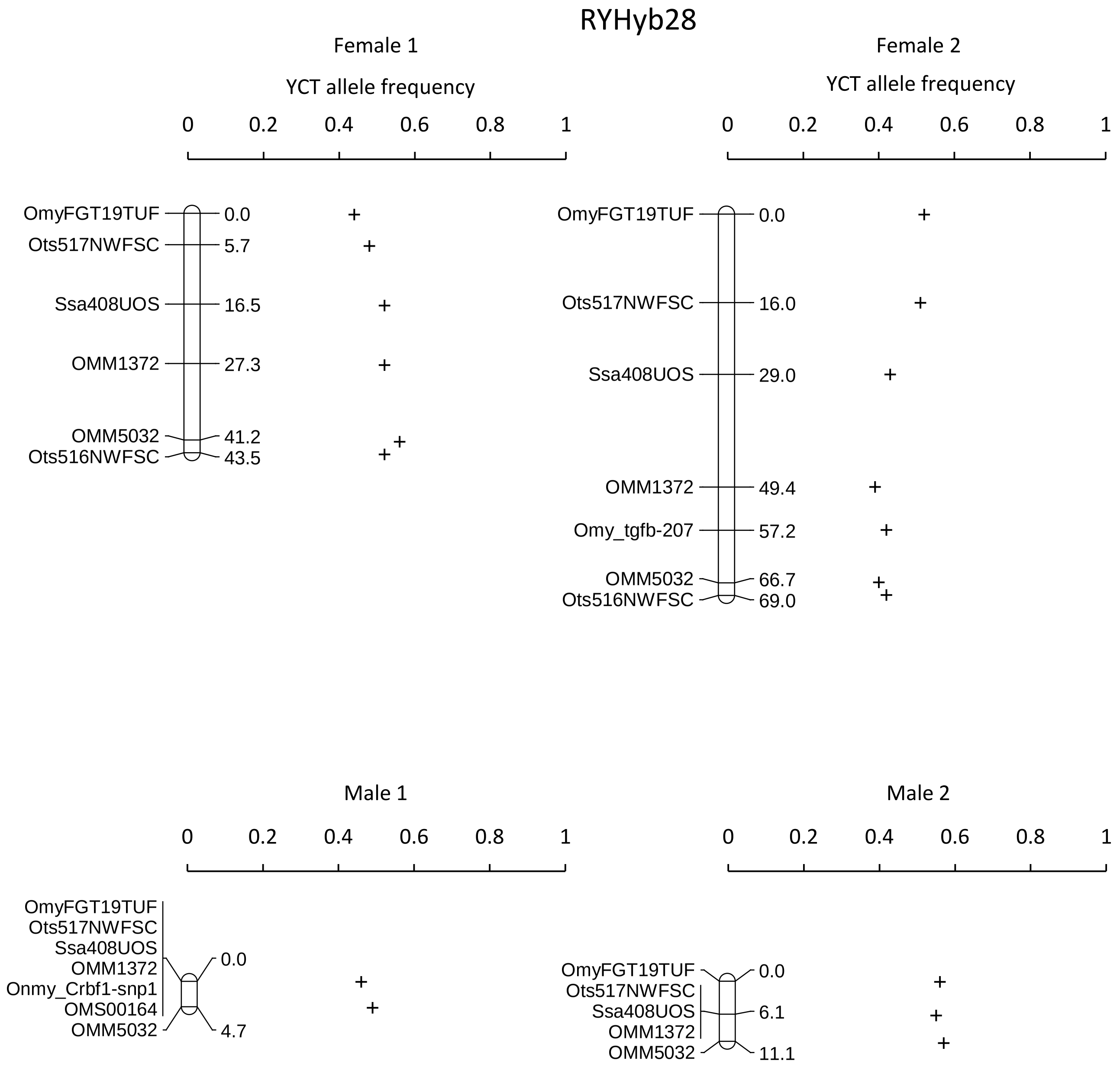
RYHyb28
Female 1
YCT allele frequency
0 0.2 0.4 0.6 0.8 1
OmyFGT19TUF 0.0
Ots517NWFSC 5.7
Ssa408UOS 16.5
OMM1372 27.3
OMM5032 41.2
Ots516NWFSC 43.5
Female 2
YCT allele frequency
0 0.2 0.4 0.6 0.8 1
OmyFGT19TUF 0.0
Ots517NWFSC 16.0
Ssa408UOS 29.0
OMM1372 49.4
Omy_tgfb-207 57.2
OMM5032 66.7
Ots516NWFSC 69.0
Male 1
0 0.2 0.4 0.6 0.8 1
OmyFGT19TUF
Ots517NWFSC
Ssa408UOS
OMM1372
Onmy_Crbf1-snp1
OMS00164
OMM5032
0.0
4.7
Male 2
0 0.2 0.4 0.6 0.8 1
OmyFGT19TUF 0.0
Ots517NWFSC
Ssa408UOS 6.1
OMM1372
OMM5032 11.1
