# Supplementary material for: Chromosome rearrangements, recombination suppression, and limited segregation distortion in hybrids between Yellowstone cutthroat trout (Oncorhynchus clarkii bouvieri) and rainbow trout (O. mykiss)
Source: BMC Genomics. 2013 Aug 22;14:570. doi: 10.1186/1471-2164-14-570 (PMC3765842; doi:10.1186/1471-2164-14-570)

Additional file 3. Female-, male-, and sex-merged F_1 hybrid linkage maps in *p* to *q* orientation with rainbow trout chromosome designations (Omy) and linkage groups (RT) indicated in parentheses. Map distances (cM) represent the recombination fraction (theta) between loci.

RYHyb01 (Omy1, RT6)

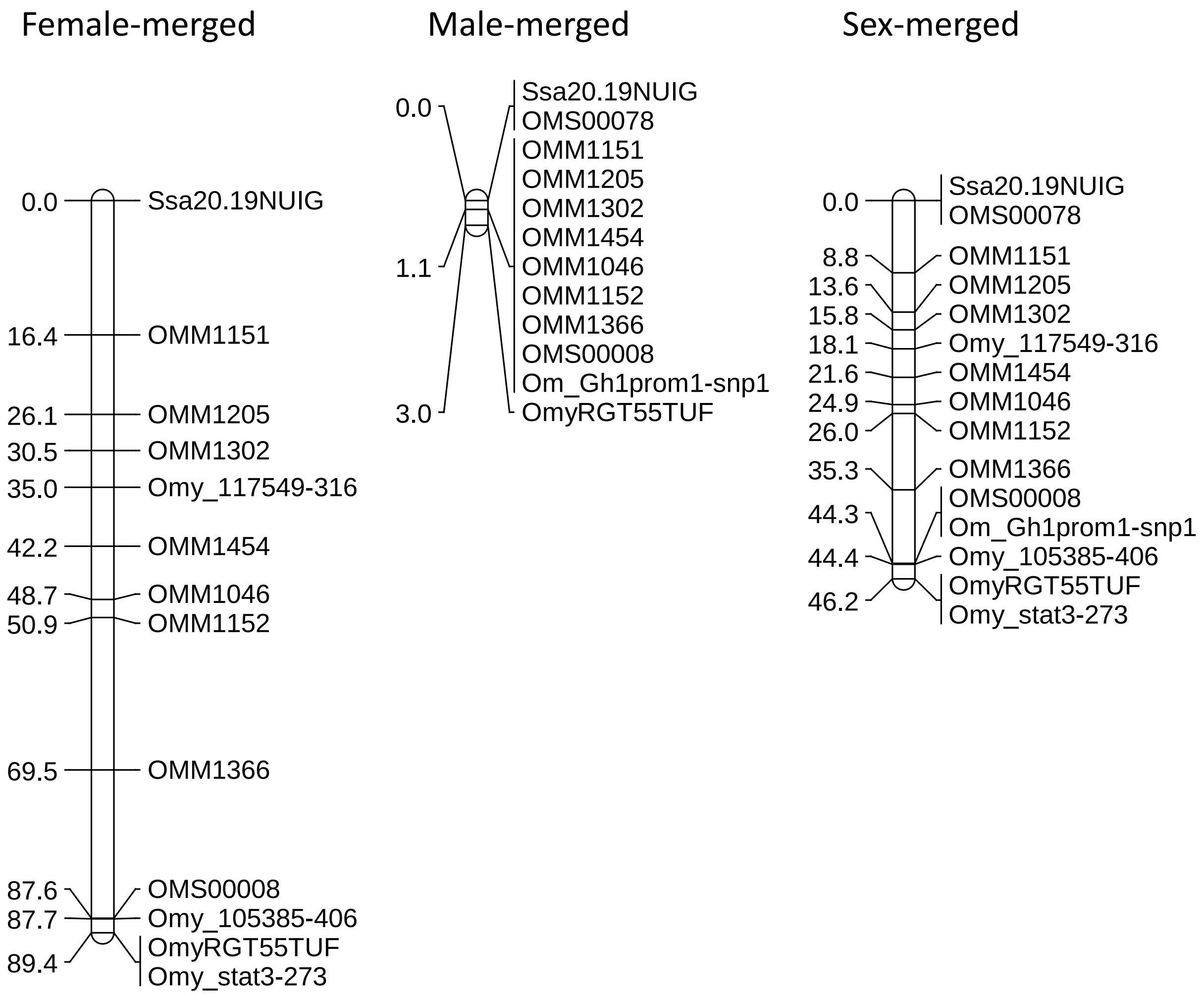

RYHyb02 (Omy2, RT27)

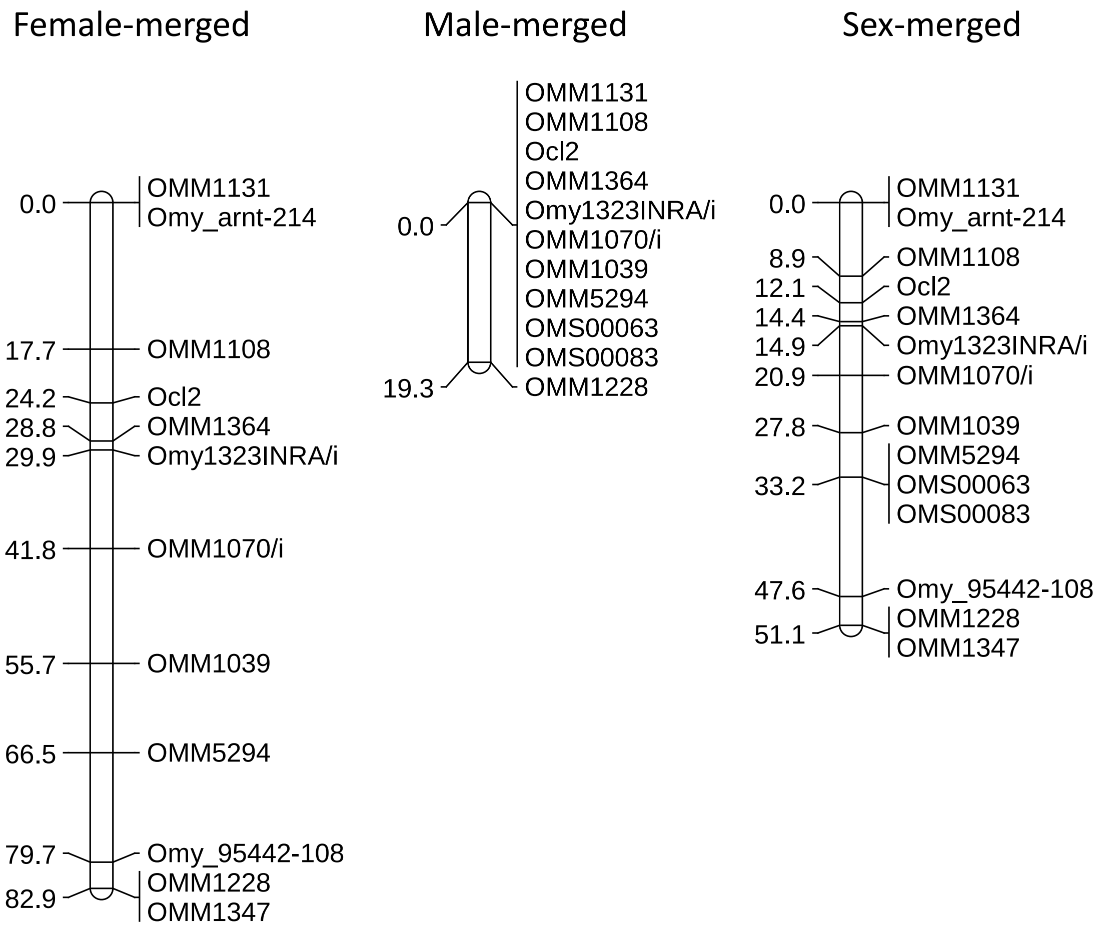

RYHyb03 (Omy3, RT31)

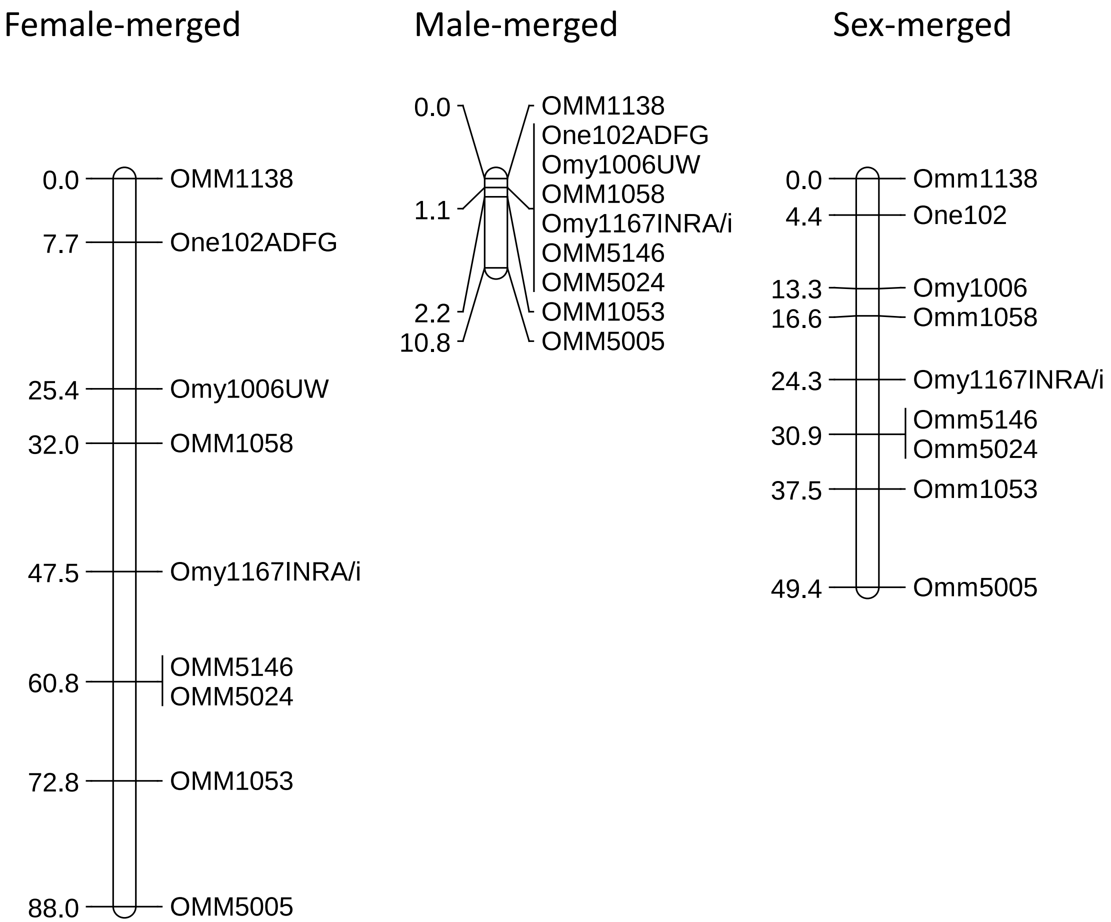

RYHyb04 (Omy4, RT24)

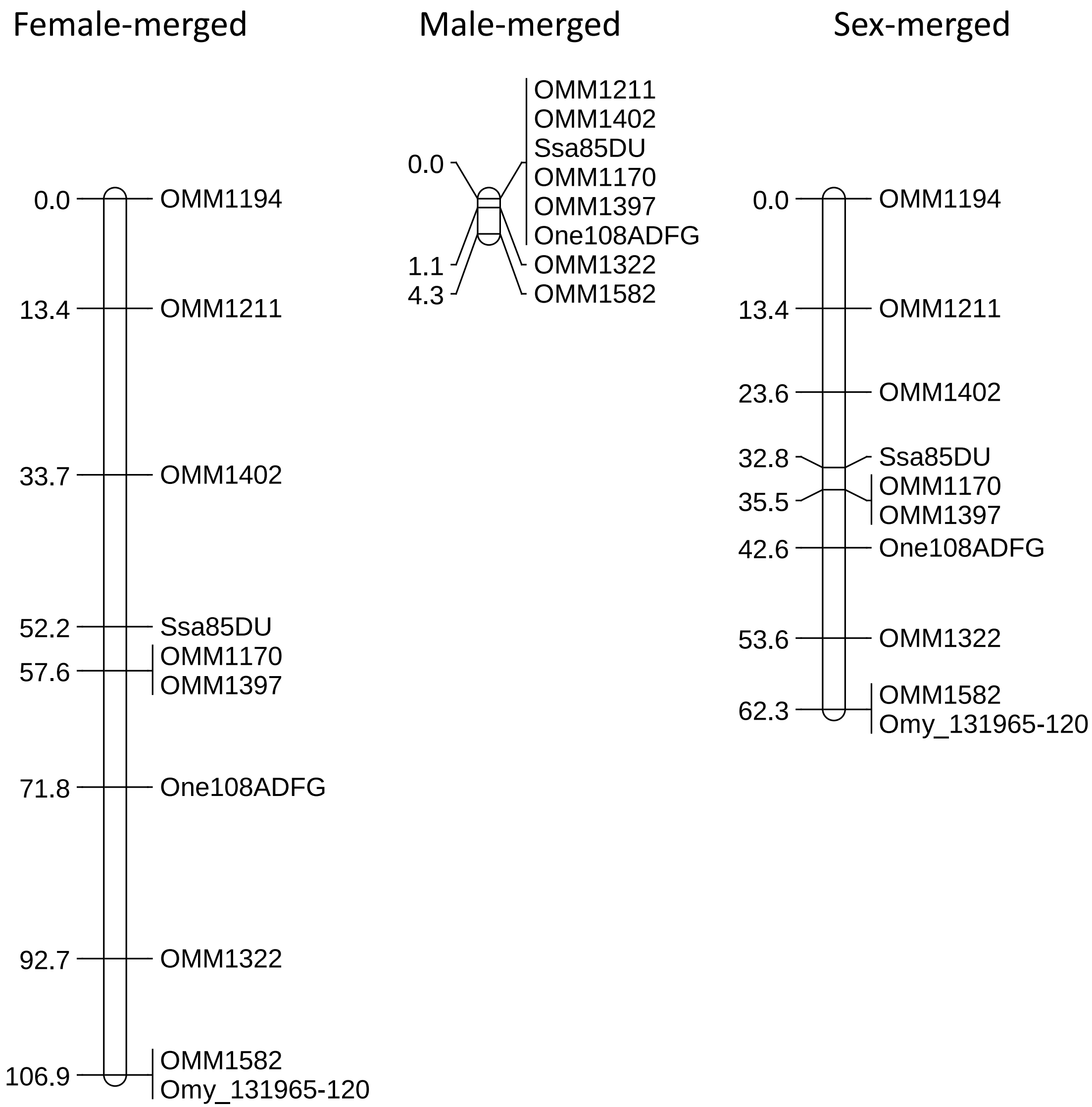

RYHyb05 (Omy5, RT8)

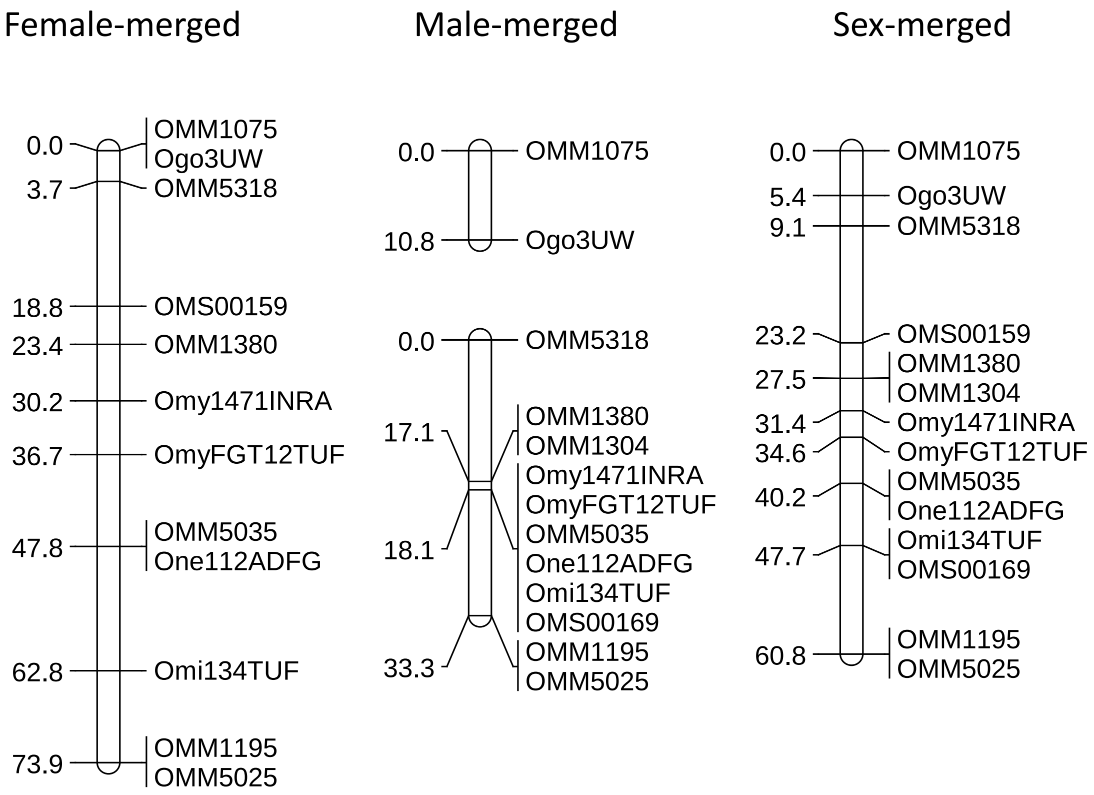

RYHyb06 (Omy6, RT10)

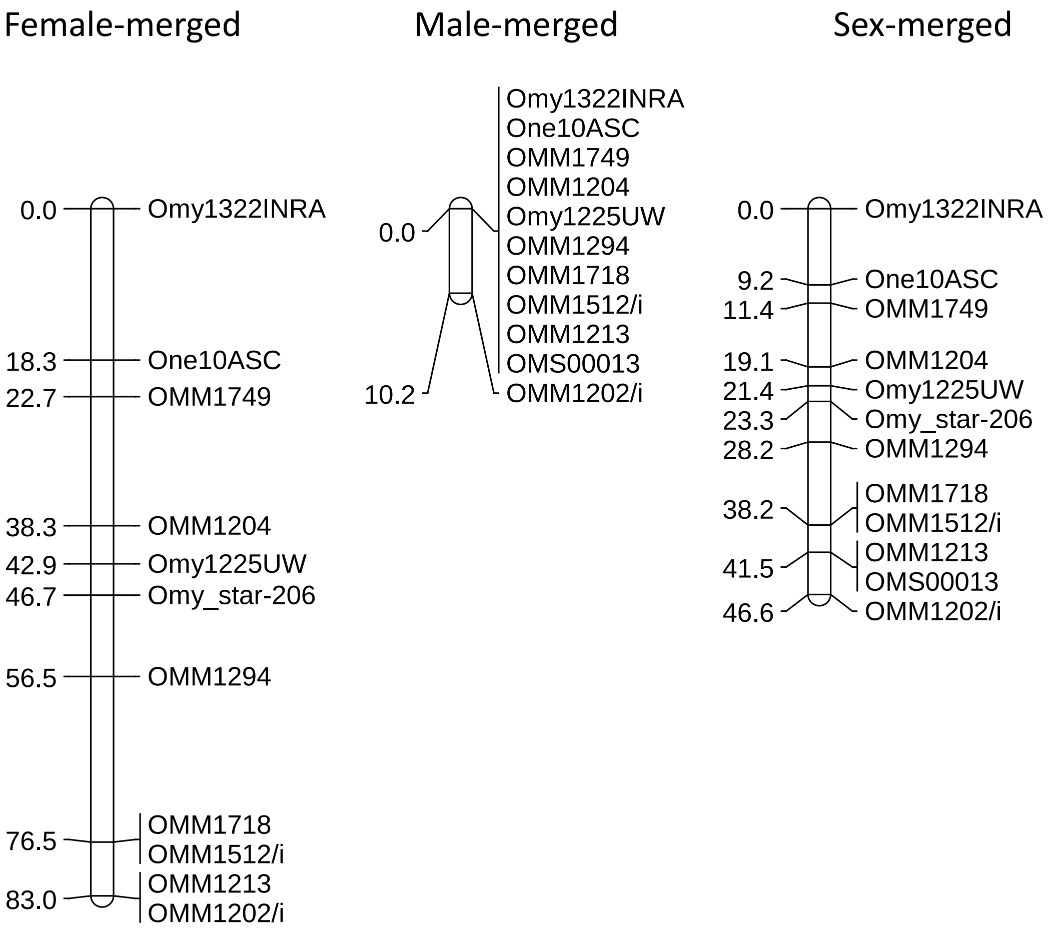

RYHyb07 (Omy7, RT12)

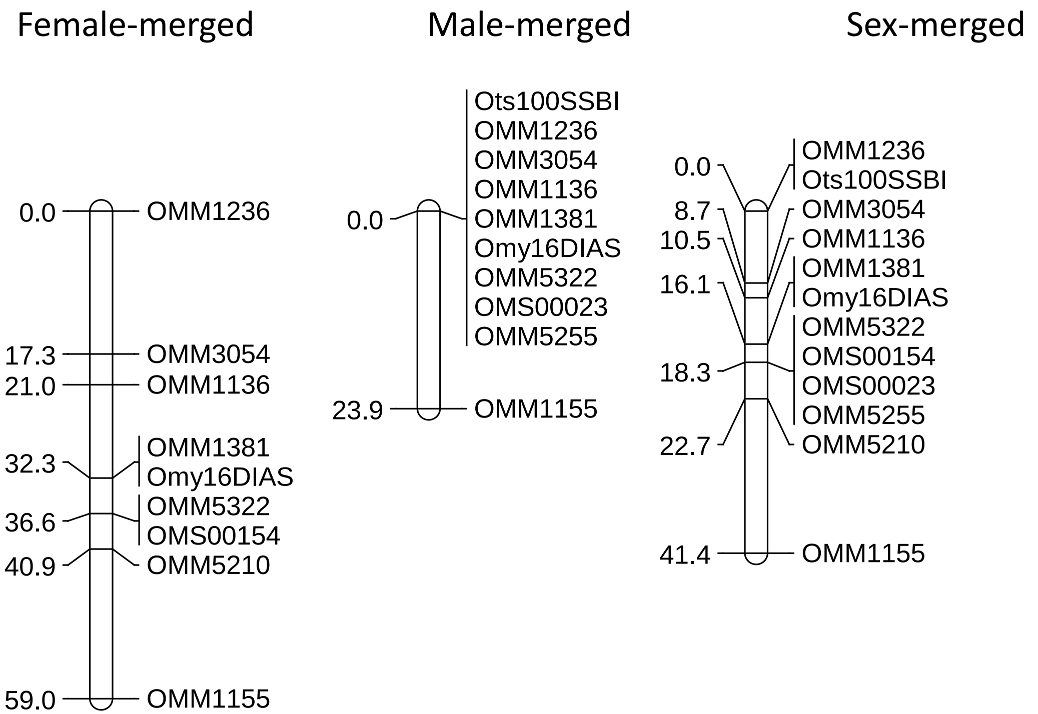

RYHyb08 (Omy8, RT23)

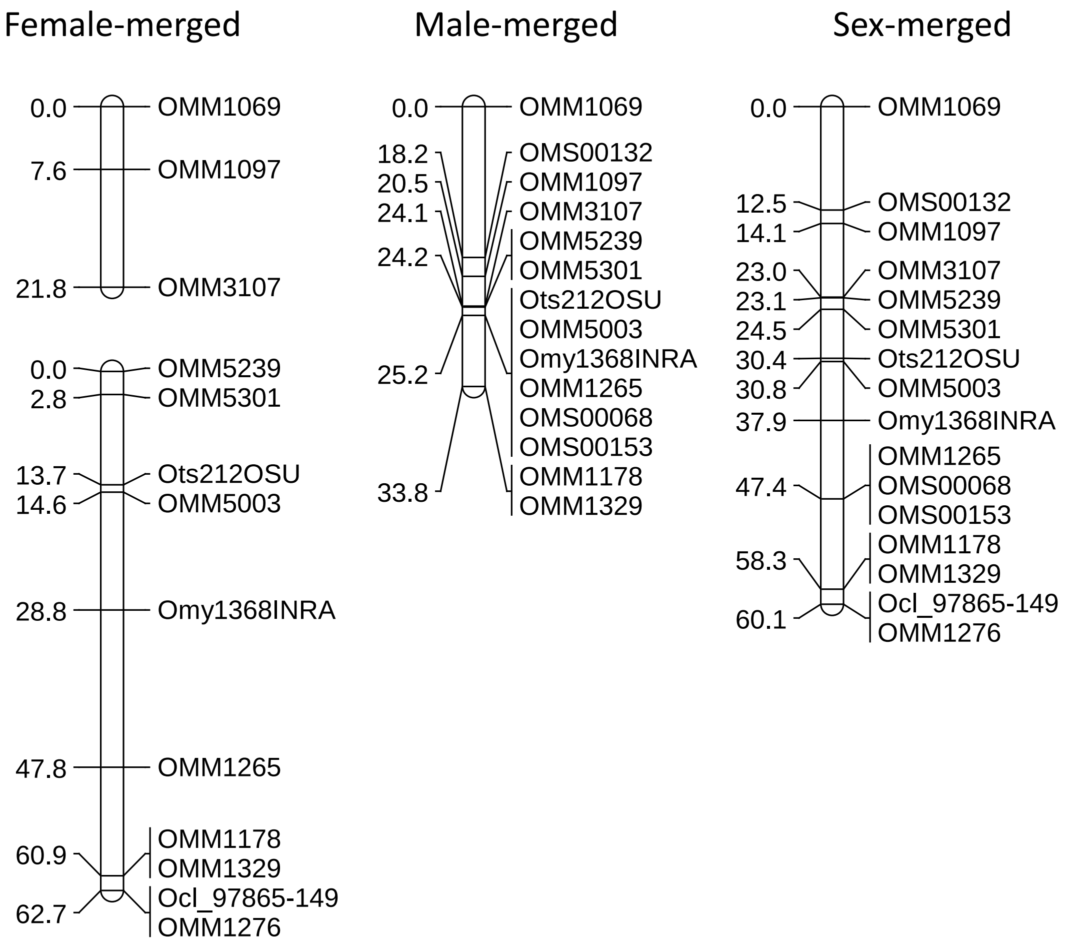

RYHyb09 (Omy9, RT21)

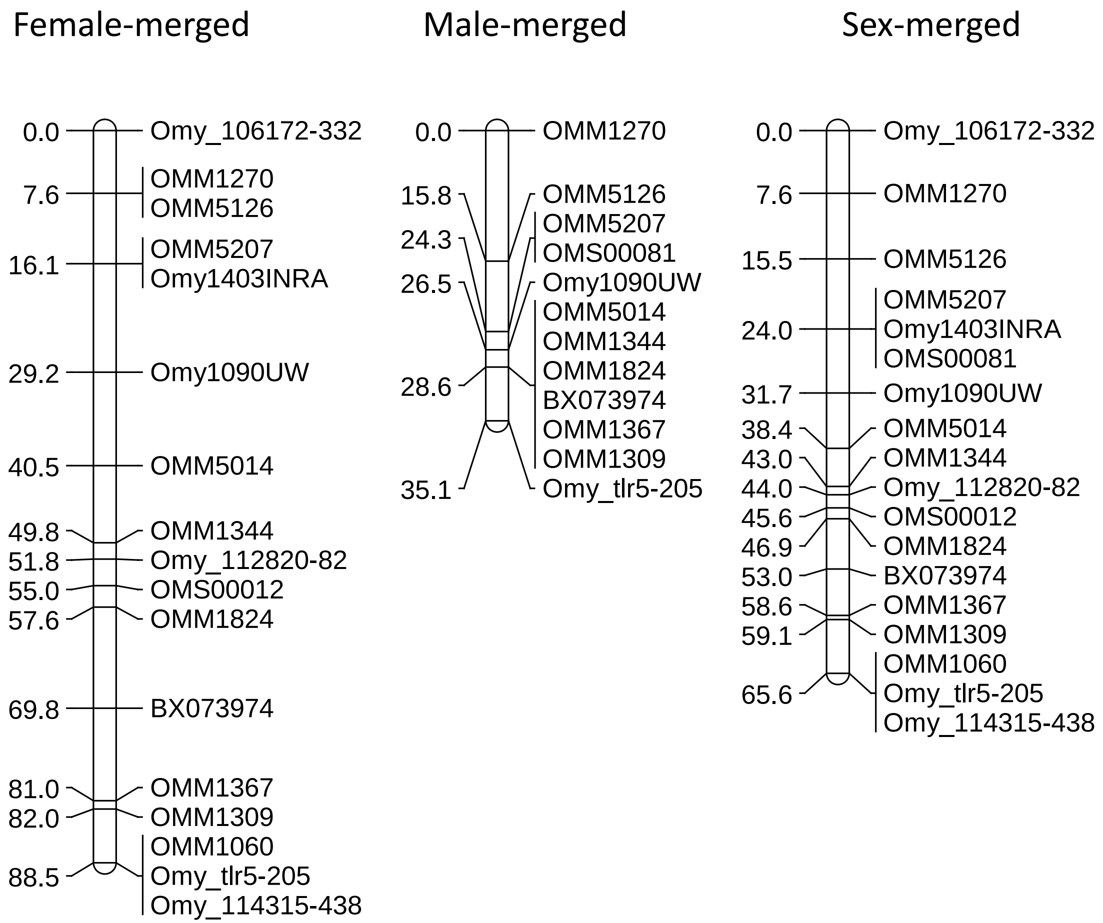

RYHyb10 (Omy10, RT20)

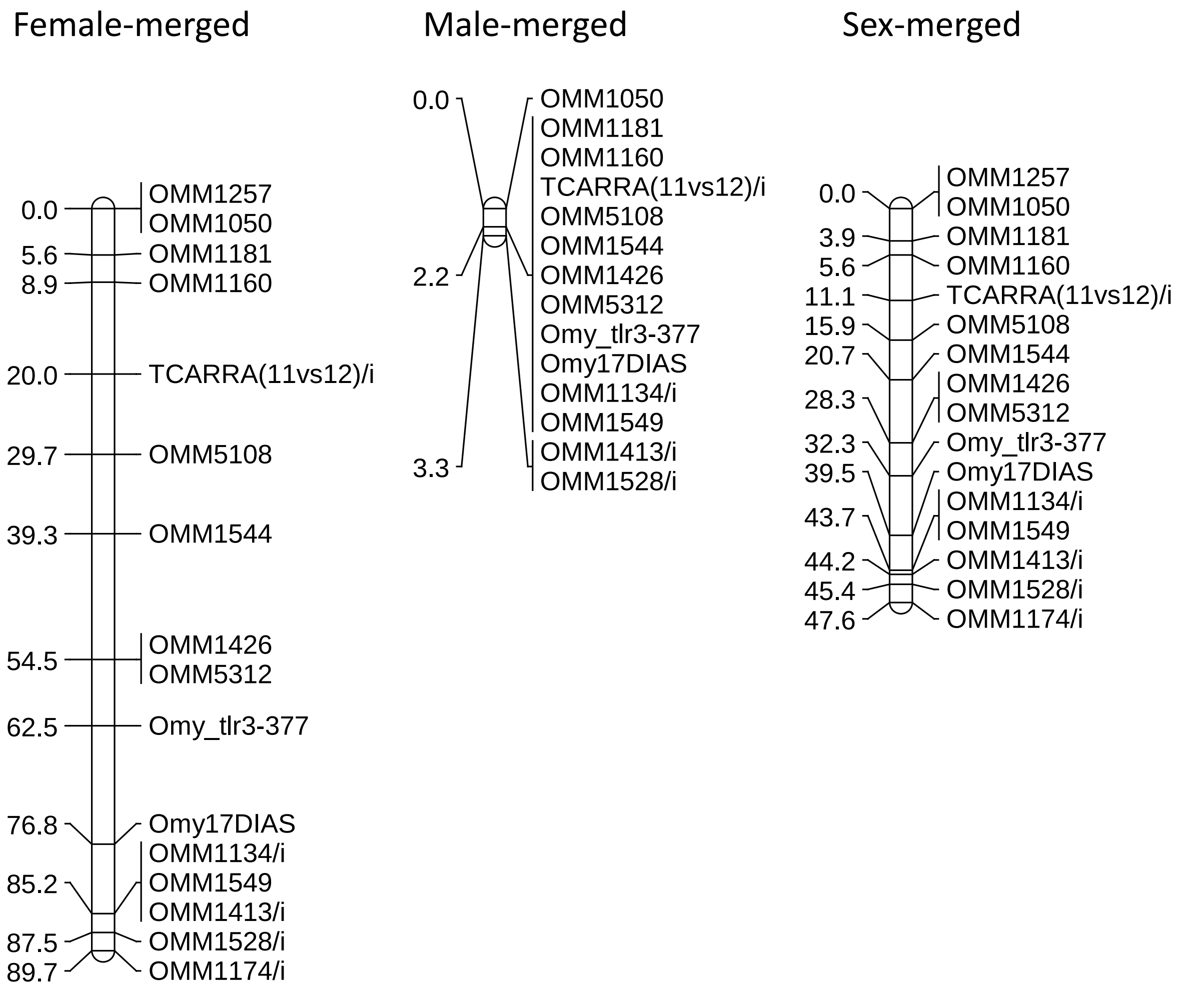

RYHyb11 (Omy11, RT19)

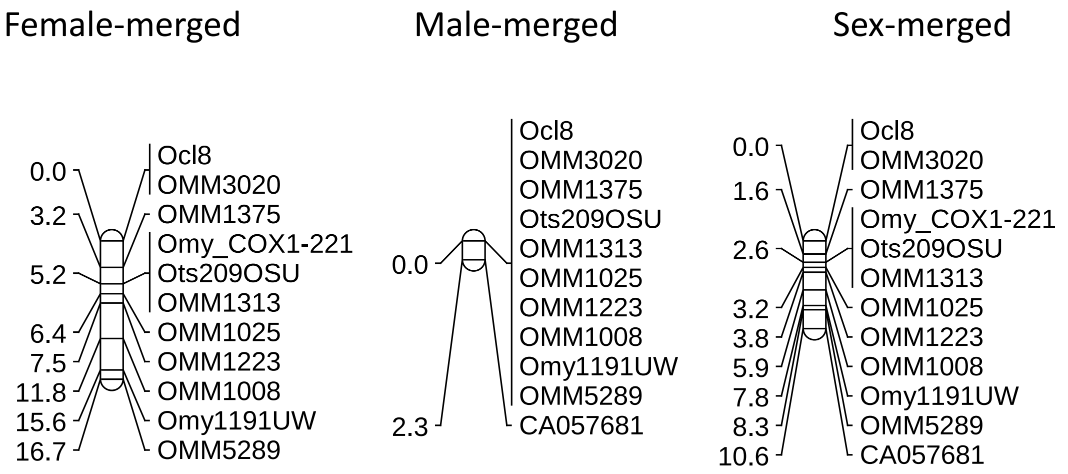

RYHyb12 (Omy12, RT9)

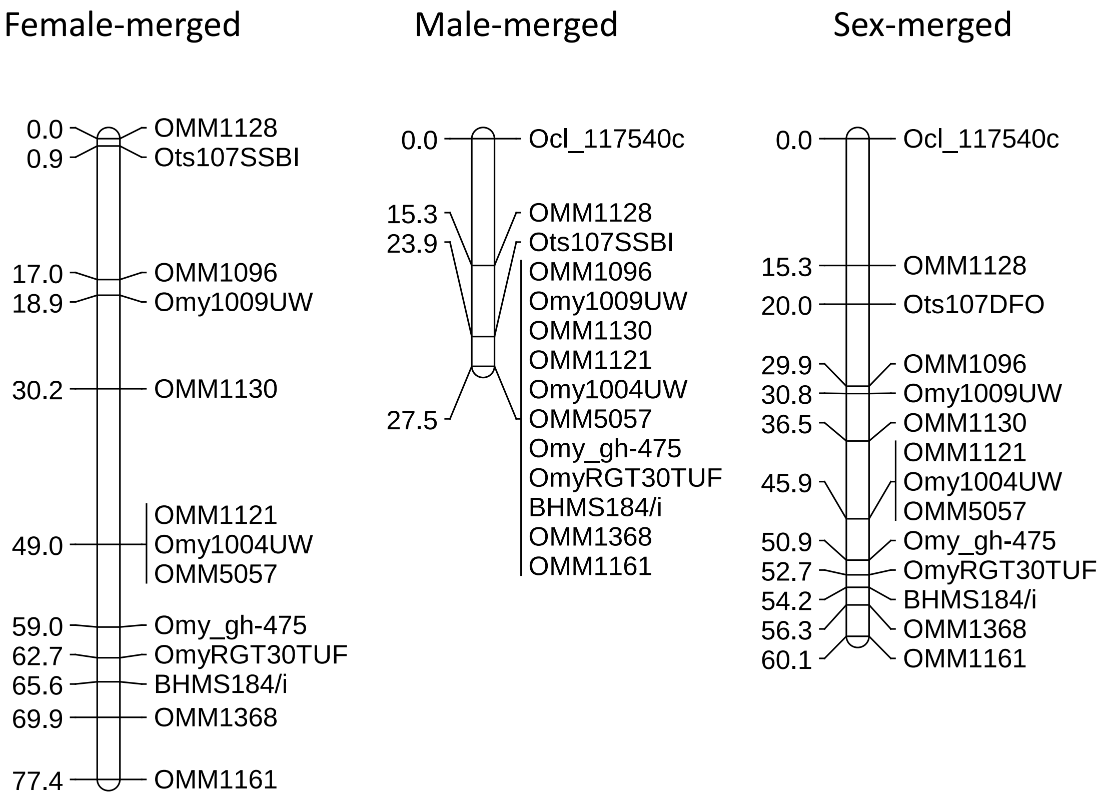

RYHyb13 (Omy13, RT2)

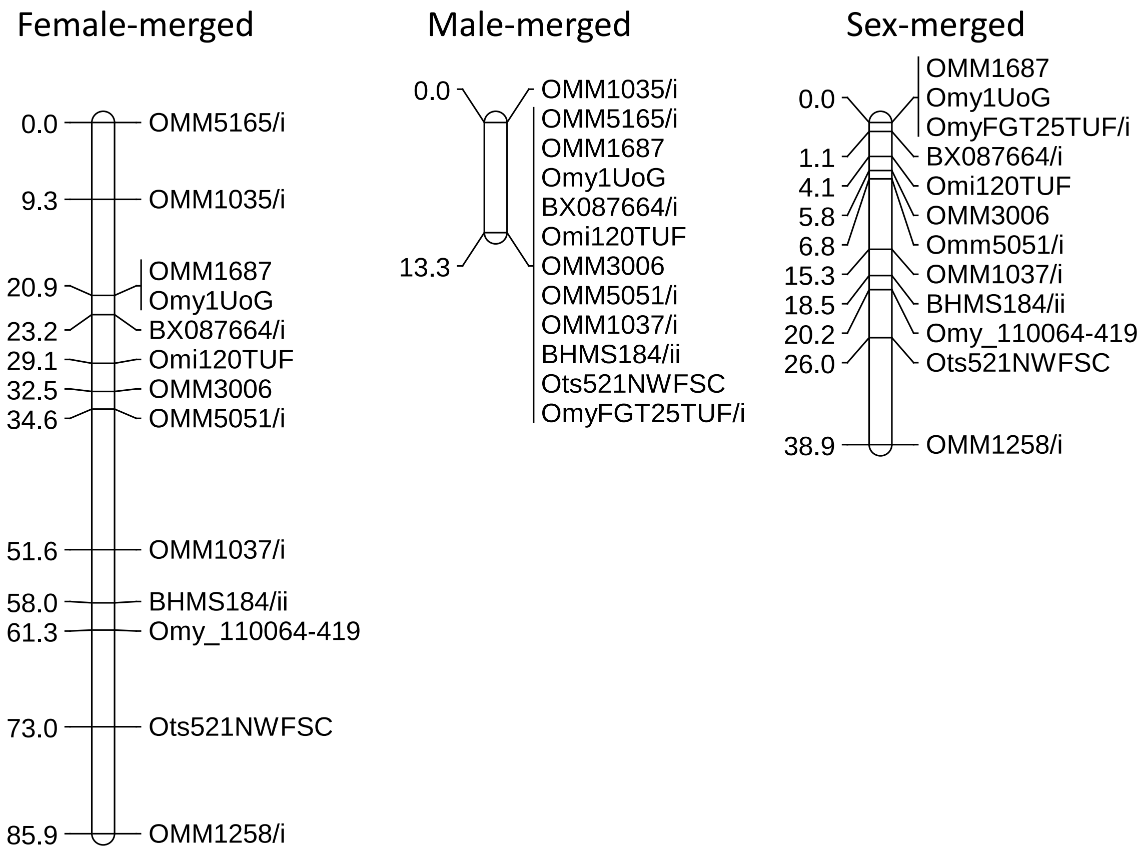

RYHyb14 (Omy14, RT3)

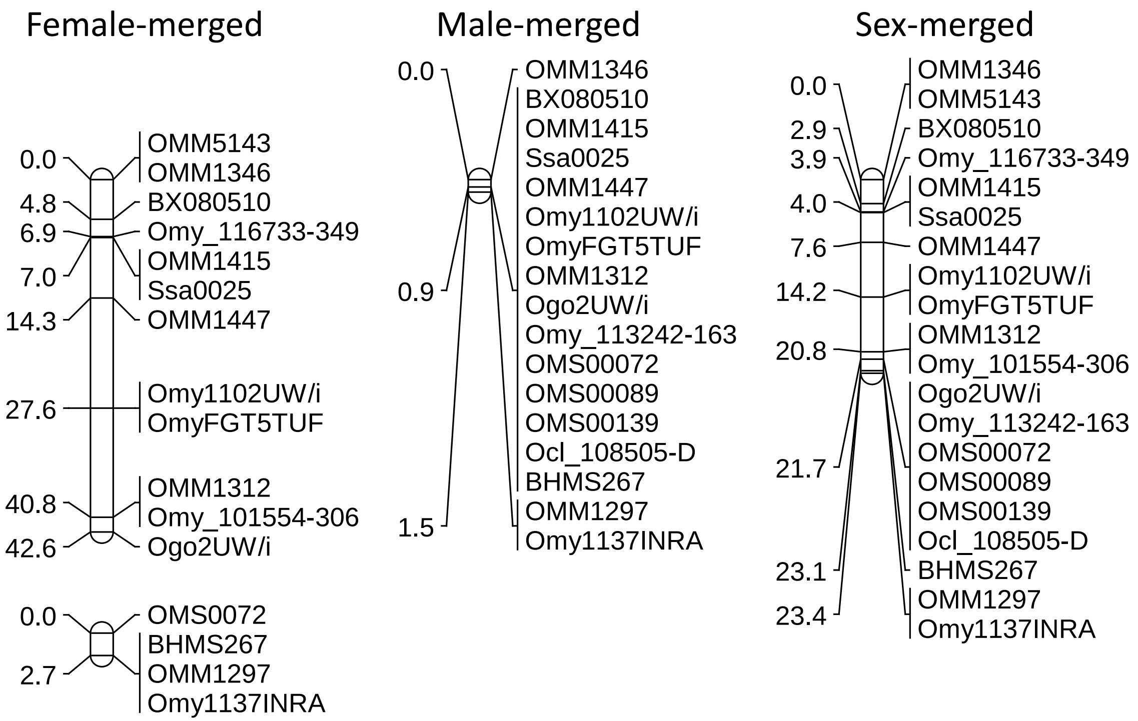

RYHyb15 (Omy15, RT7)

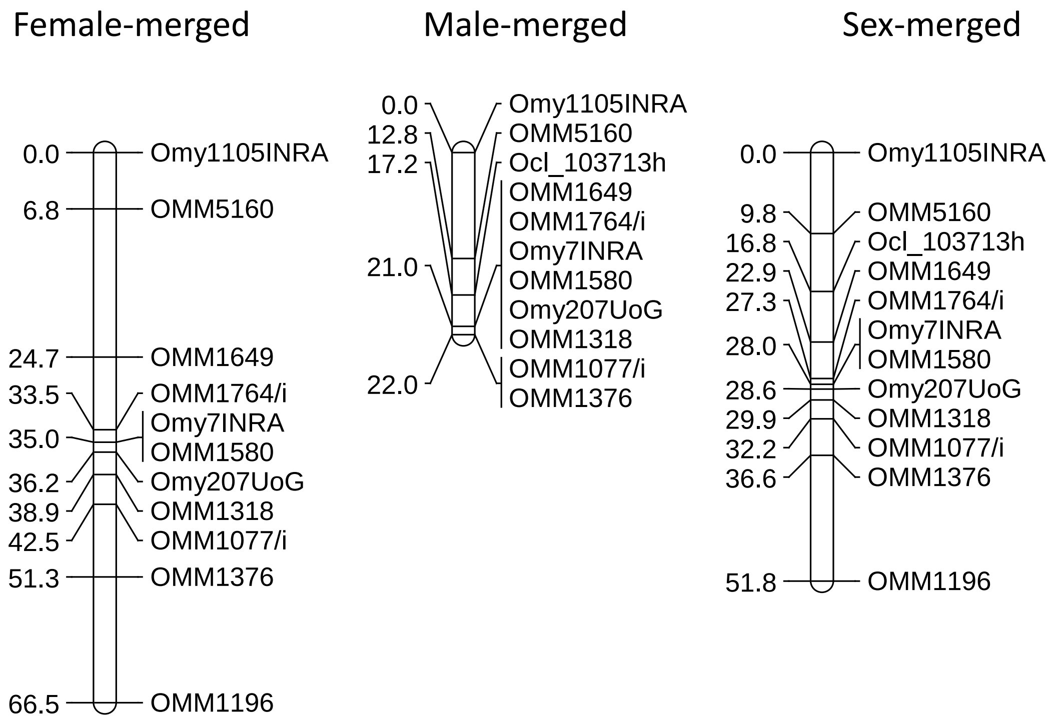

RYHyb16 (Omy16, RT22)

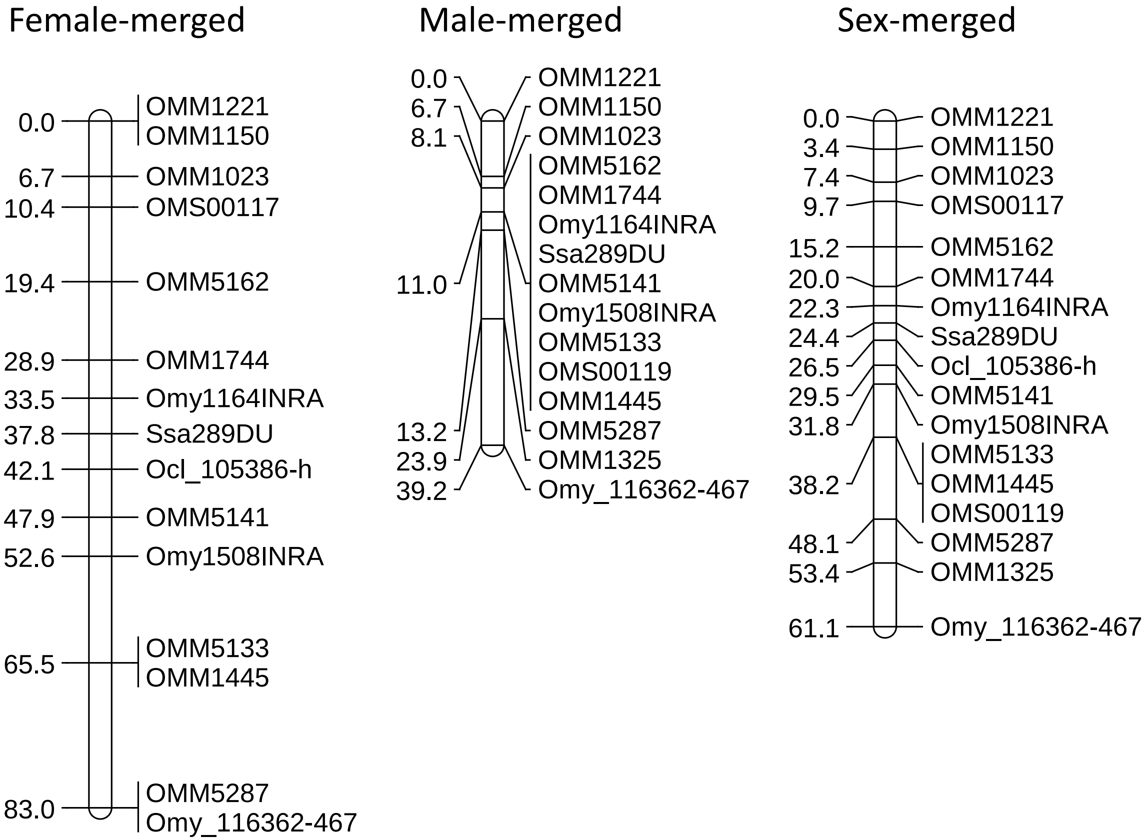

RYHyb17 (Omy17, RT29)

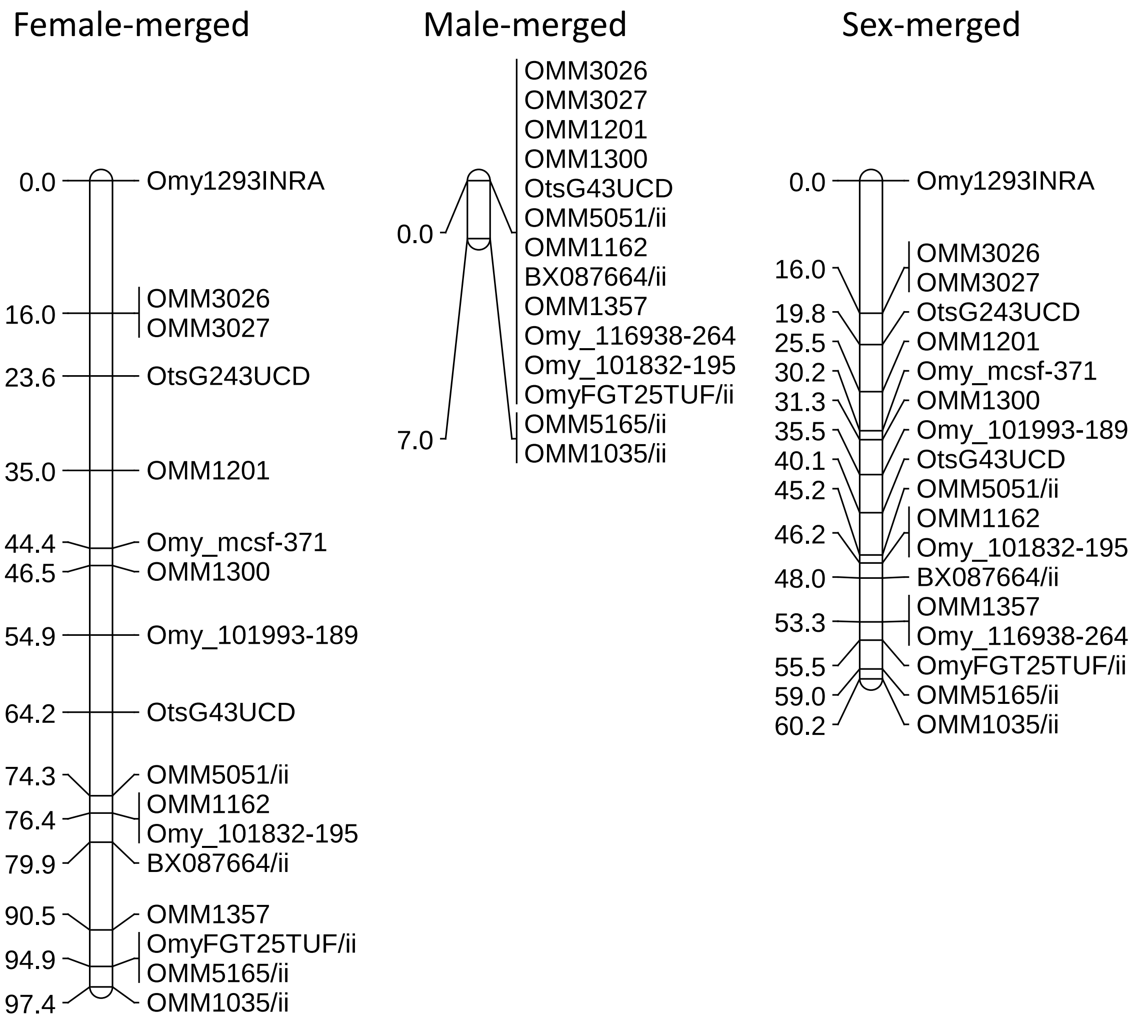

RYHyb18 (Omy18, RT16)

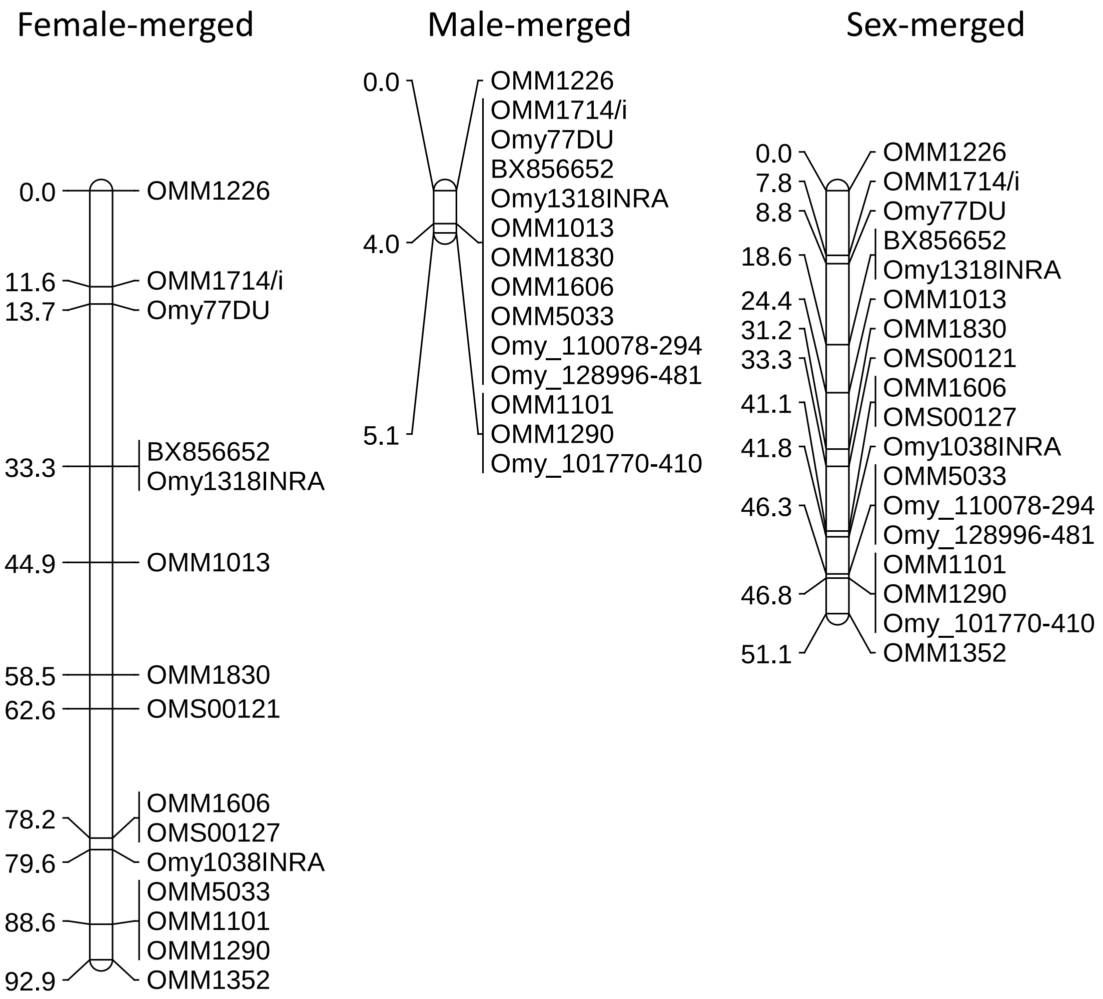

RYHyb19 (Omy19, RT14)

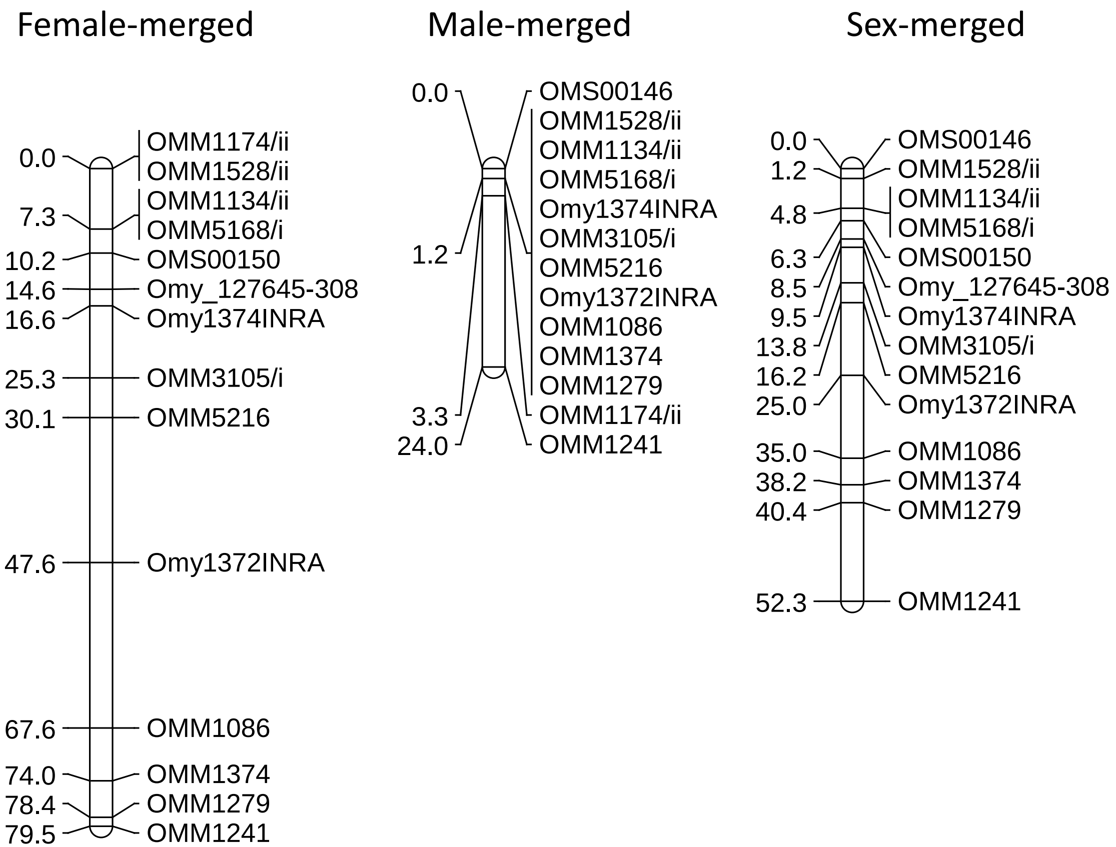

RYHyb20 (Omy28_Omy20, RT13_RT17)

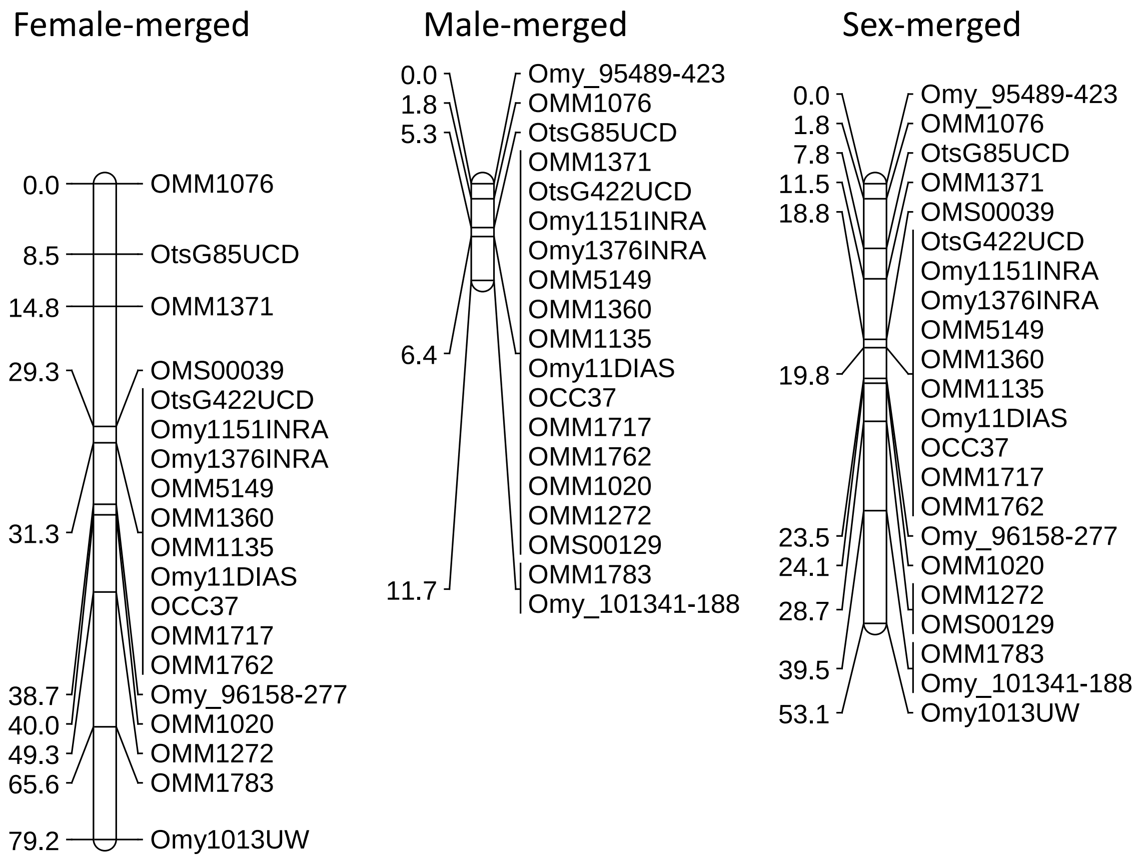

RYHyb21 (Omy21, RT15)

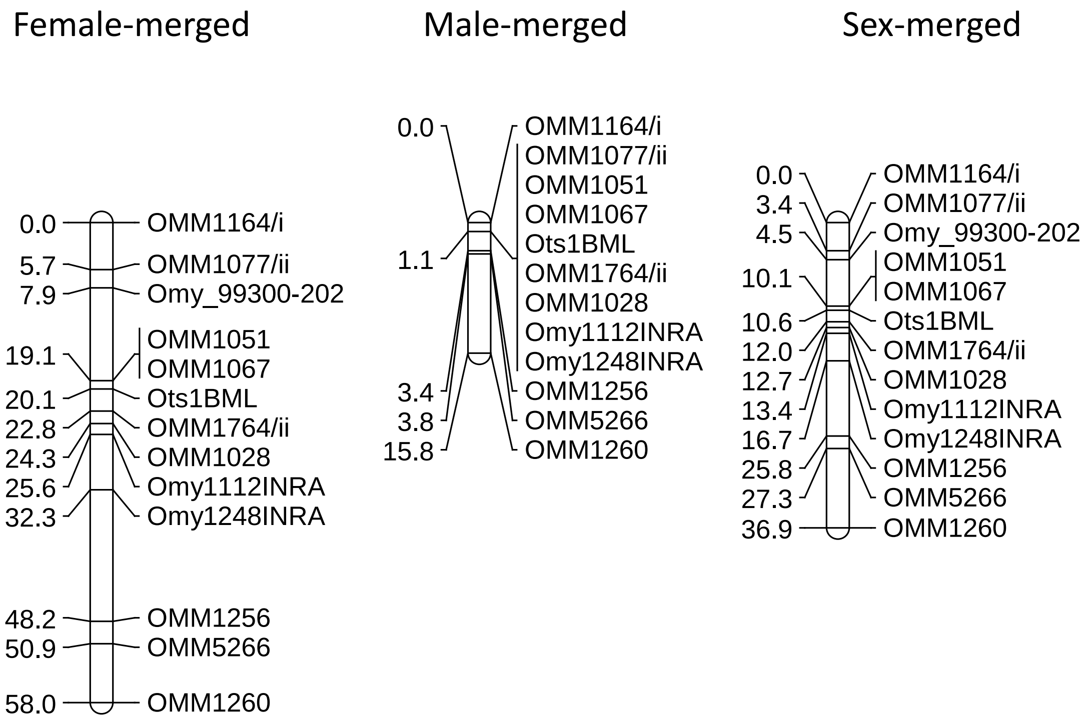

RYHyb22 (Omy22, RT5)

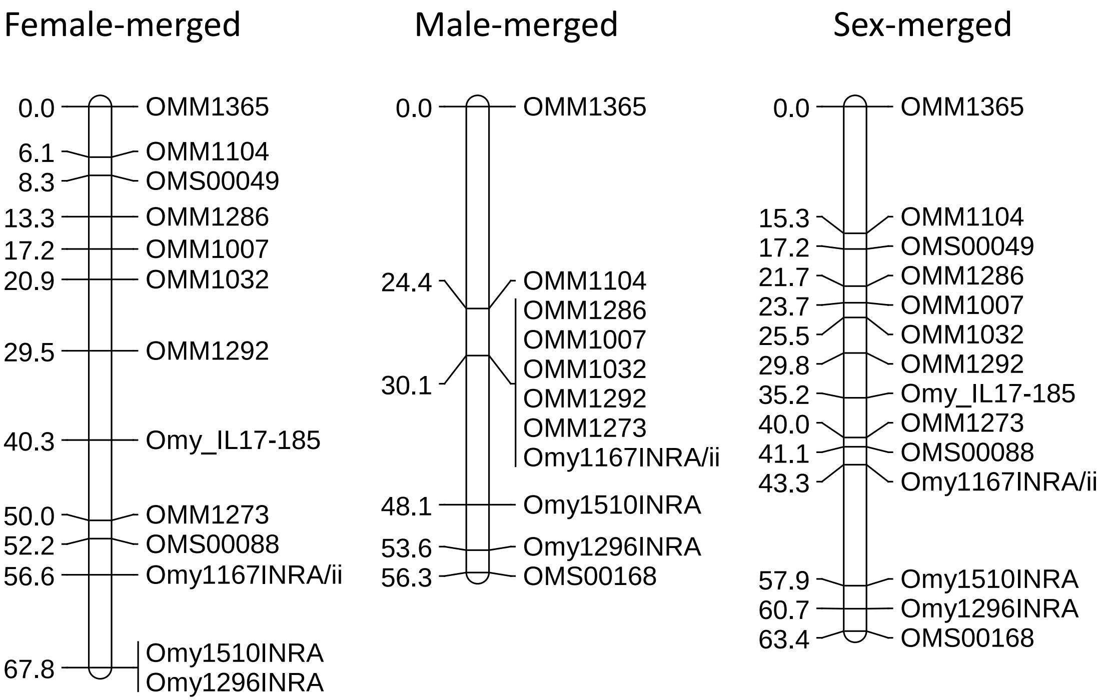

RYHyb23 (Omy23, RT30)

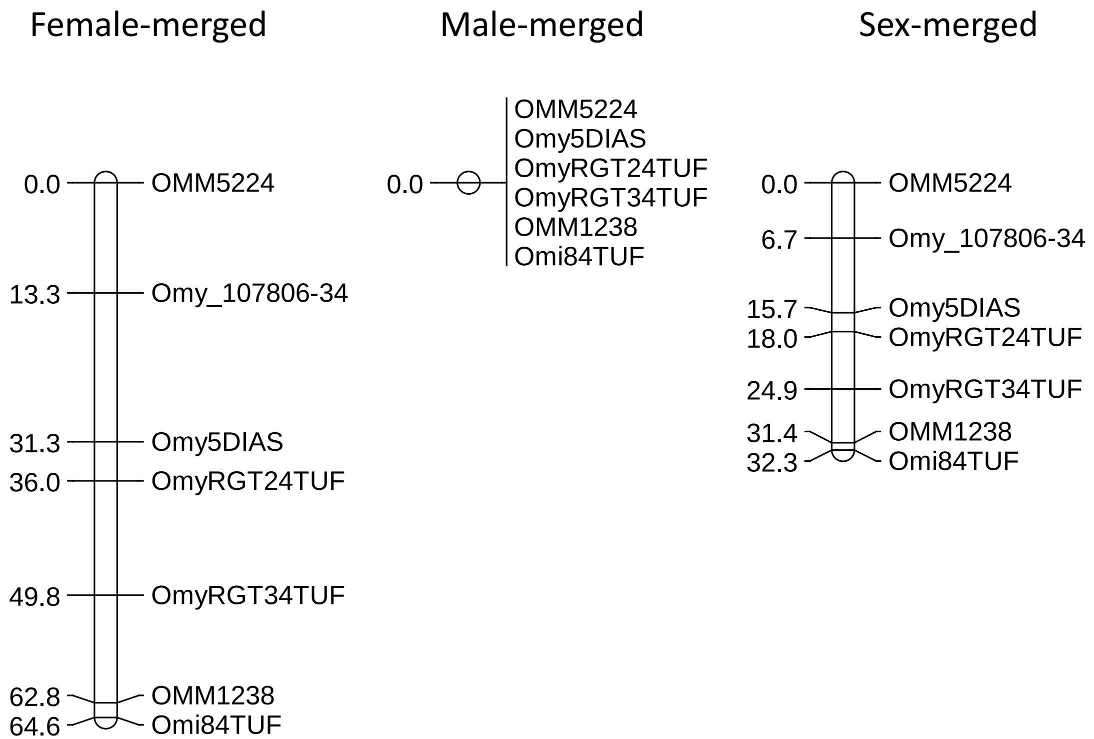

RYHyb24 (Omy24, RT26)

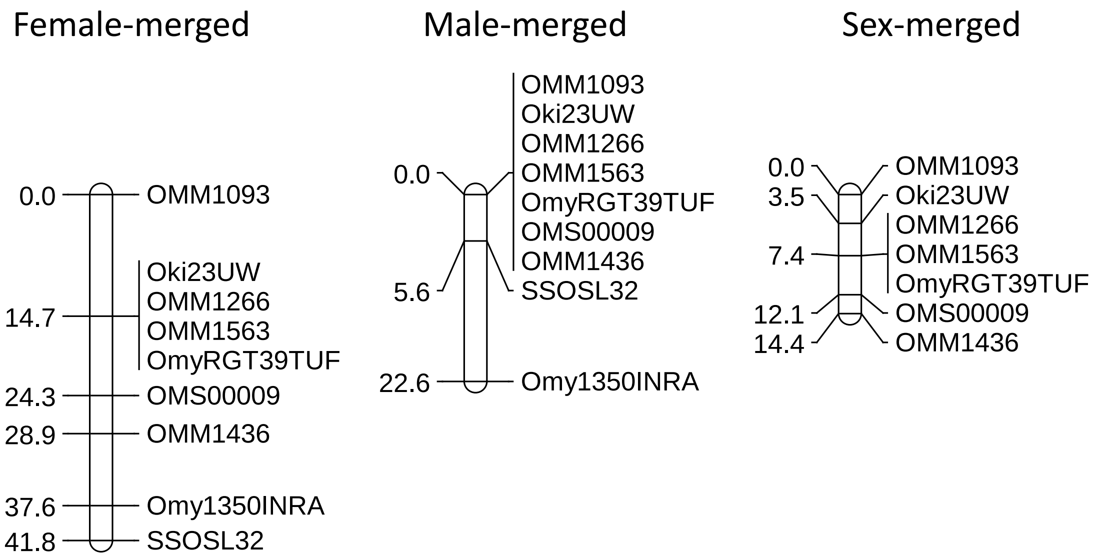

RYHyb25 (Omy25,RT4) and RYHyb29 (Omy29, RT25) polymorphism

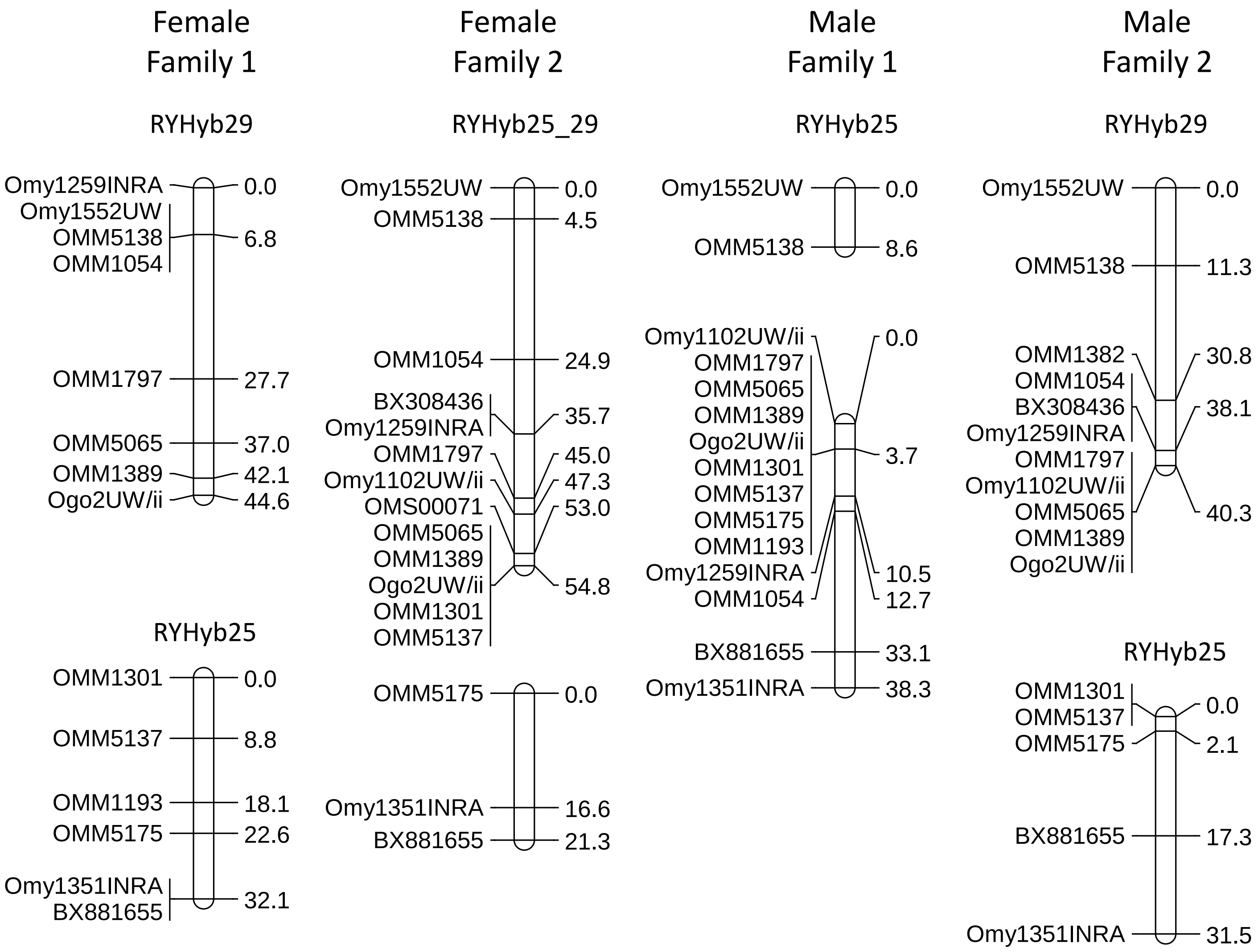

RYHyb26 (Omy26, RT18)

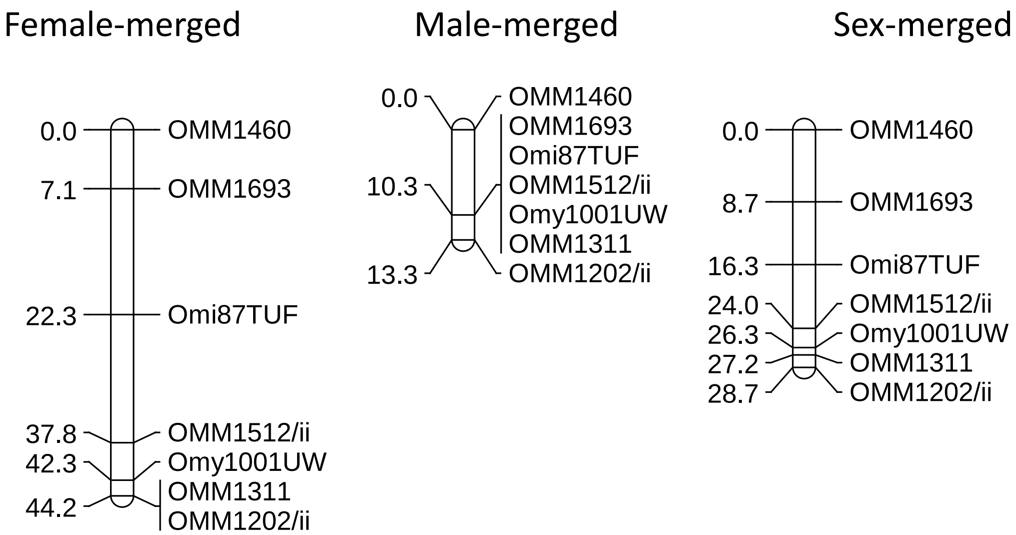

RYHyb27 (Omy27, RT11)

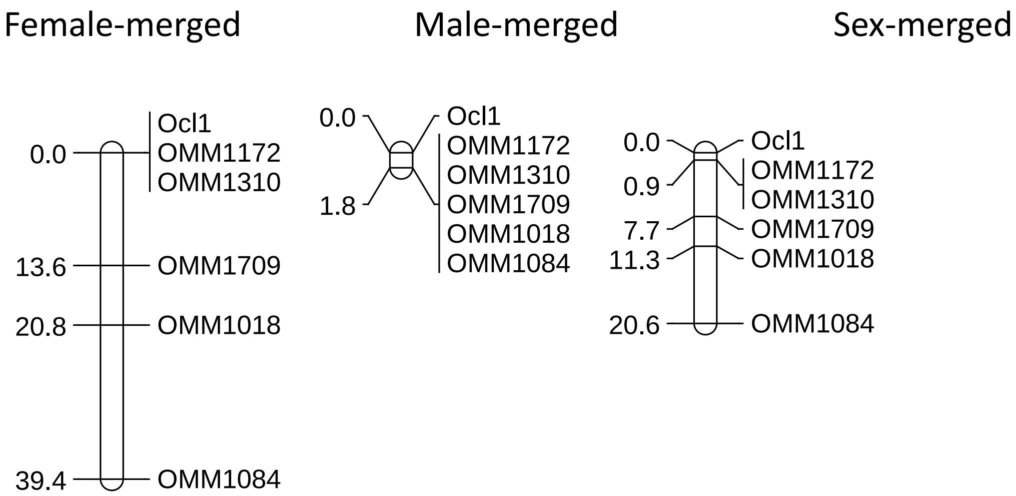

RYHyb29 (OmySex, RT1)

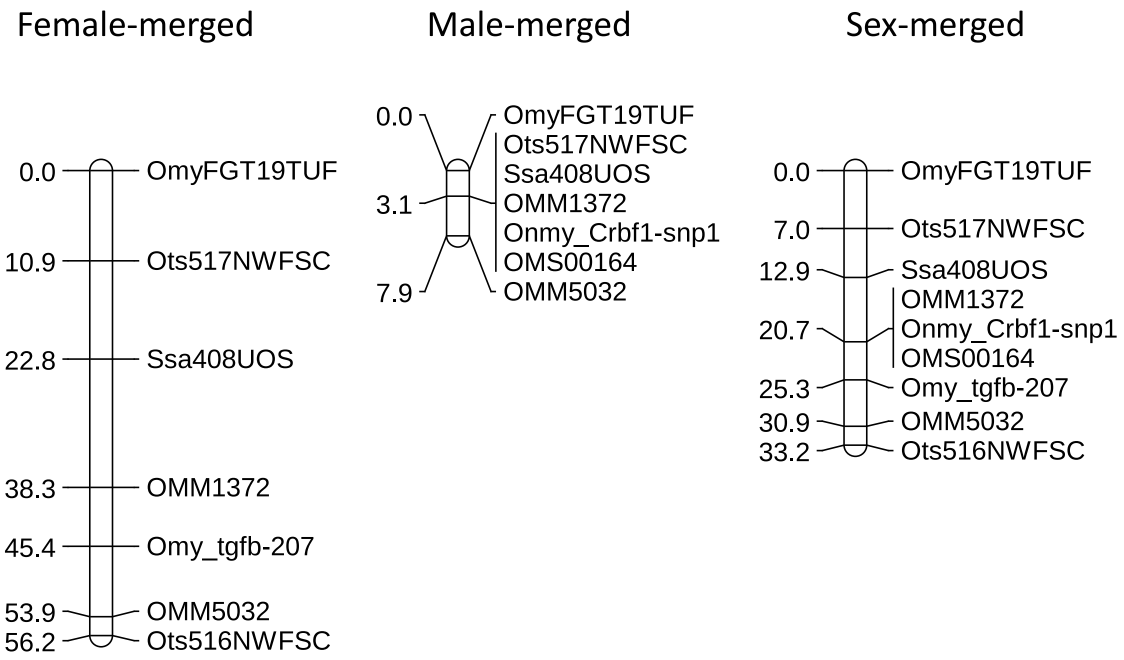

Supplement: Additional file 3 — This PDF file includes figures representing female-, male-, and sex-merged F1 hybrid linkage maps. [file 1471-2164-14-570-S3.pdf]
