# Supplementary material for: Chromosome rearrangements, recombination suppression, and limited segregation distortion in hybrids between Yellowstone cutthroat trout (Oncorhynchus clarkii bouvieri) and rainbow trout (O. mykiss)
Source: BMC Genomics. 2013 Aug 22;14:570. doi: 10.1186/1471-2164-14-570 (PMC3765842; doi:10.1186/1471-2164-14-570)
Supplement: Additional file 4 — This PDF file includes figures that compare map distances, in centiMorgans, across the same markers in the female-merged F1 hybrid linkage map (X-axis) and the female consensus rainbow trout map (Y-axis) for each linkage group. [file 1471-2164-14-570-S4.pdf]

Additional file 4. Comparison of map distances in centiMorgans (cM) across the same markers in the female-merged F_1 hybrid linkage map (X-axis) and the female consensus rainbow trout (RBT) map (Y-axis). Black diamonds indicate mapped markers (*p* to *q* orientation) and brackets indicate putative centromere locations for metacentric linkage groups identified in Guyomard et al. (2006). For acrocentric linkage groups, the marker closest to the centromere is plotted at zero. Significant differences in recombination (*P*-values) between the female-merged F_1 and consensus RBT female maps across linkage groups are indicated (ns, not significant).

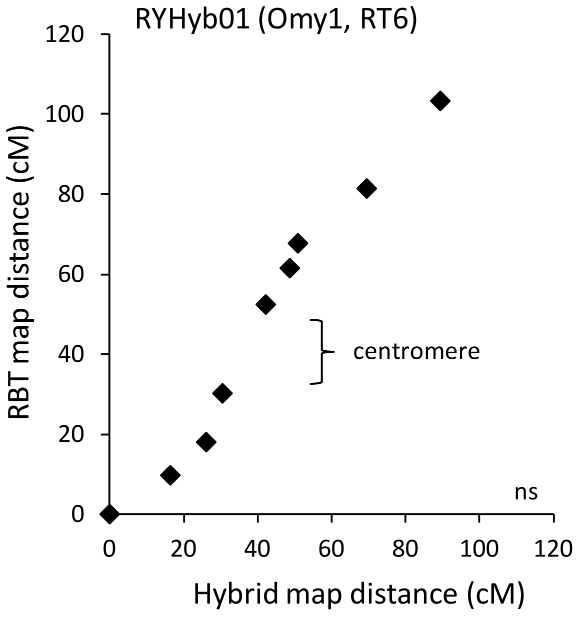
RYHyb01 (Omy1, RT6)
RBT map distance (cM)
Hybrid map distance (cM)
centromere
ns
0
20
40
60
80
100
120

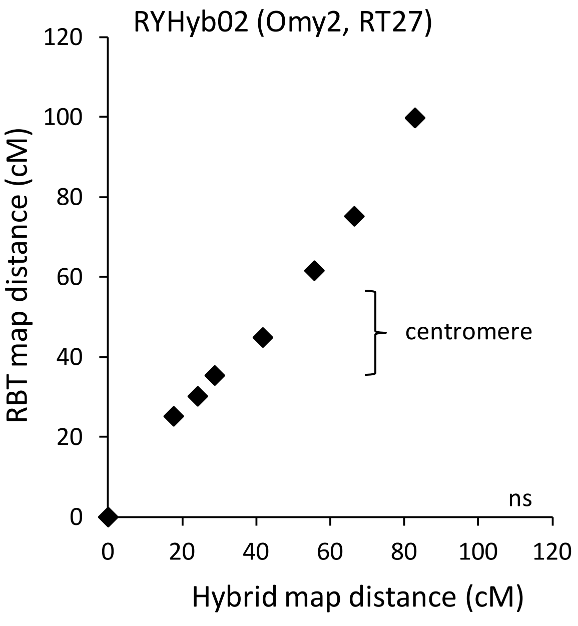
RYHyb02 (Omy2, RT27)
RBT map distance (cM)
Hybrid map distance (cM)
centromere
ns
0
20
40
60
80
100
120

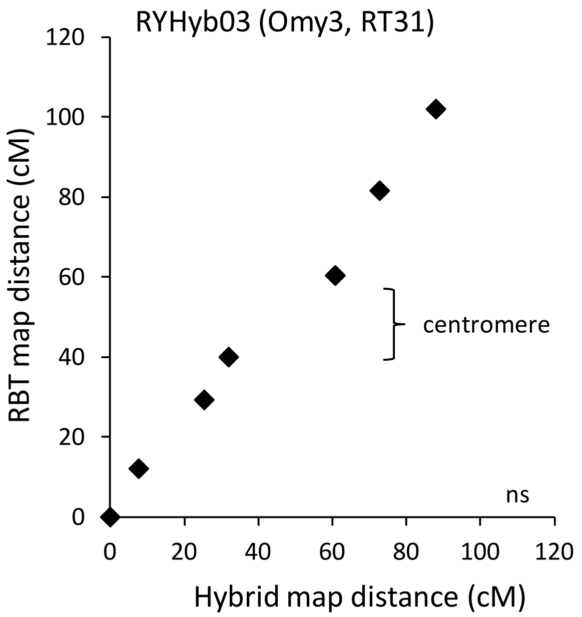
RYHyb03 (Omy3, RT31)
RBT map distance (cM)
Hybrid map distance (cM)
centromere
ns
0
20
40
60
80
100
120

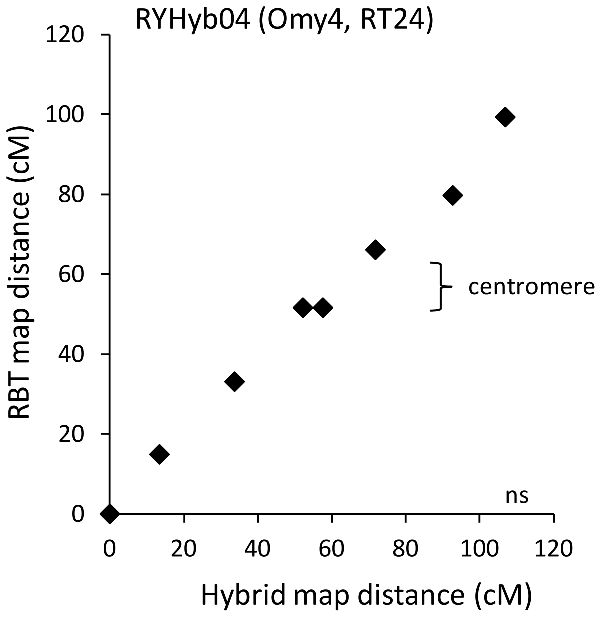
RYHyb04 (Omy4, RT24)
RBT map distance (cM)
Hybrid map distance (cM)
centromere
ns
0
20
40
60
80
100
120

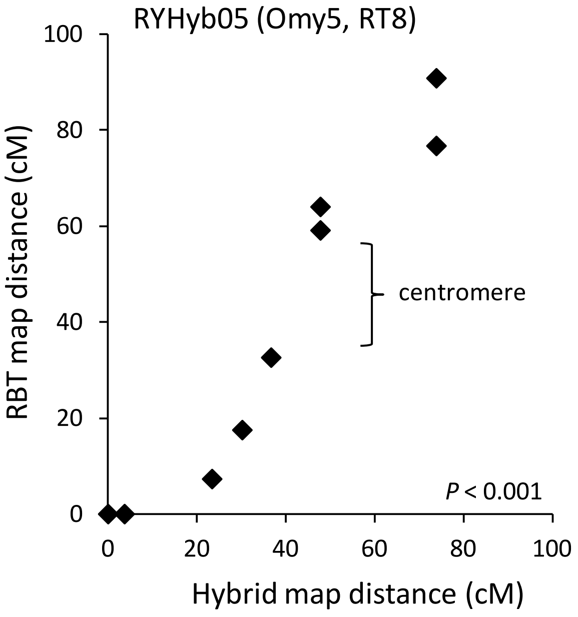
RYHyb05 (Omy5, RT8)
RBT map distance (cM)
Hybrid map distance (cM)
centromere
P < 0.001
0
20
40
60
80
100

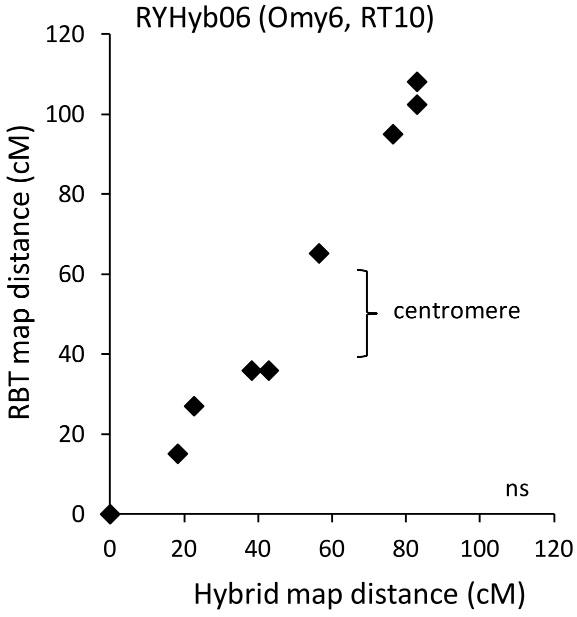
RYHyb06 (Omy6, RT10)
RBT map distance (cM)
Hybrid map distance (cM)
centromere
ns
0
20
40
60
80
100
120

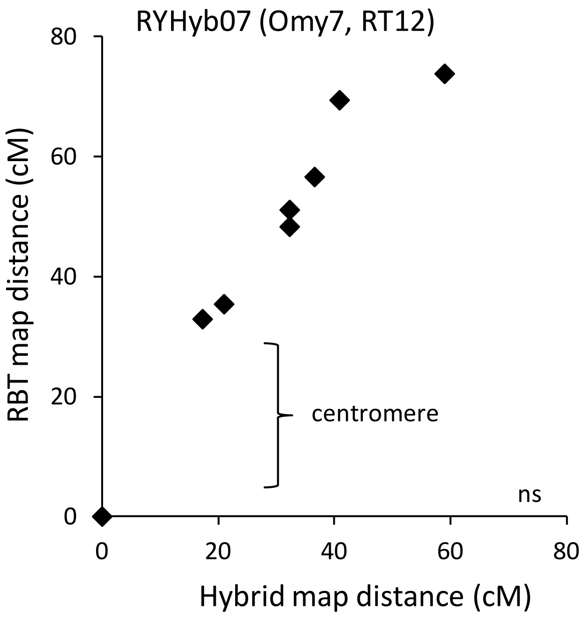
RYHyb07 (Omy7, RT12)
RBT map distance (cM)
Hybrid map distance (cM)
centromere
ns
0
20
40
60
80

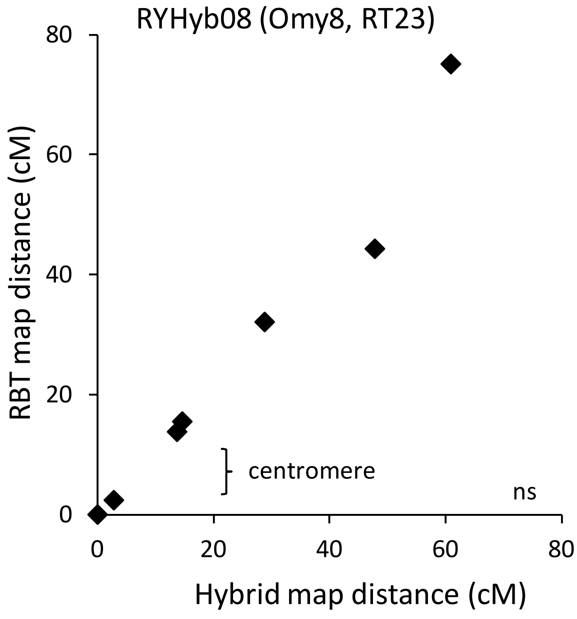
RYHyb08 (Omy8, RT23)
RBT map distance (cM)
Hybrid map distance (cM)
centromere
ns
0
20
40
60
80

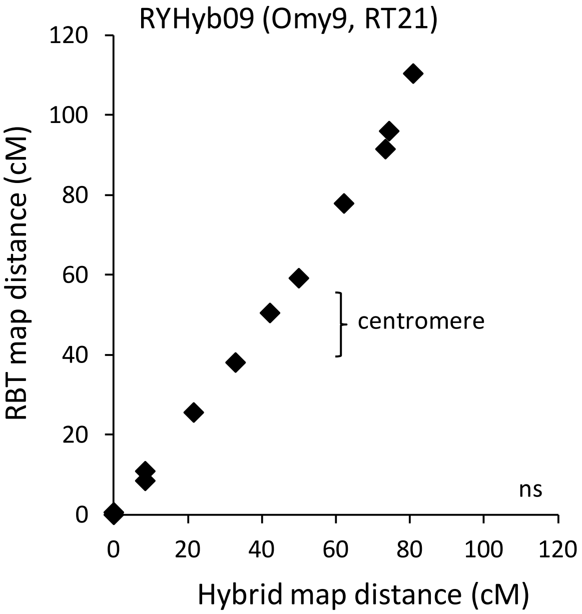
RYHyb09 (Omy9, RT21)
RBT map distance (cM)
Hybrid map distance (cM)
centromere
ns
0
20
40
60
80
100
120

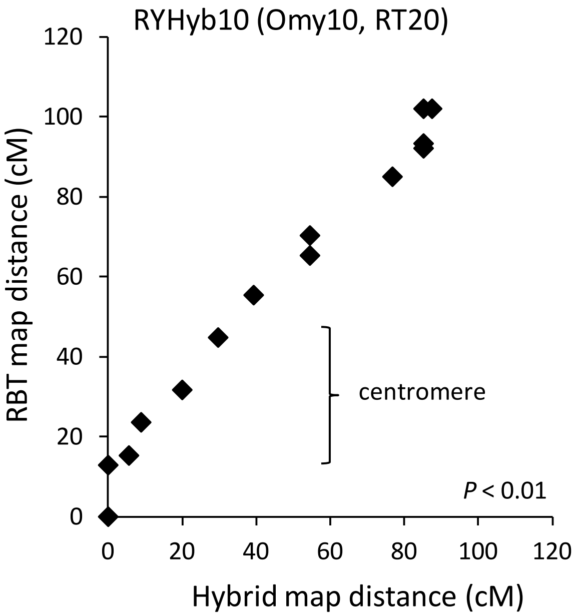
RYHyb10 (Omy10, RT20)
RBT map distance (cM)
Hybrid map distance (cM)
centromere
P < 0.01
0
20
40
60
80
100
120

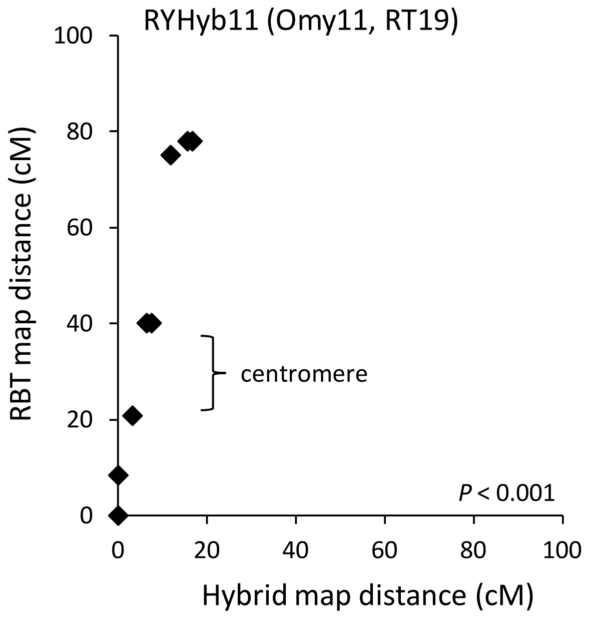
RYHyb11 (Omy11, RT19)
RBT map distance (cM)
Hybrid map distance (cM)
centromere
P < 0.001
0
20
40
60
80
100

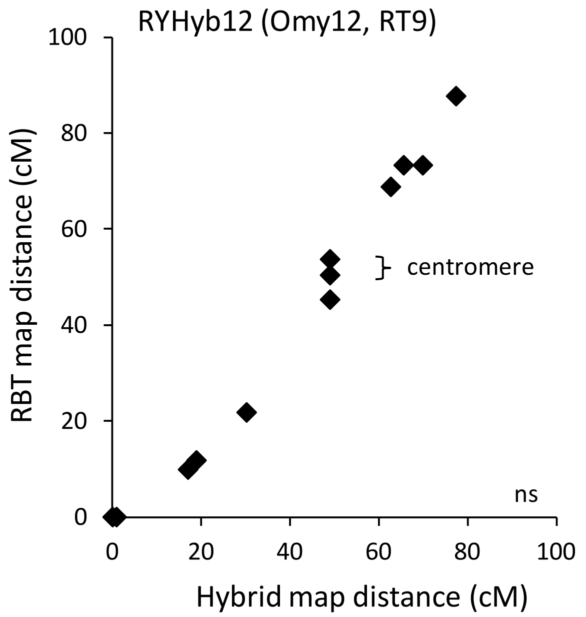
RYHyb12 (Omy12, RT9)
RBT map distance (cM)
Hybrid map distance (cM)
centromere
ns
0
20
40
60
80
100

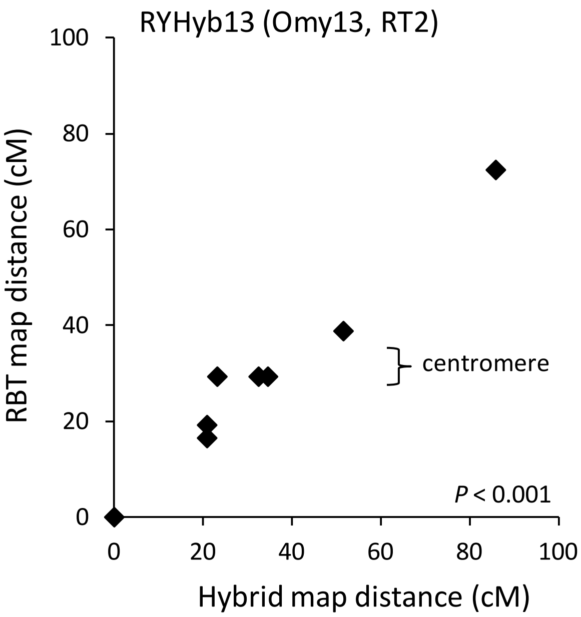

RYHyb13 (Omy13, RT2)
RBT map distance (cM)
Hybrid map distance (cM)
centromere
P < 0.001
0
20
40
60
80
100

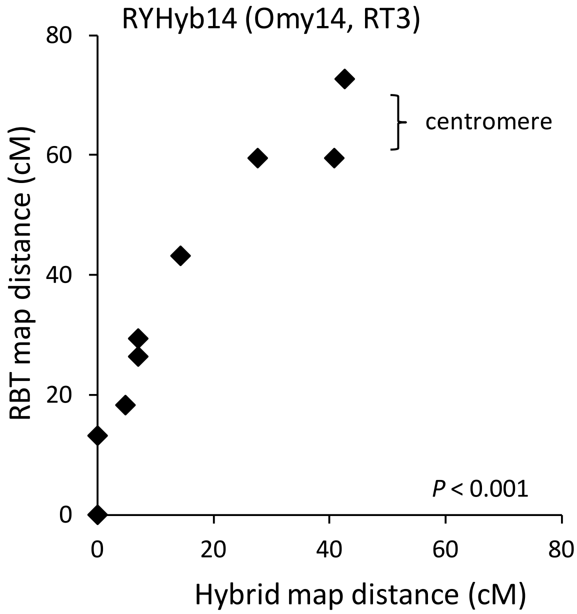

RYHyb14 (Omy14, RT3)
RBT map distance (cM)
Hybrid map distance (cM)
centromere
P < 0.001
0
20
40
60
80

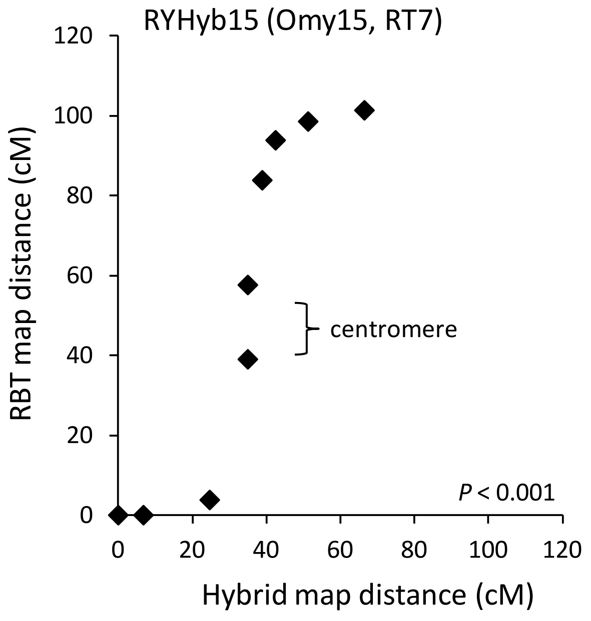
RYHyb15 (Omy15, RT7)
RBT map distance (cM)
Hybrid map distance (cM)
centromere
P < 0.001
0
20
40
60
80
100
120

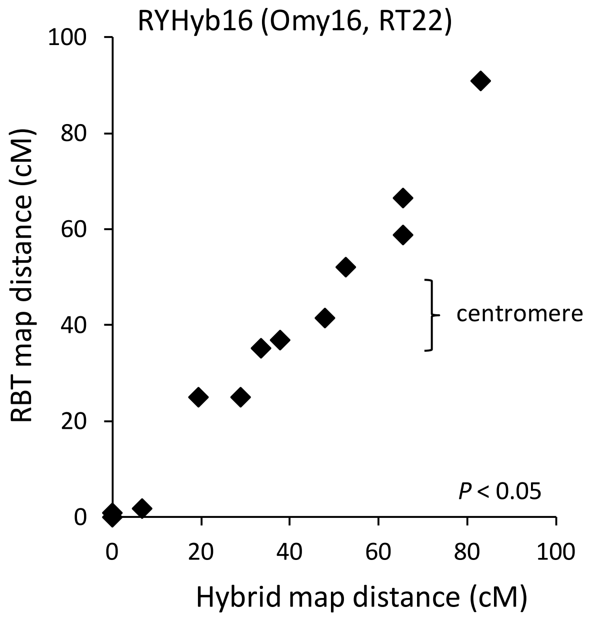
RYHyb16 (Omy16, RT22)
RBT map distance (cM)
Hybrid map distance (cM)
centromere
P < 0.05
0
20
40
60
80
100

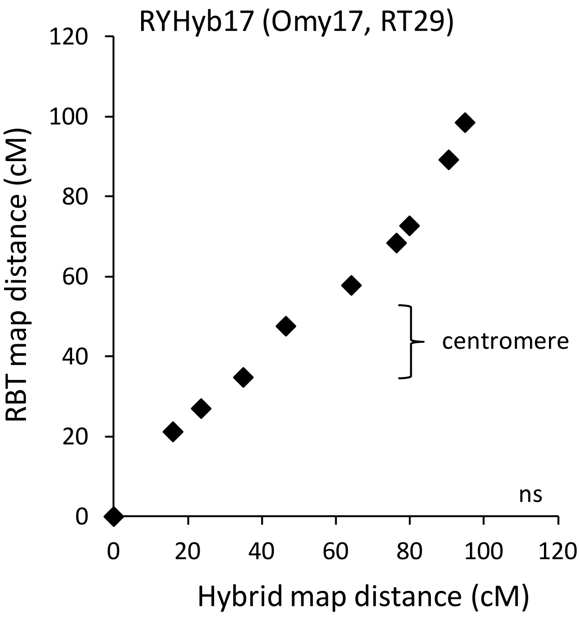
RYHyb17 (Omy17, RT29)
RBT map distance (cM)
Hybrid map distance (cM)
centromere
ns
0
20
40
60
80
100
120

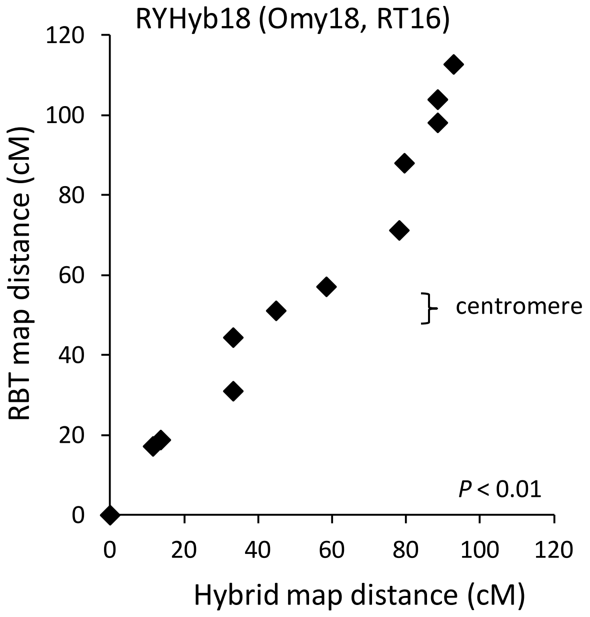
RYHyb18 (Omy18, RT16)
RBT map distance (cM)
Hybrid map distance (cM)
centromere
P < 0.01
0
20
40
60
80
100
120

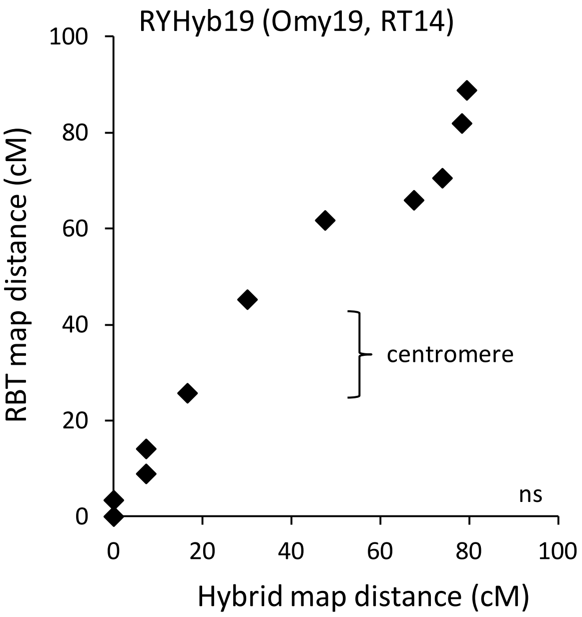

RYHyb19 (Omy19, RT14)
RBT map distance (cM)
Hybrid map distance (cM)
centromere
ns
0
20
40
60
80
100

Map distance comparisons for RYHyb20 were done across each RBT chromosomes that was fused in the hybrid map.

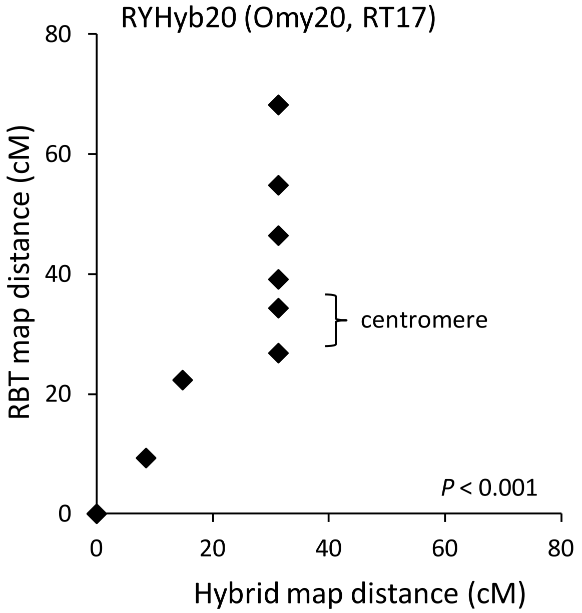

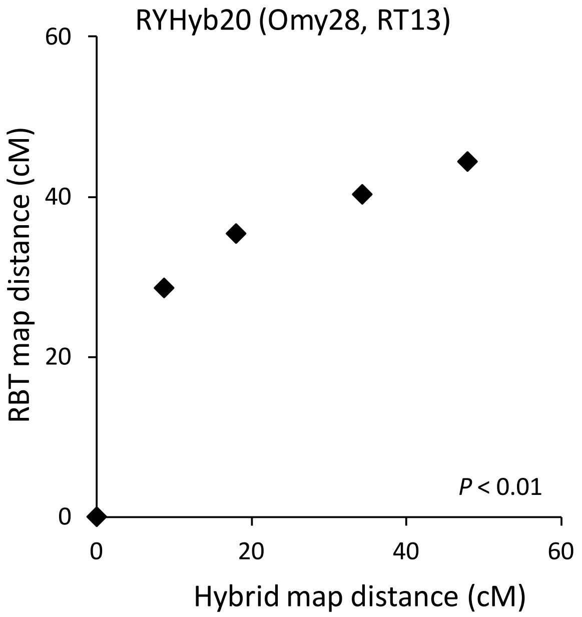

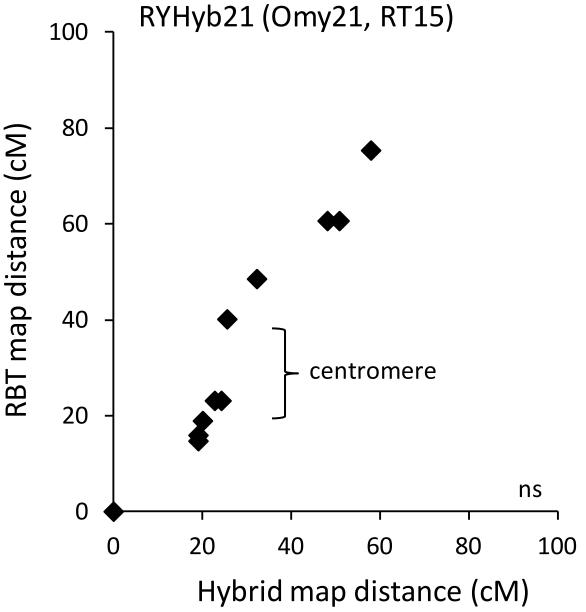

RYHyb21 (Omy21, RT15)
RBT map distance (cM)
Hybrid map distance (cM)
centromere
ns
0
20
40
60
80
100

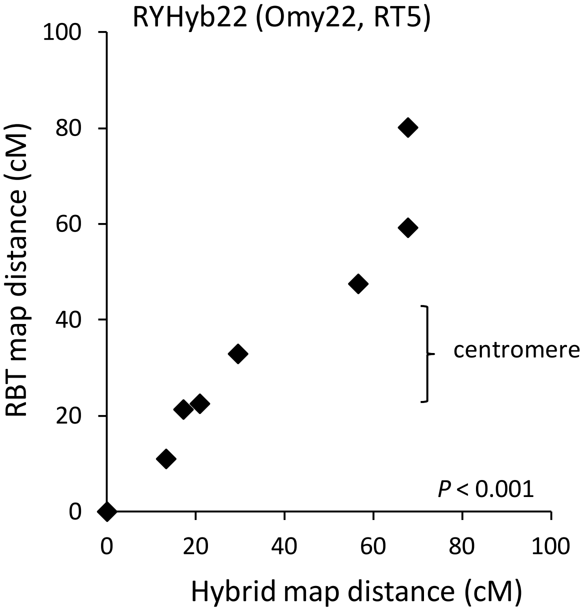

RYHyb22 (Omy22, RT5)
RBT map distance (cM)
Hybrid map distance (cM)
centromere
P < 0.001
0
20
40
60
80
100

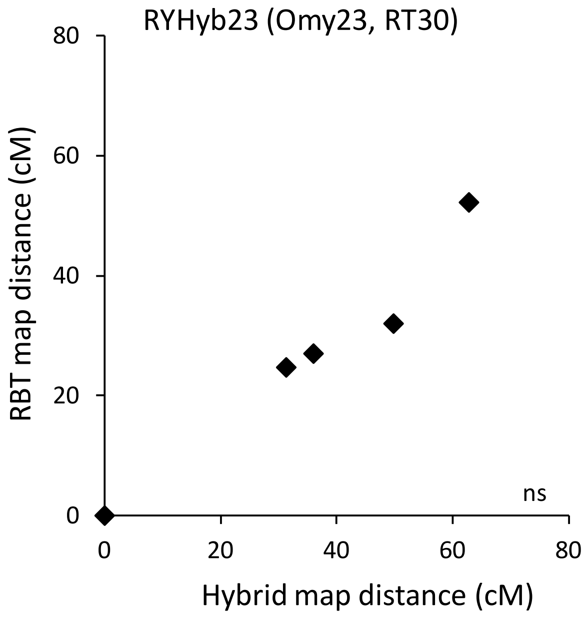
RYHyb23 (Omy23, RT30)
RBT map distance (cM)
Hybrid map distance (cM)
ns
0
20
40
60
80

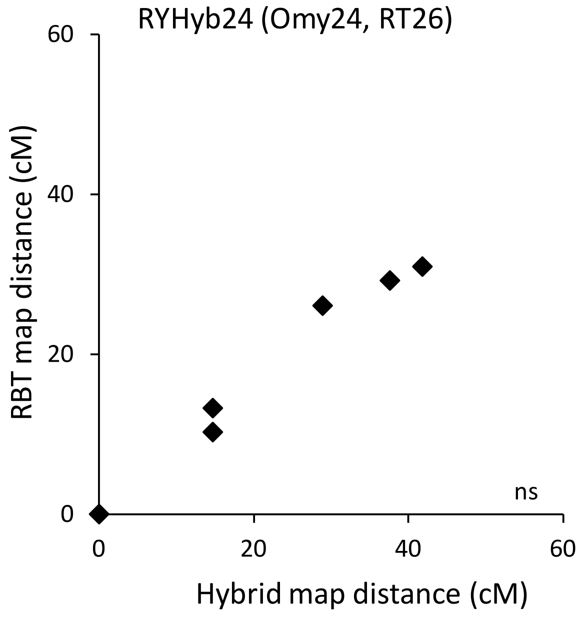
RYHyb24 (Omy24, RT26)
RBT map distance (cM)
Hybrid map distance (cM)
ns
0
20
40
60

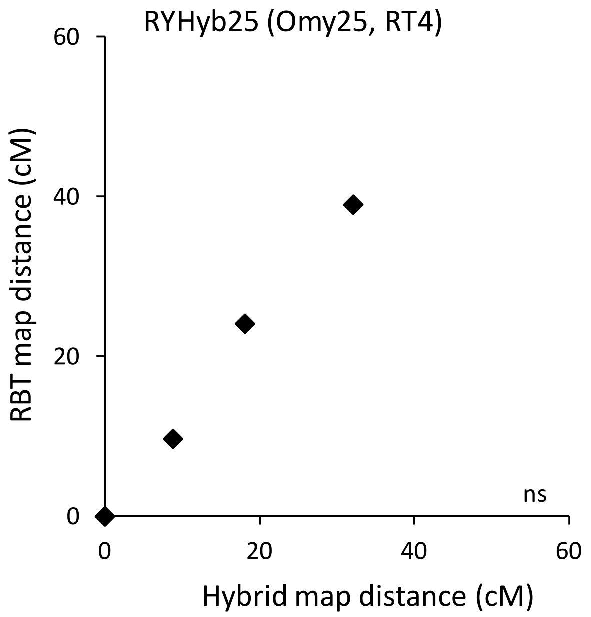
RYHyb25 (Omy25, RT4)
RBT map distance (cM)
Hybrid map distance (cM)
ns
0
20
40
60

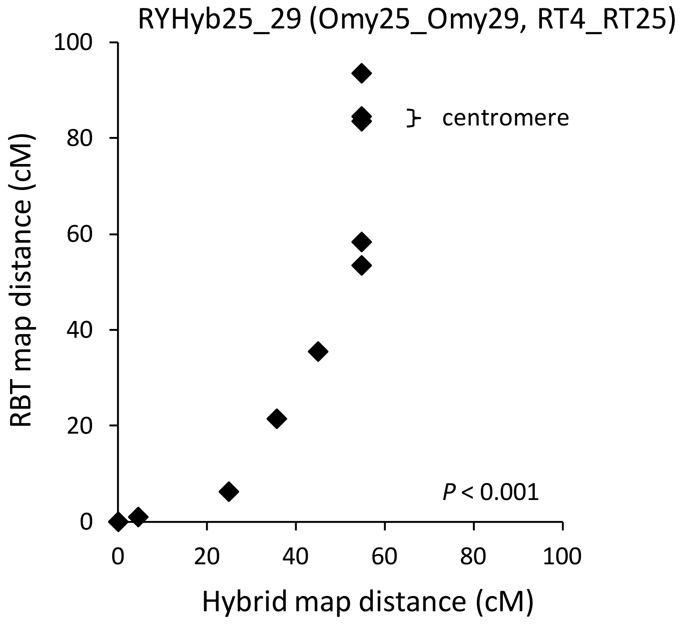
RYHyb25_29 (Omy25_Omy29, RT4_RT25)
RBT map distance (cM)
Hybrid map distance (cM)
centromere
P < 0.001
0
20
40
60
80
100

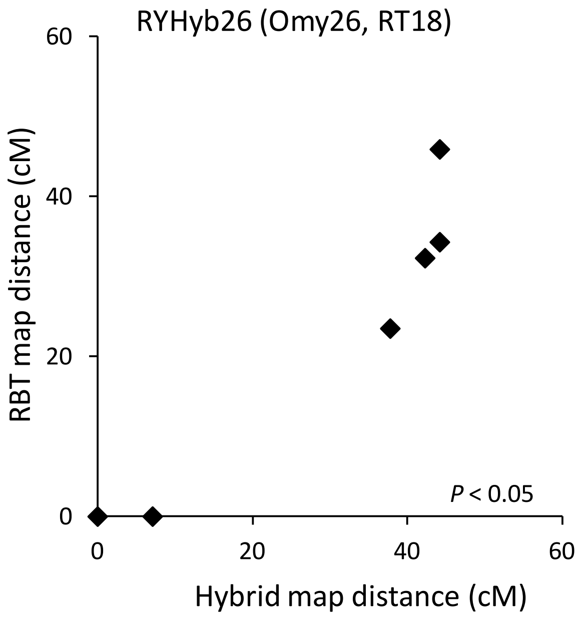
RYHyb26 (Omy26, RT18)
RBT map distance (cM)
Hybrid map distance (cM)
P < 0.05
0
20
40
60
0
20
40
60

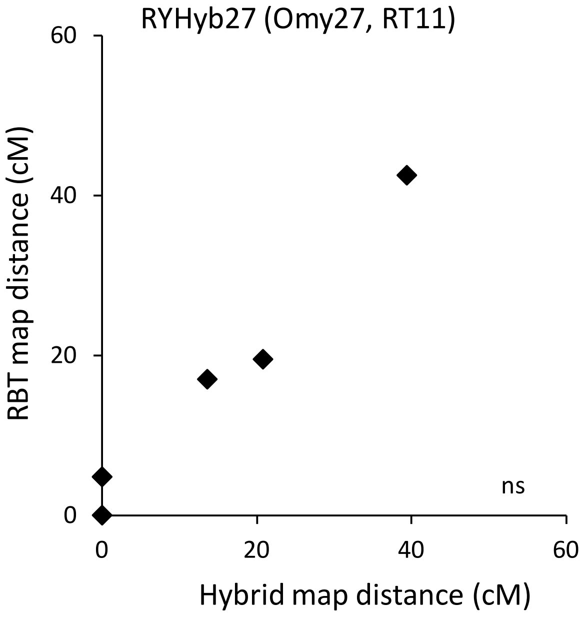
RYHyb27 (Omy27, RT11)
RBT map distance (cM)
Hybrid map distance (cM)
ns
0
20
40
60
0
20
40
60

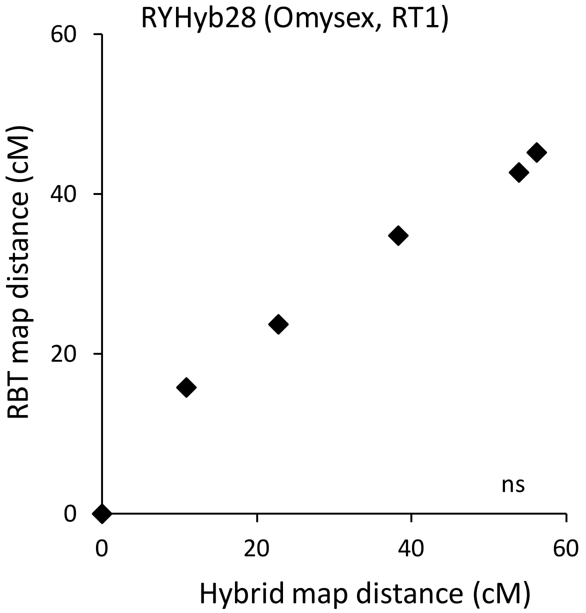
RYHyb28 (Omysex, RT1)
RBT map distance (cM)
Hybrid map distance (cM)
ns
0
20
40
60
0
20
40
60

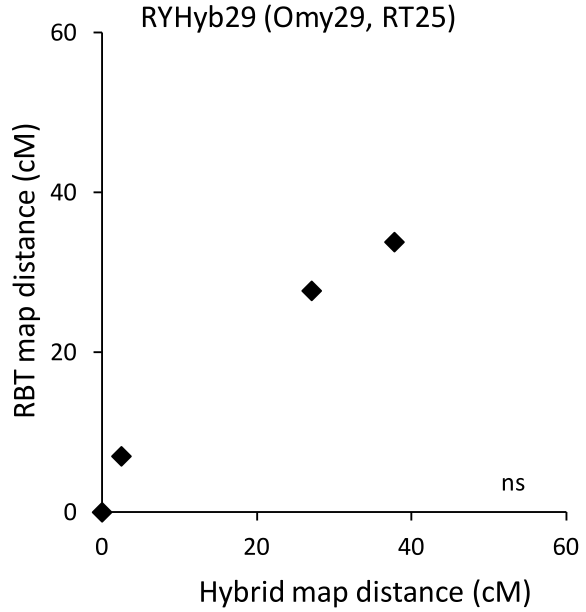
RYHyb29 (Omy29, RT25)
RBT map distance (cM)
Hybrid map distance (cM)
ns
0
20
40
60
0
20
40
60
