# Supplementary material for: Chromosome rearrangements, recombination suppression, and limited segregation distortion in hybrids between Yellowstone cutthroat trout (Oncorhynchus clarkii bouvieri) and rainbow trout (O. mykiss)
Source: BMC Genomics. 2013 Aug 22;14:570. doi: 10.1186/1471-2164-14-570 (PMC3765842; doi:10.1186/1471-2164-14-570)
Supplement: Additional file 5 — This PDF file includes figures representing inferred genotypic frequency distributions for Yellowstone cutthroat trout homozygotes, rainbow trout homozygotes, and heterozygotes at each locus in Family 1, Family 2, and loci scored in both families combined across each hybrid linkage group. [file 1471-2164-14-570-S5.pdf]

Additional file 5. Genotypic frequency distributions (Yellowstone cutthroat trout homozygotes, YCT/YCT; rainbow trout homozygotes, RBT/RBT; and heterozygotes, YCT/RBT) for loci in Family 1, Family 2, and loci scored in both families combined. Loci are presented in map order (*p* to *q* orientation), except those within stippled boxes which represent species markers and variable SNP markers for which mapping parents were heterozygous for the same alleles. These loci were assigned to linkage groups but not localized within linkage groups. * indicates Chi-square test where $P < 0.05$ with two degrees of freedom, and** indicates genotypic frequency distributions that differ significantly from expected distributions after applying a false discovery rate correction (B-Y FDR) for multiple tests.

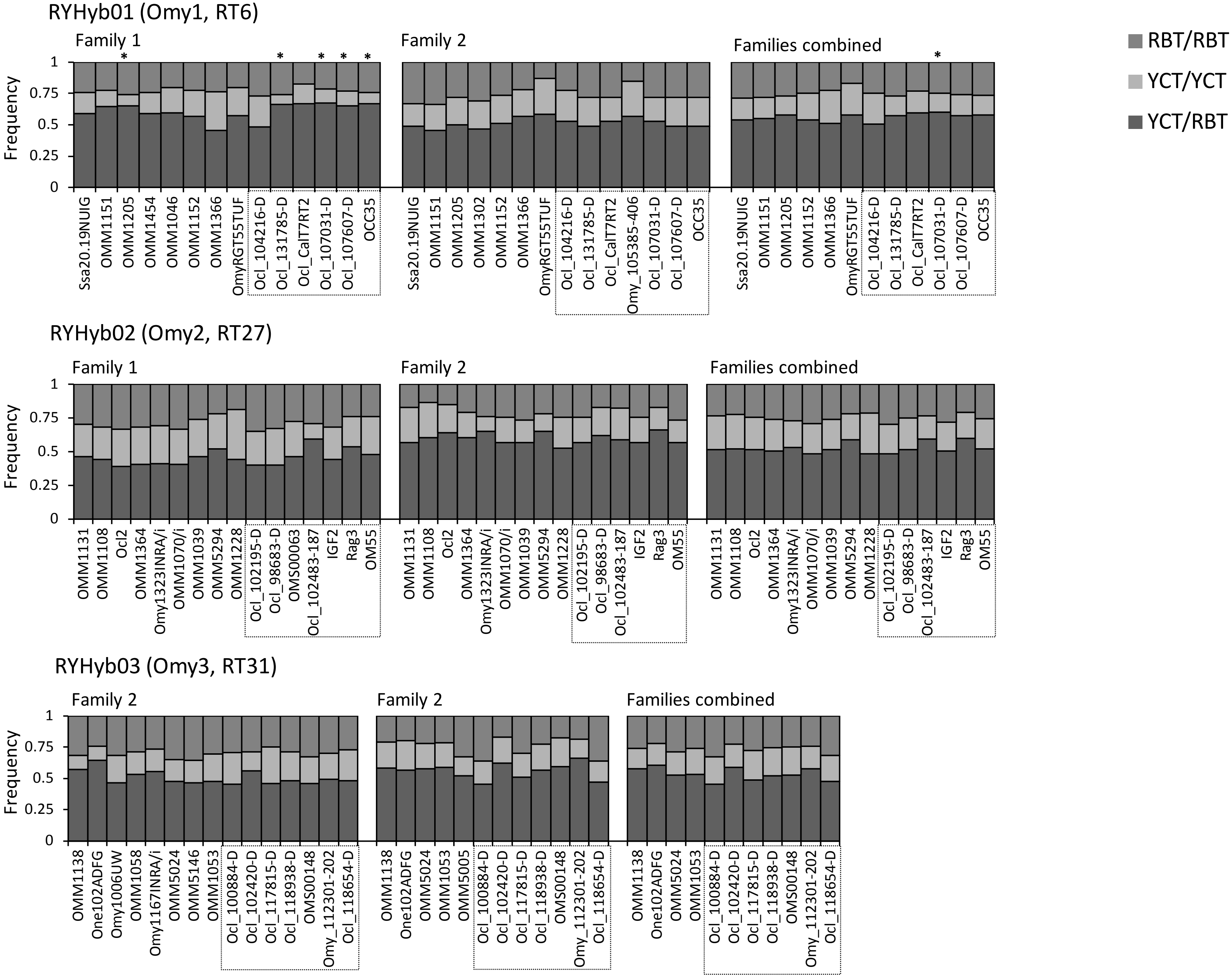
RYHyb01 (Omy1, RT6)
Family 1
Family 2
Families combined
RBT/RBT
YCT/YCT
YCT/RBT
Frequency
1
0.75
0.5
0.25
0
*
Ssa20.19NUIG
OMM1151
OMM1205
OMM1454
OMM1046
OMM1152
OMM1366
OmyRGT55TUF
Ocl_104216-D
Ocl_131785-D
Ocl_CalT7RT2
Ocl_107031-D
Ocl_107607-D
OCC35
OMM1302
Omy_105385-406
RYHyb02 (Omy2, RT27)
OMM1131
OMM1108
Ocl2
OMM1364
Omy1323INRA/i
OMM1070/i
OMM1039
OMM5294
OMM1228
Ocl_102195-D
Ocl_98683-D
OMS00063
Ocl_102483-187
IGF2
Rag3
OM55
RYHyb03 (Omy3, RT31)
OMM1138
One102ADFG
Omy1006UW
OMM1058
Omy1167INRA/i
OMM5024
OMM5146
OMM1053
OMM5005
Ocl_100884-D
Ocl_102420-D
Ocl_117815-D
Ocl_118938-D
OMS00148
Omy_112301-202
Ocl_118654-D

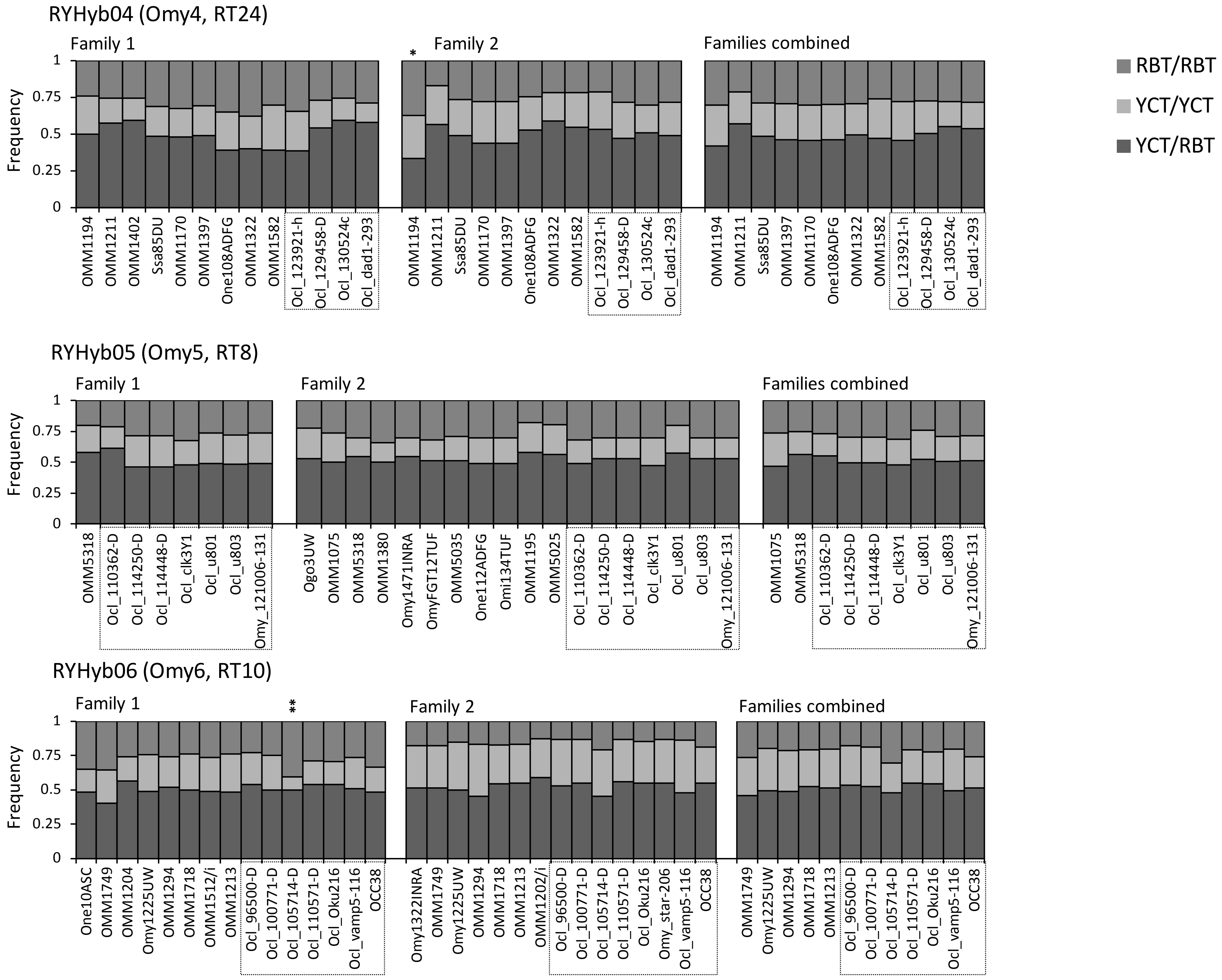

RYHyb04 (Omy4, RT24)
Family 1
Family 2
Families combined
RBT/RBT
YCT/YCT
YCT/RBT
Frequency
1
0.75
0.5
0.25
0
*
OMM1194
OMM1211
OMM1402
Ssa85DU
OMM1170
OMM1397
One108ADFG
OMM1322
OMM1582
Ocl_123921-h
Ocl_129458-D
Ocl_130524c
Ocl_dad1-293
OMM1194
OMM1211
Ssa85DU
OMM1170
OMM1397
One108ADFG
OMM1322
OMM1582
Ocl_123921-h
Ocl_129458-D
Ocl_130524c
Ocl_dad1-293
OMM1194
OMM1211
Ssa85DU
OMM1397
OMM1170
One108ADFG
OMM1322
OMM1582
Ocl_123921-h
Ocl_129458-D
Ocl_130524c
Ocl_dad1-293
RYHyb05 (Omy5, RT8)
Family 1
Family 2
Families combined
Frequency
OMM5318
Ocl_110362-D
Ocl_114250-D
Ocl_114448-D
Ocl_clk3Y1
Ocl_u801
Ocl_u803
Omy_121006-131
Ogo3UW
OMM1075
OMM5318
OMM1380
Omy1471INRA
OmyFGT12TUF
OMM5035
One112ADFG
Omi134TUF
OMM1195
OMM5025
Ocl_110362-D
Ocl_114250-D
Ocl_114448-D
Ocl_clk3Y1
Ocl_u801
Ocl_u803
Omy_121006-131
OMM1075
OMM5318
Ocl_110362-D
Ocl_114250-D
Ocl_114448-D
Ocl_clk3Y1
Ocl_u801
Ocl_u803
Omy_121006-131
RYHyb06 (Omy6, RT10)
Family 1
Family 2
Families combined
**
Frequency
One10ASC
OMM1749
OMM1204
Omy1225UW
OMM1294
OMM1718
OMM1512/i
OMM1213
Ocl_96500-D
Ocl_100771-D
Ocl_105714-D
Ocl_110571-D
Ocl_Oku216
Ocl_vamp5-116
OCC38
Omy1322INRA
OMM1749
Omy1225UW
OMM1294
OMM1718
OMM1213
OMM1202/i
Ocl_96500-D
Ocl_100771-D
Ocl_105714-D
Ocl_110571-D
Ocl_Oku216
Omy_star-206
Ocl_vamp5-116
OCC38
OMM1749
Omy1225UW
OMM1294
OMM1718
OMM1213
Ocl_96500-D
Ocl_100771-D
Ocl_105714-D
Ocl_110571-D
Ocl_Oku216
Ocl_vamp5-116
OCC38

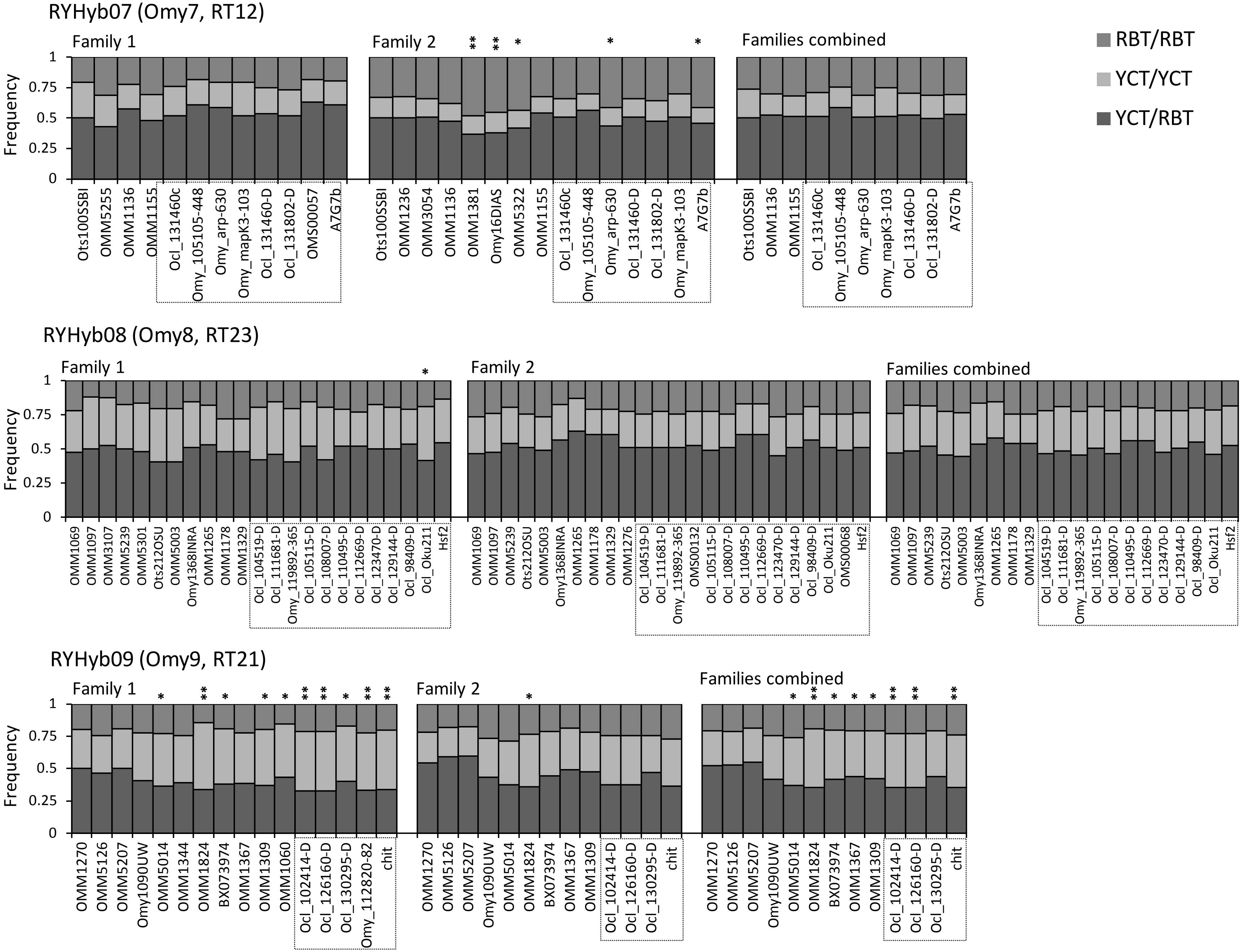
RYHyb07 (Omy7, RT12)
Family 1
Family 2
Families combined
RBT/RBT
YCT/YCT
YCT/RBT
Frequency
1
0.75
0.5
0.25
0
Ots100SSBI
OMM5255
OMM1136
OMM1155
Ocl_131460c
Omy_105105-448
Omy_arp-630
Omy_mapK3-103
Ocl_131460-D
Ocl_131802-D
OMS00057
A7G7b
OMM1236
OMM3054
OMM1381
Omy16DIAS
OMM5322
RYHyb08 (Omy8, RT23)
OMM1069
OMM1097
OMM3107
OMM5239
OMM5301
Ots212OSU
OMM5003
Omy1368INRA
OMM1265
OMM1178
OMM1329
OMM1276
Ocl_104519-D
Ocl_111681-D
Omy_119892-365
OMS00132
Ocl_105115-D
Ocl_108007-D
Ocl_110495-D
Ocl_112669-D
Ocl_123470-D
Ocl_129144-D
Ocl_98409-D
Ocl_Oku211
OMS00068
Hsf2
RYHyb09 (Omy9, RT21)
OMM1270
OMM5126
OMM5207
Omy1090UW
OMM5014
OMM1344
OMM1824
BX073974
OMM1367
OMM1309
OMM1060
Ocl_102414-D
Ocl_126160-D
Ocl_130295-D
Omy_112820-82
chit

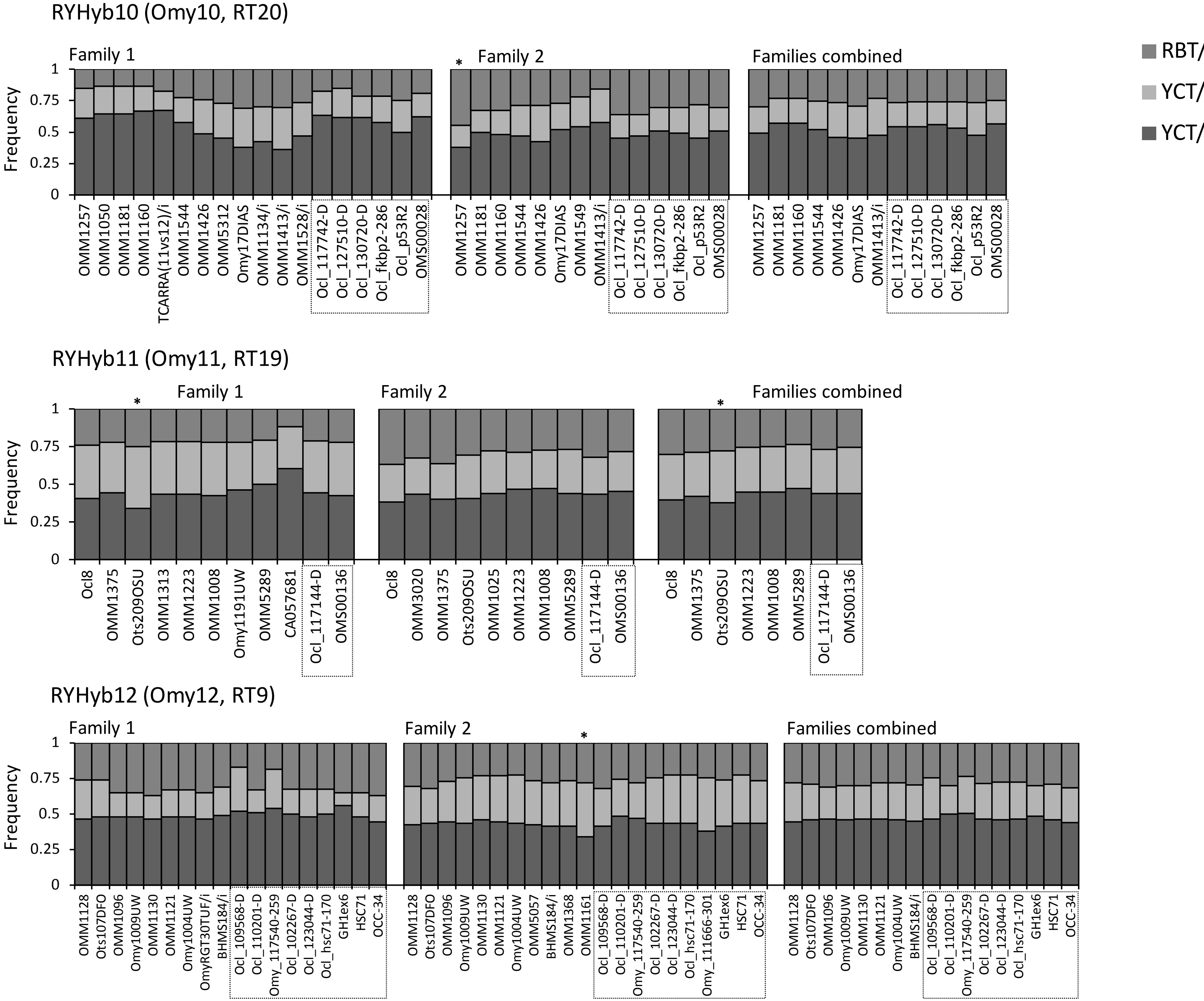
RYHyb10 (Omy10, RT20)
Family 1
Family 2
Families combined
RBT/RBT
YCT/YCT
YCT/RBT
Frequency
OMM1257
OMM1050
OMM1181
OMM1160
TCARRA(11vs12)/i
OMM1544
OMM1426
OMM5312
Omy17DIAS
OMM1134/i
OMM1413/i
OMM1528/i
Ocl_117742-D
Ocl_127510-D
Ocl_130720-D
Ocl_fkbp2-286
Ocl_p53R2
OMS00028
OMM1549
RYHyb11 (Omy11, RT19)
Ocl8
OMM1375
Ots209OSU
OMM1313
OMM1223
OMM1008
Omy1191UW
OMM5289
CA057681
Ocl_117144-D
OMS00136
OMM3020
OMM1025
RYHyb12 (Omy12, RT9)
OMM1128
Ots107DFO
OMM1096
Omy1009UW
OMM1130
OMM1121
Omy1004UW
OmyRGT30TUF/i
BHMS184/i
Ocl_109568-D
Ocl_110201-D
Omy_117540-259
Ocl_102267-D
Ocl_123044-D
Ocl_hsc71-170
GH1ex6
HSC71
OCC-34
OMM5057
OMM1368
OMM1161
Omy_111666-301

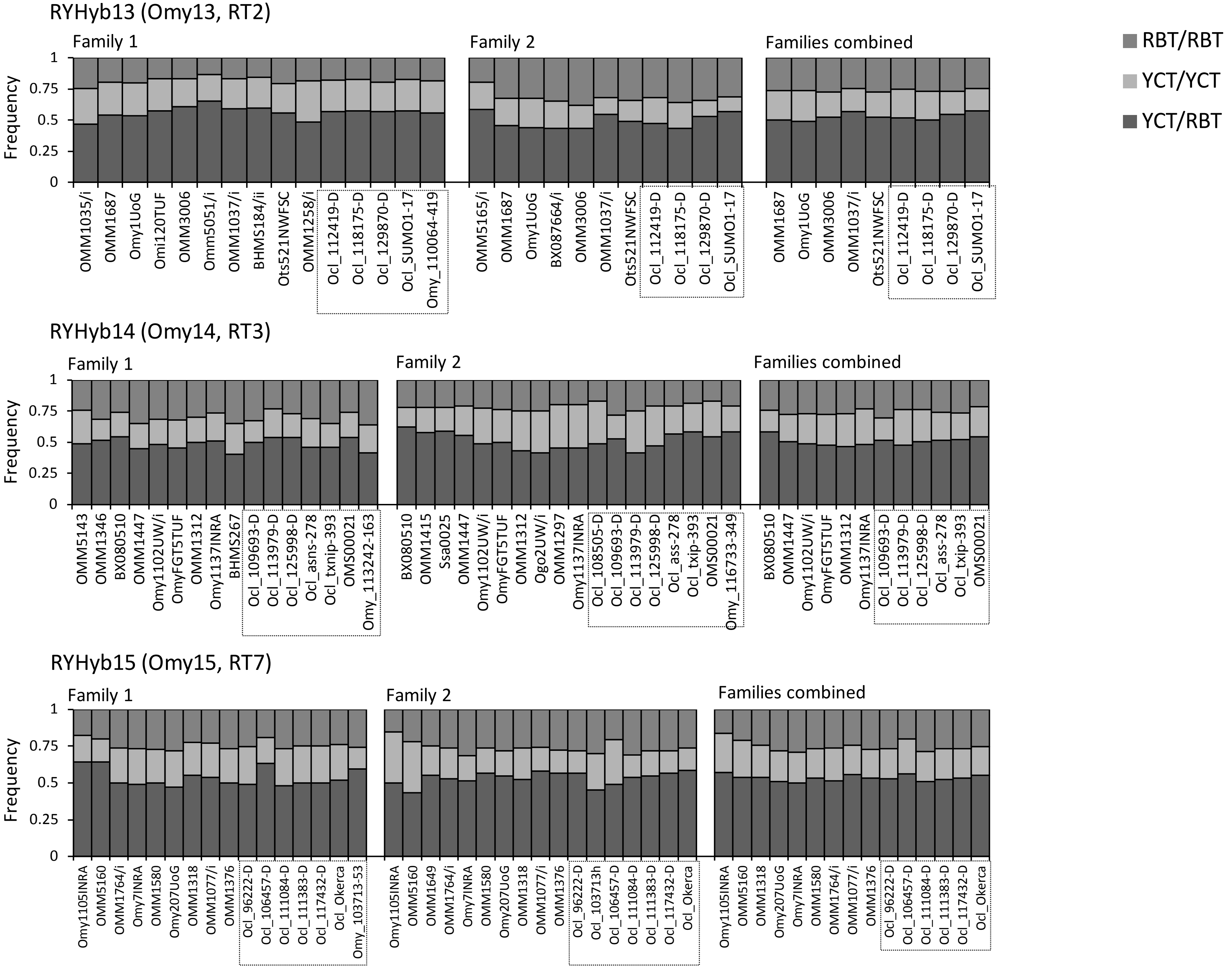
RYHyb13 (Omy13, RT2)
Family 1
Family 2
Families combined
RBT/RBT
YCT/YCT
YCT/RBT
Frequency
0
0.25
0.5
0.75
1
OMM1035/i
OMM1687
Omy1UoG
Omi120TUF
OMM3006
Omm5051/i
OMM1037/i
BHMS184/ii
Ots521NWFSC
OMM1258/i
Ocl_112419-D
Ocl_118175-D
Ocl_129870-D
Ocl_SUMO1-17
Omy_110064-419
OMM5165/i
OMM1687
Omy1UoG
BX087664/i
OMM3006
OMM1037/i
Ots521NWFSC
Ocl_112419-D
Ocl_118175-D
Ocl_129870-D
Ocl_SUMO1-17
OMM1687
Omy1UoG
OMM3006
OMM1037/i
Ots521NWFSC
Ocl_112419-D
Ocl_118175-D
Ocl_129870-D
Ocl_SUMO1-17
RYHyb14 (Omy14, RT3)
Family 1
Family 2
Families combined
Frequency
0
0.25
0.5
0.75
1
OMM5143
OMM1346
BX080510
OMM1447
Omy1102UW/i
OmyFGT5TUF
OMM1312
Omy1137INRA
BHMS267
Ocl_109693-D
Ocl_113979-D
Ocl_125998-D
Ocl_asns-278
Ocl_txnip-393
OMS00021
Omy_113242-163
BX080510
OMM1415
Ssa0025
OMM1447
Omy1102UW/i
OmyFGT5TUF
OMM1312
Ogo2UW/i
OMM1297
Omy1137INRA
Ocl_108505-D
Ocl_109693-D
Ocl_113979-D
Ocl_125998-D
Ocl_ass-278
Ocl_txip-393
OMS00021
Omy_116733-349
BX080510
OMM1447
Omy1102UW/i
OmyFGT5TUF
OMM1312
Omy1137INRA
Ocl_109693-D
Ocl_113979-D
Ocl_125998-D
Ocl_ass-278
Ocl_txip-393
OMS00021
RYHyb15 (Omy15, RT7)
Family 1
Family 2
Families combined
Frequency
0
0.25
0.5
0.75
1
Omy1105INRA
OMM5160
OMM1764/i
Omy7INRA
OMM1580
Omy207UoG
OMM1318
OMM1077/i
OMM1376
Ocl_96222-D
Ocl_106457-D
Ocl_111084-D
Ocl_111383-D
Ocl_117432-D
Ocl_Okerca
Omy_103713-53
Omy1105INRA
OMM5160
OMM1649
OMM1764/i
Omy7INRA
OMM1580
Omy207UoG
OMM1318
OMM1077/i
OMM1376
Ocl_96222-D
Ocl_103713h
Ocl_106457-D
Ocl_111084-D
Ocl_111383-D
Ocl_117432-D
Ocl_Okerca
Omy1105INRA
OMM5160
OMM1318
Omy207UoG
Omy7INRA
OMM1580
OMM1764/i
OMM1077/i
OMM1376
Ocl_96222-D
Ocl_106457-D
Ocl_111084-D
Ocl_111383-D
Ocl_117432-D
Ocl_Okerca

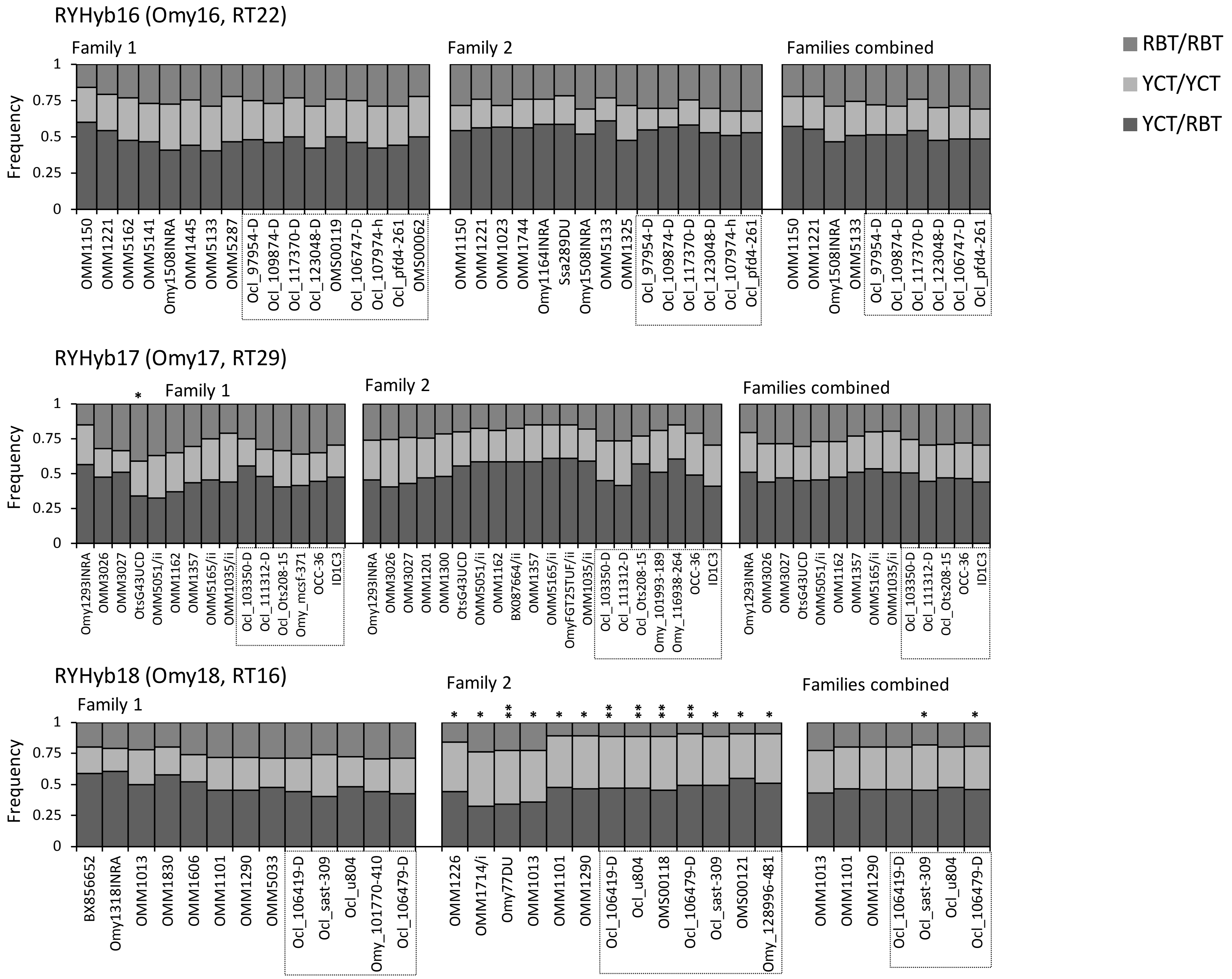
RYHyb16 (Omy16, RT22)
Family 1
Family 2
Families combined
RBT/RBT
YCT/YCT
YCT/RBT
Frequency
0
0.25
0.5
0.75
1
OMM1150
OMM1221
OMM5162
OMM5141
Omy1508INRA
OMM1445
OMM5133
OMM5287
Ocl_97954-D
Ocl_109874-D
Ocl_117370-D
Ocl_123048-D
OMS00119
Ocl_106747-D
Ocl_107974-h
Ocl_pfd4-261
OMS00062
OMM1150
OMM1221
OMM1023
OMM1744
Omy1164INRA
Ssa289DU
Omy1508INRA
OMM5133
OMM1325
Ocl_97954-D
Ocl_109874-D
Ocl_117370-D
Ocl_123048-D
Ocl_107974-h
Ocl_pfd4-261
OMM1150
OMM1221
Omy1508INRA
OMM5133
Ocl_97954-D
Ocl_109874-D
Ocl_117370-D
Ocl_123048-D
Ocl_106747-D
Ocl_pfd4-261
RYHyb17 (Omy17, RT29)
Family 1
Family 2
Families combined
Omy1293INRA
OMM3026
OMM3027
OtsG43UCD
OMM5051/ii
OMM1162
OMM1357
OMM5165/ii
OMM1035/ii
Ocl_103350-D
Ocl_111312-D
Ocl_Ots208-15
Omy_mcsf-371
OCC-36
ID1C3
Omy1293INRA
OMM3026
OMM3027
OMM1201
OMM1300
OtsG43UCD
OMM5051/ii
OMM1162
BX087664/ii
OMM1357
OMM5165/ii
OmyFGT25TUF/ii
OMM1035/ii
Ocl_103350-D
Ocl_111312-D
Ocl_Ots208-15
Omy_101993-189
Omy_116938-264
OCC-36
ID1C3
Omy1293INRA
OMM3026
OMM3027
OtsG43UCD
OMM5051/ii
OMM1162
OMM1357
OMM5165/ii
OMM1035/ii
Ocl_103350-D
Ocl_111312-D
Ocl_Ots208-15
OCC-36
ID1C3
RYHyb18 (Omy18, RT16)
Family 1
Family 2
Families combined
BX856652
Omy1318INRA
OMM1013
OMM1830
OMM1606
OMM1101
OMM1290
OMM5033
Ocl_106419-D
Ocl_sast-309
Ocl_u804
Omy_101770-410
Ocl_106479-D
OMM1226
OMM1714/i
Omy77DU
OMM1013
OMM1101
OMM1290
Ocl_106419-D
Ocl_u804
OMS00118
Ocl_106479-D
Ocl_sast-309
OMS00121
Omy_128996-481
OMM1013
OMM1101
OMM1290
Ocl_106419-D
Ocl_sast-309
Ocl_u804
Ocl_106479-D

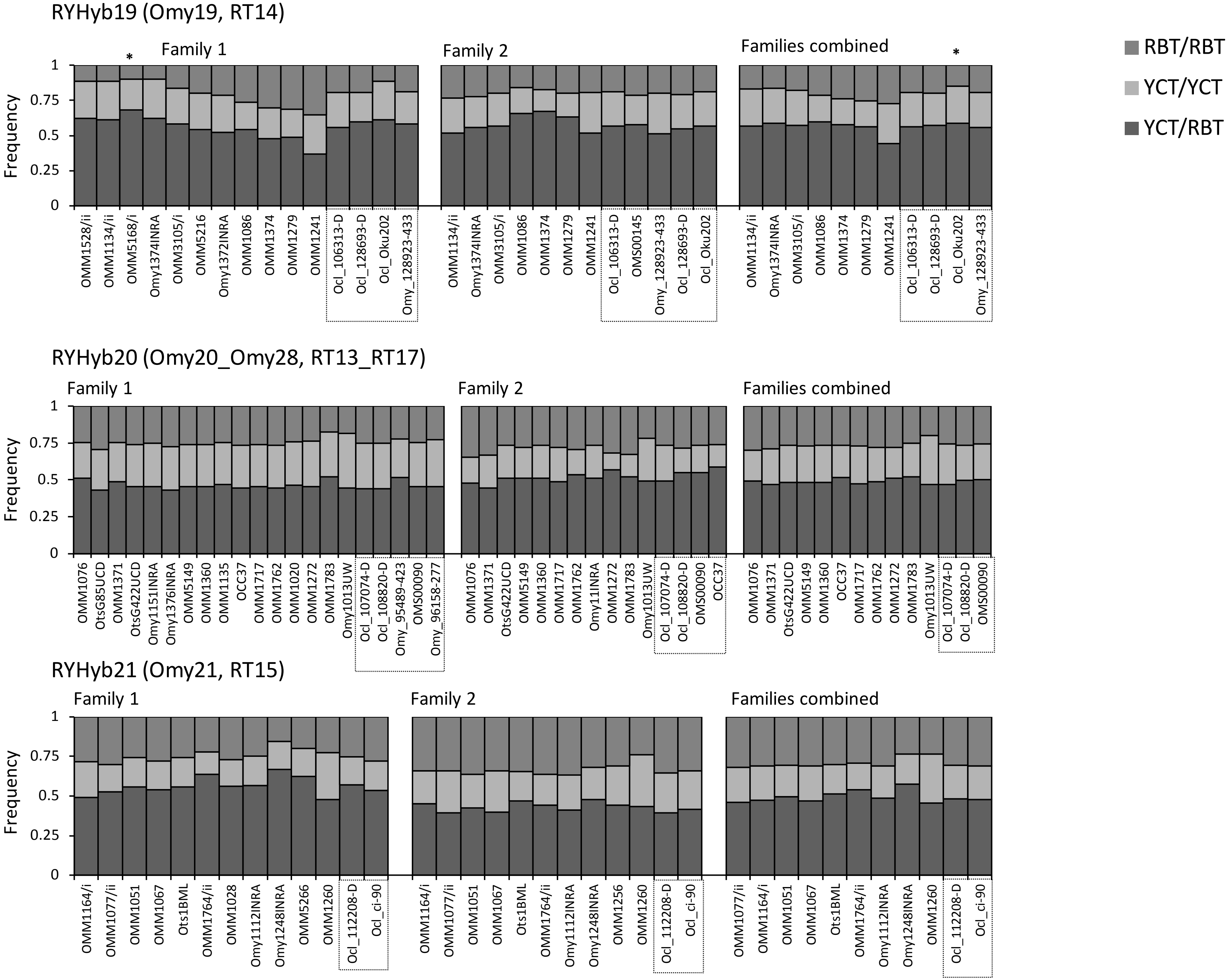
RYHyb19 (Omy19, RT14)
Family 1
Family 2
Families combined
RBT/RBT
YCT/YCT
YCT/RBT
Frequency
1
0.75
0.5
0.25
0
*
OMM1528/ii
OMM1134/ii
OMM5168/i
Omy1374INRA
OMM3105/i
OMM5216
Omy1372INRA
OMM1086
OMM1374
OMM1279
OMM1241
Ocl_106313-D
Ocl_128693-D
Ocl_Oku202
Omy_128923-433
OMM1134/ii
Omy1374INRA
OMM3105/i
OMM1086
OMM1374
OMM1279
OMM1241
Ocl_106313-D
OMS00145
Omy_128923-433
Ocl_128693-D
Ocl_Oku202
OMM1134/ii
Omy1374INRA
OMM3105/i
OMM1086
OMM1374
OMM1279
OMM1241
Ocl_106313-D
Ocl_128693-D
Ocl_Oku202
Omy_128923-433
RYHyb20 (Omy20_Omy28, RT13_RT17)
Family 1
Family 2
Families combined
OMM1076
OtsG85UCD
OMM1371
OtsG422UCD
Omy1151INRA
Omy1376INRA
OMM5149
OMM1360
OMM1135
OCC37
OMM1717
OMM1762
OMM1020
OMM1272
OMM1783
Omy1013UW
Ocl_107074-D
Ocl_108820-D
Omy_95489-423
OMS00090
Omy_96158-277
OMM1076
OMM1371
OtsG422UCD
OMM5149
OMM1360
OMM1717
OMM1762
Omy11INRA
OMM1272
OMM1783
Omy1013UW
Ocl_107074-D
Ocl_108820-D
OMS00090
OCC37
OMM1076
OMM1371
OtsG422UCD
OMM5149
OMM1360
OCC37
OMM1717
OMM1762
OMM1272
OMM1783
Omy1013UW
Ocl_107074-D
Ocl_108820-D
OMS00090
RYHyb21 (Omy21, RT15)
Family 1
Family 2
Families combined
OMM1164/i
OMM1077/ii
OMM1051
OMM1067
Ots1BML
OMM1764/ii
OMM1028
Omy1112INRA
Omy1248INRA
OMM5266
OMM1260
Ocl_112208-D
Ocl_ci-90
OMM1164/i
OMM1077/ii
OMM1051
OMM1067
Ots1BML
OMM1764/ii
Omy1112INRA
Omy1248INRA
OMM1256
OMM1260
Ocl_112208-D
Ocl_ci-90
OMM1077/ii
OMM1164/i
OMM1051
OMM1067
Ots1BML
OMM1764/ii
Omy1112INRA
Omy1248INRA
OMM1260
Ocl_112208-D
Ocl_ci-90

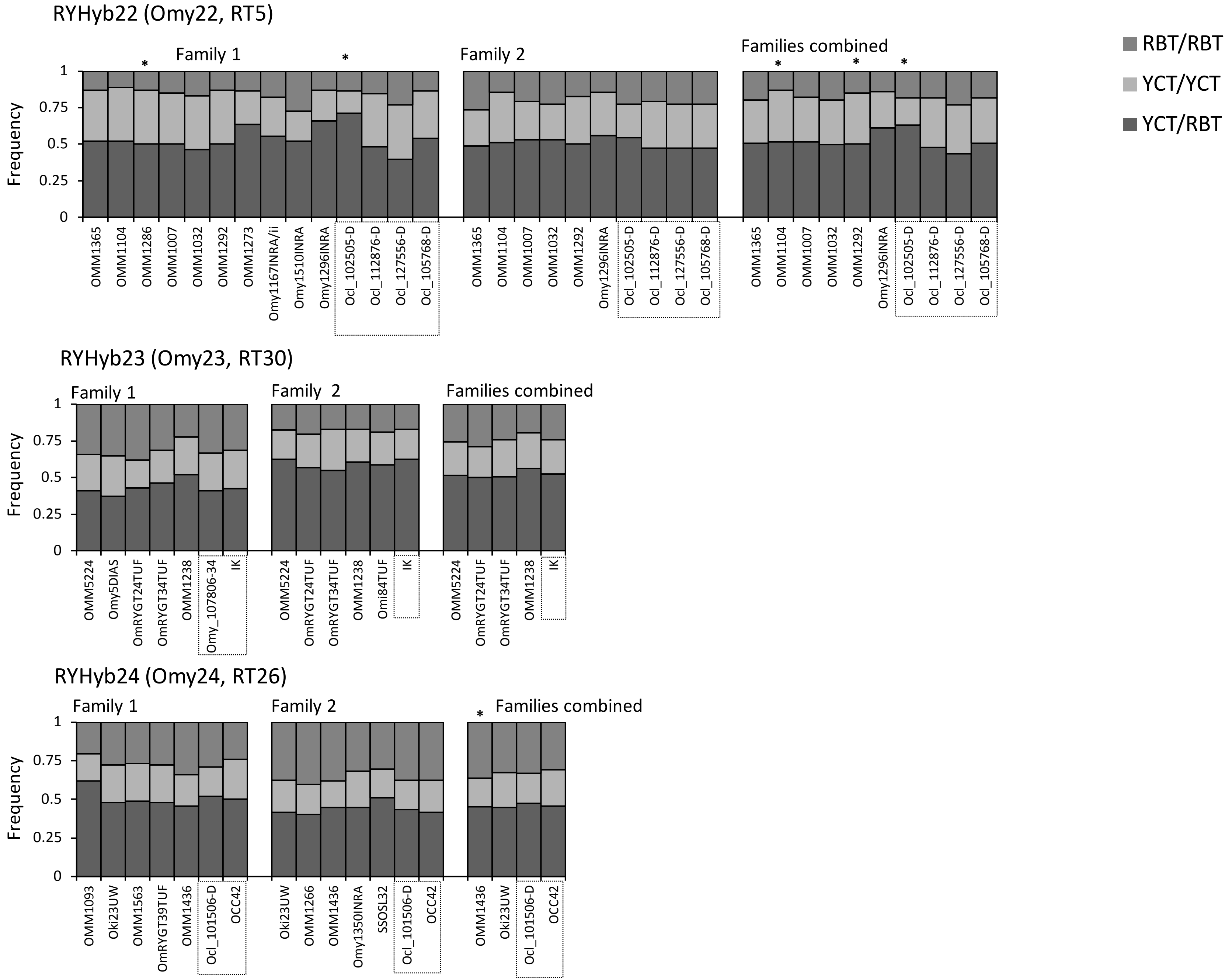
RYHyb22 (Omy22, RT5)
Family 1
Family 2
Families combined
RBT/RBT
YCT/YCT
YCT/RBT
Frequency
1
0.75
0.5
0.25
0
*
OMM1365
OMM1104
OMM1286
OMM1007
OMM1032
OMM1292
OMM1273
Omy1167INRA/ii
Omy1510INRA
Omy1296INRA
Ocl_102505-D
Ocl_112876-D
Ocl_127556-D
Ocl_105768-D
OMM1365
OMM1104
OMM1007
OMM1032
OMM1292
Omy1296INRA
Ocl_102505-D
Ocl_112876-D
Ocl_127556-D
Ocl_105768-D
OMM1365
OMM1104
OMM1007
OMM1032
OMM1292
Omy1296INRA
Ocl_102505-D
Ocl_112876-D
Ocl_127556-D
Ocl_105768-D
RYHyb23 (Omy23, RT30)
Family 1
Family 2
Families combined
Frequency
OMM5224
Omy5DIAS
OmRYGT24TUF
OmRYGT34TUF
OMM1238
Omy_107806-34
IK
OMM5224
OmRYGT24TUF
OmRYGT34TUF
OMM1238
Omi84TUF
IK
OMM5224
OmRYGT24TUF
OmRYGT34TUF
OMM1238
IK
RYHyb24 (Omy24, RT26)
Family 1
Family 2
Families combined
Frequency
OMM1093
Oki23UW
OMM1563
OmRYGT39TUF
OMM1436
Ocl_101506-D
OCC42
Oki23UW
OMM1266
OMM1436
Omy1350INRA
SSOSL32
Ocl_101506-D
OCC42
OMM1436
Oki23UW
Ocl_101506-D
OCC42

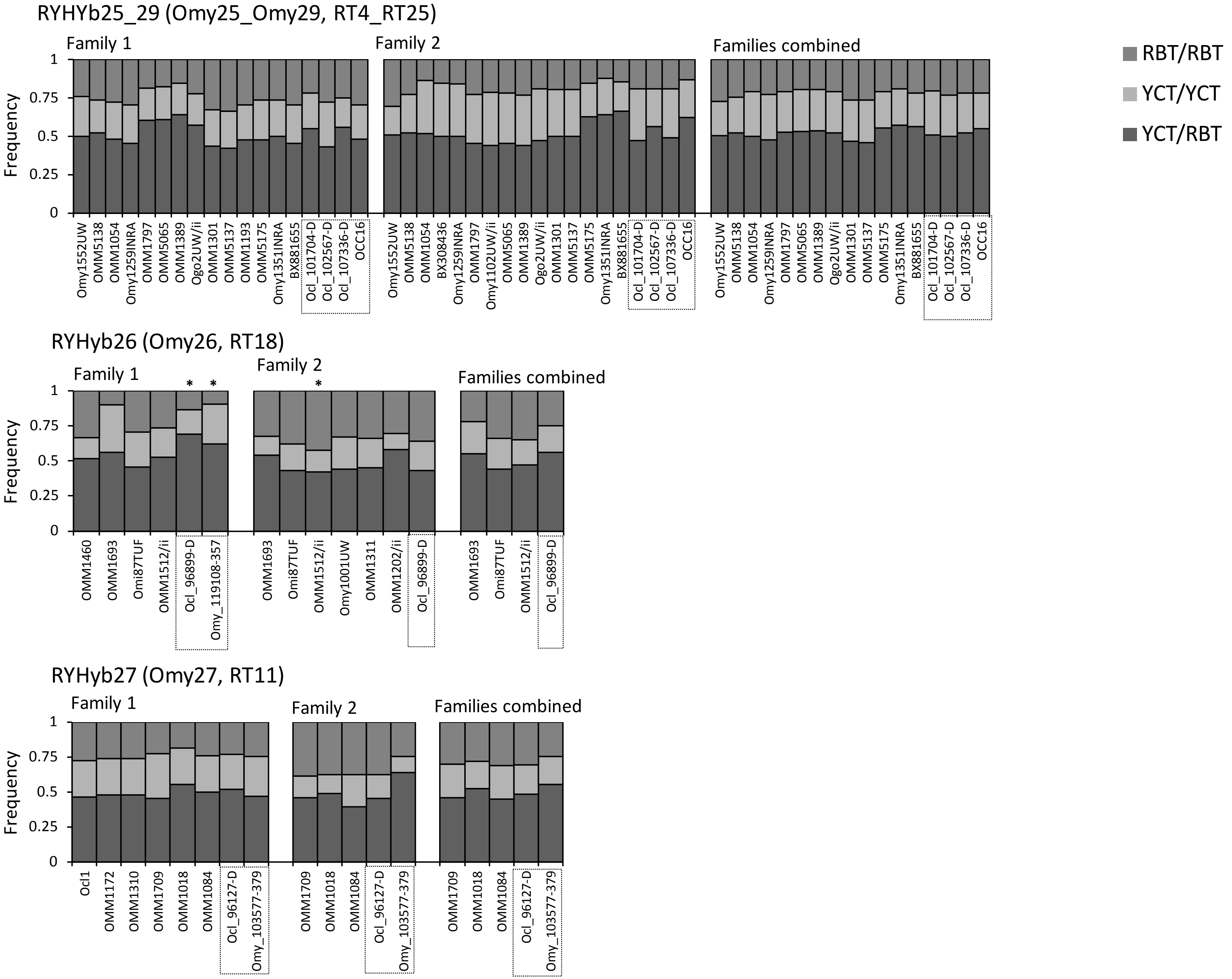

RYHYb25_29 (Omy25_Omy29, RT4_RT25)
Family 1
Family 2
Families combined
RBT/RBT
YCT/YCT
YCT/RBT
Frequency
1
0.75
0.5
0.25
0
Omy1552UW
OMM5138
OMM1054
Omy1259INRA
OMM1797
OMM5065
OMM1389
Ogo2UW/ii
OMM1301
OMM5137
OMM1193
OMM5175
Omy1351INRA
BX881655
Ocl_101704-D
Ocl_102567-D
Ocl_107336-D
OCC16
Omy1552UW
OMM5138
OMM1054
BX308436
Omy1259INRA
OMM1797
Omy1102UW/ii
OMM5065
OMM1389
Ogo2UW/ii
OMM1301
OMM5137
OMM5175
Omy1351INRA
BX881655
Ocl_101704-D
Ocl_102567-D
Ocl_107336-D
OCC16
Omy1552UW
OMM5138
OMM1054
Omy1259INRA
OMM1797
OMM5065
OMM1389
Ogo2UW/ii
OMM1301
OMM5137
OMM5175
Omy1351INRA
BX881655
Ocl_101704-D
Ocl_102567-D
Ocl_107336-D
OCC16
RYHyb26 (Omy26, RT18)
Family 1
Family 2
Families combined
*
*
*
Frequency
1
0.75
0.5
0.25
0
OMM1460
OMM1693
Omi87TUF
OMM1512/ii
Ocl_96899-D
Omy_119108-357
OMM1693
Omi87TUF
OMM1512/ii
Omy1001UW
OMM1311
OMM1202/ii
Ocl_96899-D
OMM1693
Omi87TUF
OMM1512/ii
Ocl_96899-D
RYHyb27 (Omy27, RT11)
Family 1
Family 2
Families combined
Frequency
1
0.75
0.5
0.25
0
Ocl1
OMM1172
OMM1310
OMM1709
OMM1018
OMM1084
Ocl_96127-D
Omy_103577-379
OMM1709
OMM1018
OMM1084
Ocl_96127-D
Omy_103577-379
OMM1709
OMM1018
OMM1084
Ocl_96127-D
Omy_103577-379

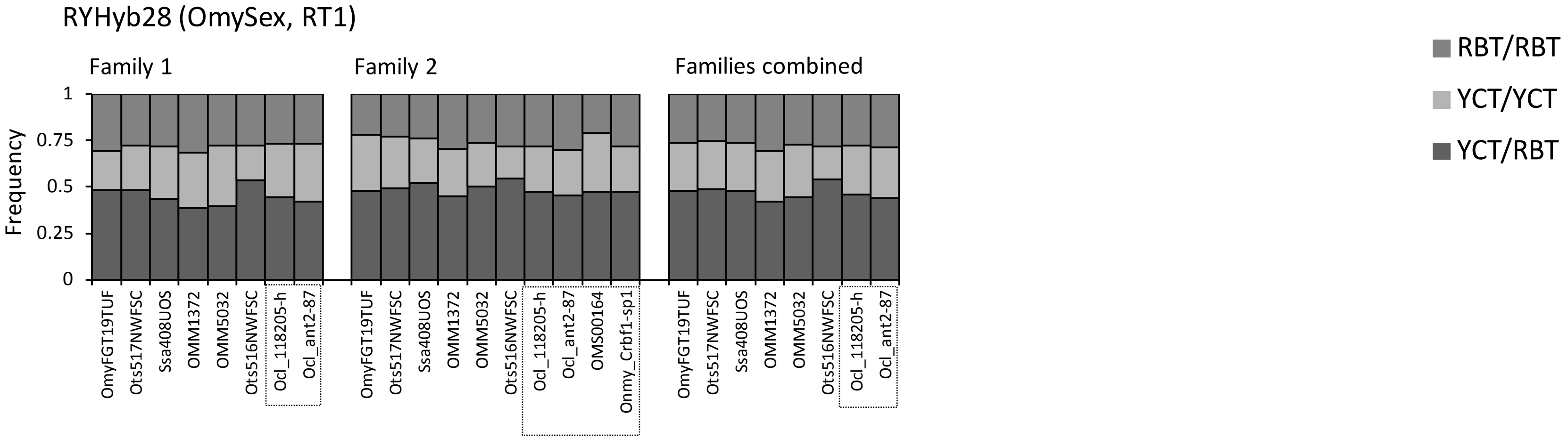
RYHyb28 (OmySex, RT1)
Family 1
Family 2
Families combined
RBT/RBT
YCT/YCT
YCT/RBT
Frequency
1
0.75
0.5
0.25
0
OmyFGT19TUF
Ots517NWFSC
Ssa408UOS
OMM1372
OMM5032
Ots516NWFSC
Ocl_118205-h
Ocl_ant2-87
OMS00164
Onmy_Crbf1-sp1
